# Modeling Heterogeneous Catalysis Using Quantum Computers: An Academic and Industry Perspective

**DOI:** 10.1021/acs.jcim.4c01212

**Published:** 2024-11-29

**Authors:** Seenivasan Hariharan, Sachin Kinge, Lucas Visscher

**Affiliations:** † Institute for Theoretical Physics, University of Amsterdam, Science Park 904, 1098 XH Amsterdam, The Netherlands; ‡ QuSoft, Science Park 123, 1098 XG Amsterdam, The Netherlands; ¶ Toyota Motor Europe, Materials Engineering Division, Hoge Wei 33, B-1930 Zaventum, Belgium; § Theoretical Chemistry, Vrije Universiteit, De Boelelaan 1083, 1081 HV Amsterdam, The Netherlands

**Keywords:** heterogeneous catalysis modeling, density functional theory, quantum computing algorithms, variational quantum algorithms, uncertainty quantification, embedding techniques, quantum-centric computing, magnetic catalysts, strong correlation effects, spin-related phenomena

## Abstract

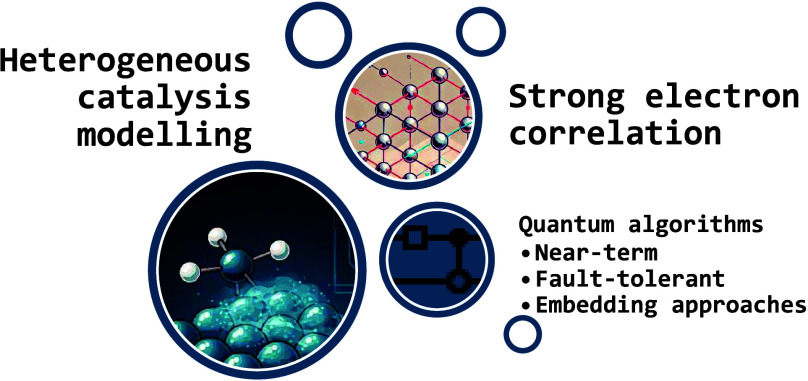

Heterogeneous catalysis plays a critical role in many industrial processes, including the production of fuels, chemicals, and pharmaceuticals, and research to improve current catalytic processes is important to make the chemical industry more sustainable. Despite its importance, the challenge of identifying optimal catalysts with the required activity and selectivity persists, demanding a detailed understanding of the complex interactions between catalysts and reactants at various length and time scales. Density functional theory (DFT) has been the workhorse in modeling heterogeneous catalysis for more than three decades. While DFT has been instrumental, this review explores the application of quantum computing algorithms in modeling heterogeneous catalysis, which could bring a paradigm shift in our approach to understanding catalytic interfaces. Bridging academic and industrial perspectives by focusing on emerging materials, such as multicomponent alloys, single-atom catalysts, and magnetic catalysts, we delve into the limitations of DFT in capturing strong correlation effects and spin-related phenomena. The review also presents important algorithms and their applications relevant to heterogeneous catalysis modeling to showcase advancements in the field. Additionally, the review explores embedding strategies where quantum computing algorithms handle strongly correlated regions, while traditional quantum chemistry algorithms address the remainder, thereby offering a promising approach for large-scale heterogeneous catalysis modeling. Looking forward, ongoing investments by academia and industry reflect a growing enthusiasm for quantum computing’s potential in heterogeneous catalysis research. The review concludes by envisioning a future where quantum computing algorithms seamlessly integrate into research workflows, propelling us into a new era of computational chemistry and thereby reshaping the landscape of modeling heterogeneous catalysis.

## 1 Introduction

Heterogeneous catalysis, a dynamic field at the intersection of chemistry, materials science, and engineering, plays a pivotal role in enabling efficient and sustainable chemical transformations.^1^ Heterogeneous catalysis involves the utilization of solid catalysts to accelerate chemical reactions by providing an alternative reaction pathway with lower activation energy. Unlike homogeneous catalysis, where the catalyst and reactants exist in the same phase, heterogeneous catalysis leverages the unique properties and high surface area of solid catalysts to facilitate reactions between gas, liquid, or solid reactants.^2^ This ability to operate in diverse reaction conditions and effectively couple different phases makes heterogeneous catalysis an indispensable tool in a wide range of industries, including energy production,^3^ environmental remediation,^4^ and chemical synthesis.^5^

A key aspect driving the evolution of heterogeneous catalysis is its central role in sustainable chemistry.^3^ The pursuit of greener and more sustainable chemical processes necessitates catalysts that can enable highly efficient and selective reactions while minimizing energy consumption and waste production. Heterogeneous catalysis provides a promising avenue to achieve these goals by enabling the design of catalysts with tailored properties, such as active site engineering,^6^ incorporation of nanoparticles,^7^ and optimization of surface morphology.^8^

The surfaces of heterogeneous catalysts act as active sites, where reactant molecules undergo adsorption, diffusion, and subsequent reactions.^6^ These surfaces offer interactions of different types, such as Pauli repulsion, physisorption (weak bonding), chemisorption (strong bonding), which dictate the overall catalytic activity and selectivity. The complexity of these processes, coupled with the dynamic nature of catalysts, necessitates a deep understanding of the underlying principles governing surface chemistry and catalytic mechanisms.^9^ In recent years, the field of heterogeneous catalysis has witnessed tremendous advancements driven by both experimental and theoretical investigations. State-of-the-art characterization techniques, such as surface-sensitive spectroscopy and microscopy, have unraveled the intricate details of catalyst structure and composition, shedding light on the correlation between surface properties and catalytic performance.^10−18^ Moreover, computational methods, ranging from various electronic structure methods for transition state characterization, ab initio molecular dynamics and advanced sampling, machine learning algorithms, multiscale modeling, etc., have emerged as powerful tools for elucidating reaction pathways, catalyst design, and high-throughput screening of new materials.^19−31^

Density functional theory (DFT) has emerged as the workhorse for modeling heterogeneous catalysis, providing a powerful and efficient framework for understanding catalytic processes at the atomic and molecular level.^19,21^ Since DFT is an established method, it has not been discussed in this review. Interested readers are referred to the following articles and books for more information.^21,32−34^ DFT offers a practical approach to calculate electronic structure and predict reaction energetics, allowing researchers to explore the activity, selectivity, and stability of catalysts. It has been successfully employed to study a wide range of catalytic phenomena, including adsorption, surface reactions, reaction dynamics and catalytic cycles.^21^ However, despite its wide applicability, DFT has inherent limitations,^35^ most importantly its reliance on approximate exchange-correlation functionals, which can introduce errors in the description of nonlocal and strong correlation effects. In heterogeneous catalysis, where open-shell systems like transition metals and delocalization of electrons are common, strong correlation effects are often present, necessitating methods that go beyond the single-reference/single-configuration approach.^35−38^ Multireference/multiconfiguration methods, such as complete active space self-consistent field (CASSCF)^39,40^ combined with multireference perturbation theory (MRPT), for instance complete active space second-order perturbation theory CASPT2^41−43^ or N-electron valence state second-order perturbation theory (NEVPT2)^44−46^ are essential to capture static and dynamic correlation and accurately describe the electronic structure and energetics of complex catalytic systems. These advanced methods are crucial for understanding the details of catalytic reaction mechanisms and designing more efficient and selective catalysts for sustainable chemical processes.

In CASSCF calculations, the computational complexity is directly influenced by the size of the active space. As this active space expands to encompass more orbitals and electrons, the number of potential configurations increases factorially due to the combinatorial nature of this way of treating electron correlation.^47^ This factorial scaling renders these calculations computationally demanding, particularly for larger and more complex molecular systems. For large active spaces, the memory requirements become prohibitively large, making it impractical to store the full wave function explicitly. This limitation restricts the application of CAS methods to relatively small active spaces and limits the size and complexity of systems that can be studied accurately. The largest CAS calculations performed so far involve active spaces of up to a 44 orbitals and 44 electrons.^48−50^ To overcome the limitations of explicit wave function storage, various methods have been developed to exploit sparsity and exploit tensor network representations, such as density matrix renormalization group (DMRG),^51^ matrix product states (MPS)^52^ or tree tensor networks (TTN),^53^ to represent the wave function more compactly. These approaches offer a way to approximate and compress the wave function information, enabling the treatment of larger active spaces than would be possible with traditional storage techniques. However, even with these advancements, the scalability of CAS calculations on a classical computer is still a formidable challenge.

The emerging field of quantum computing offers an alternative and compelling avenue for pushing the boundaries of heterogeneous catalysis modeling.^54−57^ Quantum computers derive their power from the ability of quantum algorithms to represent and manipulate exponentially large wave functions, enabling more accurate simulations of strongly correlated molecules, their reaction kinetics, and high-throughput screening of catalytic materials. Unlike classical computers, quantum computers can efficiently compute energies of highly complex model wave functions using methods such as quantum phase estimation, which allows for precise energy calculations and other critical properties, such as density matrices. This capability is crucial for tackling challenges such as strong correlation effects, nonadiabatic processes, and large-scale systems that are computationally prohibitive for classical methods.^55,57^ As future error-corrected quantum processing units (QPUs) become available, they will further enhance the ability to treat larger active spaces and handle strong electron correlations. (N qubits have the capacity to represent 2^*N*^ complex numbers, equivalent to requiring 2^*N*+7^ bits for representation in double precision on classical computers.) This could lead to more accurate and scalable modeling of complex systems, enabling insights into elusive reaction intermediates and catalytic mechanisms, and accelerating the design of novel catalysts with improved performance and selectivity.^58,59^ Having said that, the near-term quantum computers and their associated algorithms are crucial as they serve as initial steps toward fault-tolerant quantum computing. Although these devices do not incorporate full error correction, they enable researchers to test, refine, and develop quantum algorithms, such as the variational quantum eigensolver (VQE)^60^ highlighted in this article. Understanding their capabilities is essential, as it offers valuable hands-on experience with quantum hardware and helps bridge the gap between current technology and future error-corrected QPUs.

Furthermore, quantum computers can generate wave functions using algorithms like the variational quantum eigensolver (VQE).^54^ However, their accuracy is limited by factors such as hardware coherence, ansatz expressivity, quantum noise and barren plateaus.^61^ Scalability also presents a challenge, as current devices struggle with large or highly entangled quantum states. A recent study indicates that while heuristic quantum state preparation is efficient for some problems, no clear exponential advantage has been demonstrated in chemical space.^62^ Perfect ground state preparation is often assumed, but it is rarely achievable. A recent approach addresses this by improving the initial state overlap with the true ground state, accelerating matrix product state (MPS) preparation, and employing efficient filtering methods.^63^ This approach enhances the likelihood of preparing high-overlap states in complex chemical systems, but reliable, scalable wave function generation remains a challenge. Quantum computing presents both opportunities and challenges. Despite these, leveraging quantum algorithms and simulations could provide valuable insights into elusive reaction intermediates, unravel complex catalytic mechanisms, and enable the design of novel catalysts with improved performance and selectivity in heterogeneous catalysis systems with strong correlation.

In heterogeneous catalysis modeling, embedding methods bridge the gap between accurate but expensive electronic structure calculations and computationally feasible simulations of entire catalyst systems. These methods treat a smaller, active region of the catalyst (e.g., the metal surface and adsorbates) with a high level of theory, while embedding it within a simpler representation of the surrounding bulk material.^64^ This allows for capturing crucial chemical interactions at the active site while maintaining computational efficiency. However, a key limitation lies in defining the appropriate boundary between the embedded region and the surrounding environment, as it can affect the accuracy of the results. Embedding methods pave the way for incorporating quantum mechanics into simulations of complex catalytic systems.^65,66^ These approaches are crucial for accurately modeling regions with strong (static) correlation while allowing the rest of the system to be handled by different methods. These techniques distinguish between types of electronic correlation (static versus dynamic), enabling the use of the most suitable solver for each region. Embedding methods exemplify a hybrid quantum–classical framework, leveraging the strengths of quantum processing units (QPUs) for strong correlations and classical CPUs for dynamic ones. This highlights that quantum and classical computers are not mutually exclusive; instead, they are alternative and complementary tools. By employing this hybrid strategy, most computations are performed on classical systems, reserving quantum processing for aspects that classical algorithms cannot effectively manage. As the field progresses toward quantum-centric computing,^67,68^ these embedded regions themselves could be tackled with even higher accuracy using quantum algorithms, leading to a new level of detail in heterogeneous catalysis modeling.

Another area where quantum computing may have an impact is in understanding kinetics and quantifying uncertainties arising from incomplete knowledge of reaction constants and mechanisms.^69−72^ This is essential for optimizing reaction conditions by enabling the accurate prediction of reaction rates and the identification of optimal parameters for desired outcomes. These problems can be cast as linear systems of equations.^73−75^ Therefore, recent advancements, particularly the application of the Harrow–Hassidim–Lloyd (HHL) algorithm^76^ and its variational counterpart, the Variational Quantum Linear Solver (VQLS),^77^ offer promising prospects for an enhanced understanding and description of microkinetic models, which facilitate the elucidation of reaction mechanisms and the prediction of reaction rates by treating them as linear systems of equations. Uncertainty quantification is crucial in this context as it allows for the assessment and reduction of uncertainties in model predictions, leading to more reliable and efficient optimization strategies.

In the rapidly evolving landscape of quantum computing, extensive reviews on its applications to various domains of quantum chemistry have emerged, including energy applications,^78^ biochemistry,^79,80^ drug development,^81^ and fusion.^82^ However, to our knowledge, despite the growing interest in harnessing quantum computing for catalysis,^83^ a notable gap exists in the literature regarding dedicated reviews on the topic of quantum computing for heterogeneous catalysis or chemical reactions at surfaces, in general. Therefore, this review article aims to fill this gap by providing an in-depth exploration of the emerging field of quantum computing for quantum chemistry applications in the context of heterogeneous catalysis. By surveying the latest advancements in quantum algorithms and applications, methodologies, and challenges, this review seeks to provide a comprehensive overview and critical analysis of the potential and challenges associated with utilizing quantum computing for advancing the field of heterogeneous catalysis.

## 2 Atomistic Modeling of Heterogeneous Catalysis: Status and Challenges

In heterogeneous catalysis, the presence of a catalyst in a distinct phase, typically solid, accelerates a reaction involving reactants in a different phase, such as liquid or gas. Among the various forms of heterogeneous catalysts, dispersed metal nanoparticles on oxide supports are widely prevalent.^7^ However, accurately modeling the complex interface comprising the oxide support, metal nanoparticle, and reactants under operational conditions of temperature and pressure presents significant computational challenges.^18,28^ To gain insights into the underlying mechanisms of these reactions, researchers often study simplified models, such as clean two-dimensional surfaces representing the most exposed facet of the metal nanoparticle. While these models capture the rate-determining steps, the effects of reactant concentration and pressure are typically excluded, model limitations referred to as the “materials gap” and “pressure gap,” respectively.^1^ To incorporate the influence of reaction temperature, various approaches like the sudden model^84^ and ab initio molecular dynamics^85^ are employed. To be able to include the thermodynamic effects *viz*., pressure and temperature and to obtain thermodynamic quantities like Gibbs free energies *ab initio thermodynamics* (AITD) can be used.^86,87^ Overcoming these gaps remains a notable challenge in the field.

**Figure 1:**
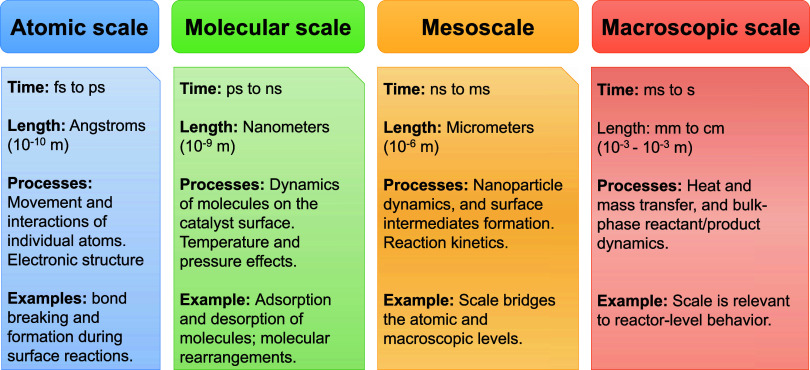
Time and length scales in heterogeneous catalysis illustrating processes across atomic, molecular, mesoscopic, and macroscopic scales. The figure depicts an increase in both time and length scales from left to right, which captures the intricate dynamics of catalytic reactions at progressively longer time and larger length scales.

In heterogeneous catalysis, understanding the catalytic cycle and predicting catalytic activity involves considering multiple levels viz., atomic scale, molecular scale, mesoscale and macroscopic scale, of modeling and analysis (Figure 1). Electronic structure calculations play a crucial role in providing detailed and predictive information about individual elementary processes within the catalytic cycle at the *atomic scale*, such as adsorption and reaction energies and energy barriers associated with chemical reactions. At the *molecular scale*, one examines the dynamics of molecules on the catalyst surface, accounting for temperature effects, generally, within the harmonic approximation. Occasionally, effects beyond the harmonic approximation and pressure effects are also incorporated at this modeling stage. Building upon this, in the *mesoscale* first-principles microkinetic models utilize the electronic structure information to assess the intricate interplay between all elementary processes, enabling the determination of the intrinsic catalytic activity. In real catalysts, an intermediate step is needed to appropriately coarse-grain the microstructure of the catalyst, ensuring the effective integration of catalytic activity with transport models. Finally, to fully understand the overall macroscopic flow of heat and mass in real catalysts, it becomes necessary to integrate the intrinsic catalytic activity into transport models. In this *macroscopic scale*, it is important to consider how catalytic activity interfaces with larger-scale processes governing heat and mass transfer.

### 2.1 Industrial Relevance of Computational Modeling in Heterogeneous Catalysis

Heterogeneous catalysis is vital for sustainable energy applications,^3^ but material selection has relied on intuition and serendipity for a long time. Nanoscale systems like oxide supports, alloys, and dopants are too complicated to be represented in full atomistic detail in a model, but representative smaller models that can be treated computationally aid in understanding reaction mechanisms and provide descriptors to aid in rational catalyst design.^21,88^ In this regard, DFT-based computational modeling of heterogeneous catalysis has established itself also as crucial in industrial research^89,90^ with the atomistic simulations offering insights into the parameters relevant for catalyst design such as electronic properties and reaction energies. Descriptors like the *d*-band center, volcano plots, Sabatier principle, and BEP relationships have proven successful in catalyst design.^19^ Various surface and energy descriptors contribute to predicting catalytic performance, supporting catalyst optimization.^91−94^ A recent study outlines a general approach to identify the best catalyst by analyzing a data set of reactions under kinetic control, calculating normalized key performance indicators (KPIs), and using KPI plots to demonstrate the optimal catalyst selection in two case studies: acetylene hydrochlorination for vinyl chloride production and the selective oxidation of methane to methanol.^95^

While density functional theory (DFT) provides valuable data, integrating first-principles rate constants into higher-scale models raises important questions regarding error propagation.^96^ Computational studies have proven beneficial in various aspects of heterogeneous catalysis research, and the integration of ab initio molecular dynamics (AIMD) with high-level theories for complex catalytic site models has become increasingly practical in recent times.^90,97^ Bridging the gap between different scales in heterogeneous catalysis modeling remains a challenge,^9^ requiring efforts to integrate complexity levels for a comprehensive understanding. The emerging synergy between computational modeling and machine learning^31^ holds promise for determining surface properties and chemical reactivity, opening avenues for future advancements in catalysis research and the efficient development of catalysts with industrial applications.

As we seek more efficient and selective catalysts for various chemical reactions, including multicomponent alloys,^98^ single-atom catalysts,^99^ magnetic catalysts,^100,101^ etc., addressing the challenges posed by strong correlation effects^102^ and spin-related phenomena^103^ becomes imperative. While DFT-based computational studies have been valuable in addressing numerous aspects of heterogeneous catalysis, our focus is on two important research areas with significant future potential: 1) studying strong correlation effects in heterogeneous catalysis and 2) investigating spin effects in heterogeneous catalysis. Strong correlation effects, arising from electron–electron interactions in transition metal complexes, bimetal and alloy catalysts, demand advanced computational approaches beyond standard DFT methods^35^ as the mean-field approach taken by Kohn–Sham DFT has limitations in predicting reaction pathways and accurate energetics for systems with such complicated, open-shell, electronic structures. Additionally, effects related to electron spin in heterogeneous catalysis, particularly in magnetic catalysts or systems with pronounced spin-polarized states, further complicate computational modeling.^101,104,105^ The interaction between electron spins, which determines the catalyst’s magnetic properties, introduces complexities challenging the predictive capabilities of DFT. These challenges hinder DFT’s ability to provide precise insights into spin-dependent catalytic processes, limiting the reliability of calculated reaction mechanisms and electronic structures. Overcoming these challenges is crucial for advancing computational methodologies in heterogeneous catalysis.

Furthermore, microkinetic modeling in heterogeneous catalysis is important yet complex due to the interplay of multiple reactions, intermediates, and pathways involving adsorption, dissociation, desorption, and surface diffusion on catalyst surfaces.^106^ Reaction kinetics also vary with temperature, pressure, and concentration, challenging model accuracy. Simplified models often use scaling relations based on electronic structure descriptors, which may not fully capture real-world behavior.^107^ Limited experimental data further complicates fitting these models, requiring benchmarks against experimental targets like turnover frequency (TOF), an important quantity that defines the activity of the catalyst. However, uncertainty arises at various levels in multiscale modeling, sepecially microkinetic modeling, potentially compounding and impacting prediction accuracy. Uncertainty quantification in microscale modeling plays a crucial role in accurately modeling heterogeneous catalysis under industrial conditions.^108^ Therefore, it is important account for the various uncertainties at multiple levels in the modeling process, ensuring that predictions align more closely with real-world catalytic behavior.

### 2.2 Strong Correlation in Heterogeneous Catalysis

**Figure 2:**
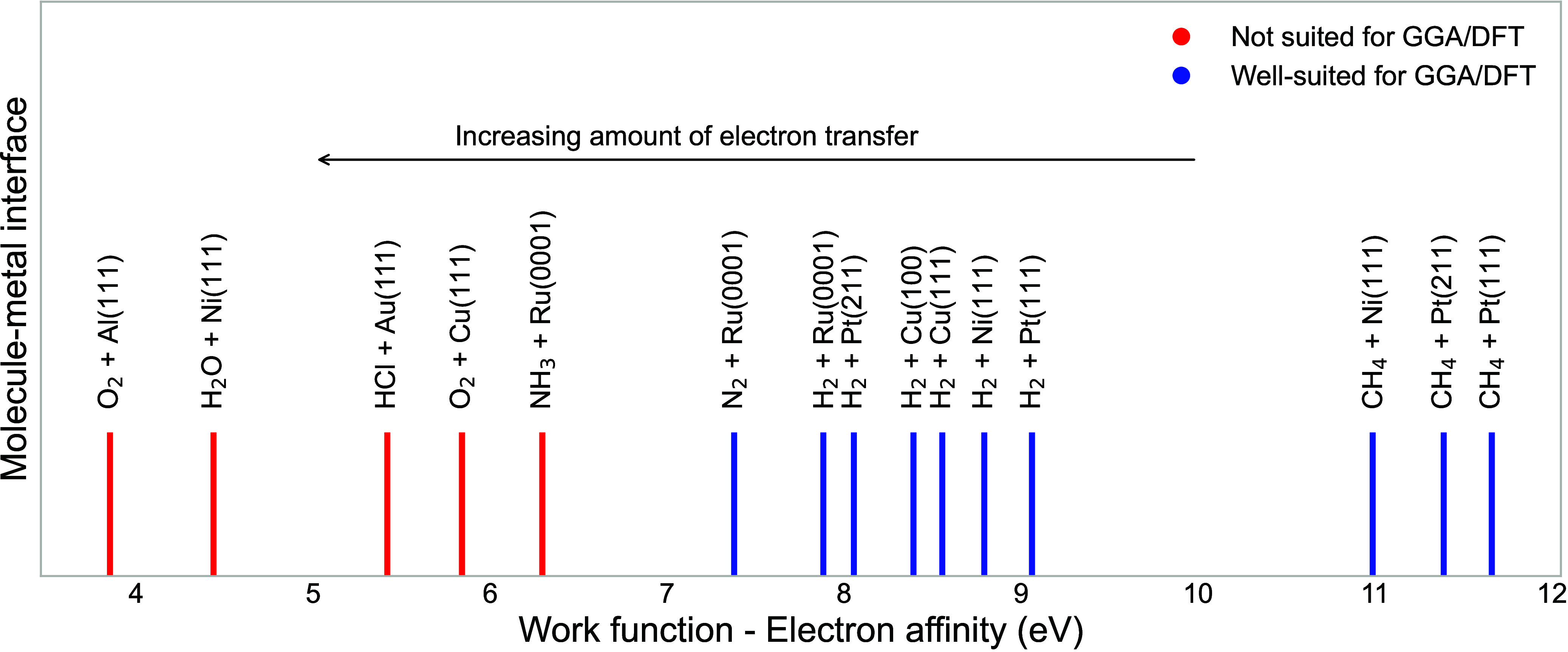
Relationship between the difference in work function of the metal surface (*W*) and the electron affinity (*E*_ea_) of the molecule (in electronvolt, eV). This correlation highlights the influence on the accuracy of GGA exchange-based density functionals in predicting barrier heights for direct electron transfer in the studied systems. The color-coded scheme (red and green) indicates the efficacy of density functionals (DF) based on GGA exchange with red (large electron transfer) denoting scenarios not suited for GGA/DFT and green (small electron transfer) representing successful candidate GGA/DFT for describing energetics and dissociative chemisorption dynamics in various molecule–metal surface reactions. Replotted data from ref (115) under Creative Commons CC-BY-NC-ND license 4.0.

At the atomic scale, wave function-based methods with atom-centered basis sets are effective in describing gas-phase reactions. Conversely, for bulk and surface systems, the prevalent approach is periodic DFT utilizing plane wave basis sets. In the context of heterogeneous catalytic reactions, especially those involving metal atoms and two-dimensional surfaces with periodicity, this combination has therefore become the predominant modeling approach. Within the realm of DFT, various exchange-correlation functional approximations have been developed, ranging from local density approximation (LDA) to generalized gradient approximation (GGA), meta-GGA, and hybrid functionals.^33^ Dispersion interactions can be included as well, either via the economical methods developed by Grimme,^109−111^ Tkatchenko and co-workers,^112^ many-body dispersions^113^ or more explicitly by performing random phase approximation (RPA) calculations.^114^ GGA functionals are preferred for their trade-off between cost and accuracy for large scale modeling of heterogeneous catalysis reactions. While DFT with standard GGA functionals proves successful in many cases, it encounters challenges when charge or electron transfer is involved between the molecule and the metal surface as shown in Figure 2.^115^

In numerous molecule-metal surface systems, GGA exchange density functionals (DFs) face limitations as they tend to be overly reactive, surpassing experimental reactivity.^115^ A critical determinant for the applicability of GGA functionals in capturing the barrier to dissociative chemisorption with chemical accuracy lies in a property which characterizes molecule-metal surface systems. This property, the difference (*W* – *E*_ea_) between the metal’s work function (*W*) and the molecule’s electron affinity (*E*_ea_), is indicative of the system’s tendency for charge transfer.^116,117^ The work function (*W*) of a material represents the minimum energy required to remove an electron from its surface, measured in electron volts (eV). A lower work function indicates easier electron emission. Electron affinity, also measured in eV, reflects the energy change when adding an electron to a neutral atom or molecule to form a negative ion. A higher electron affinity suggests a greater tendency to accept an additional electron. The comparison of difference between work function and electron affinity empirically determines the amount of charge transfer (Figure 2). In molecule-metal systems, when the *W* – *E*_ea_ exceeds 7 eV, generalized gradient approximation (GGA) in density functional theory effectively describes the energetics and dynamics of the dissociative chemisorption processes. However, if it is below 7 eV, GGA-DFT struggles to produce reliable energetics and dissociative sticking probabilities, often attributed to significant charge/electron transfer. While nonadiabatic effects are ruled out^118^ as the primary explanation for this failure of the GGA, ascending the DFT ladder to higher functionals, such as meta-GGA or hybrid DFs, proves effective in addressing errors in barriers for gas-surface reactions. The success of meta-GGA DFs, particularly in semiquantitative agreement with experimental results,^115^ suggests the viability of an electronically adiabatic approach, emphasizing the importance of the electronic structure treatment. The study of strong correlation effects in molecule-metal interfaces plays a crucial role in advancing our understanding of heterogeneous catalysis.^35,36,102^ In this context, several examples of strong correlation phenomena have been observed, shedding light on the complex nature of chemical reactions at the atomic level. One of the classic examples is the H_2_ dissociation on Li_4_ and Li_6_ clusters.^36,119^ Previous research, has highlighted the limitations of conventional computational methods in accurately describing H_2_ dissociation on Si(100) surfaces due to the presence of strong correlation effects.^120,121^

An intriguing example that highlights the limitations of standard DFT is the O_2_ dissociation on metal surfaces.^122^ In this reaction, the spin-flipping of O_2_ on numerous metal surfaces poses a challenge that previous studies failed to address adequately. Moreover, the ability to confirm or refute the occurrence of O_2_ spin-flip when the molecule approaches the metal surface remains elusive. It is well-known that molecular O_2_ exists in a ground state as a triplet (two electrons unpaired), which is unreactive, while the excited singlet (no unpaired electrons) state readily engages in reactivity. The mechanism underlying the conversion between the triplet and singlet states during O_2_ dissociation on metal surfaces still remains a mystery. Behler et al.^123−125^ found that the absence of a barrier in DFT for O_2_/Al(111) results from DFT predicting charge transfer at large O_2_-surface distances. They addressed this by using locally constrained DFT, enforcing the O_2_ molecule to stay in its triplet state, revealing a barrier. The success of this method prompted exploration into the role of spin selection rules in gas-surface interactions. Furthermore, to understand the dynamics on potential energy surfaces corresponding to different spin states, nonadiabatic models were also developed.^126−128^ Libisch et al. proposed that the barrier for O_2_ dissociation on Al(111) is not governed by spin conservation rules but arises when charge transfer is adequately treated,^118^ demonstrated using embedded correlated wave function methods.^129^ Their approach, employing DFT for surface energy and correlated wave function theory for O_2_ interaction, produced two-dimensional potential energy surfaces (PESs) consistent with experimental observations. However, the definition of an overall electronic state of the entire molecule–surface reaction remains unclear within periodic DFT. Despite indicating that spin is likely not the primary reason, and charge transfer plays a crucial role in the theory-experiment discrepancy, concrete evidence disproving the role of the spin of the incoming O_2_ molecule and the magnetic moments of the Al(111) surface is lacking. It is essential to determine whether the model wave function is strongly multiconfigurational as this gives a good indication of strong correlation effects. At present, it is worthwhile to pursue research on both the role of charge-transfer and of spin flipping until one of the hypotheses is conclusively disproved.

### 2.3 Role of Spin in Heterogeneous Catalysis

Spin, an inherent property of elementary particles, characterizes their intrinsic angular momentum and entails magnetic properties for particles with nonzero spin. In quantum computing, spin is a fundamental property, serving as the foundation for qubits, analogous to classical bits, and facilitating quantum information processing and algorithms. Meanwhile, in chemistry, spin profoundly influences reaction pathways and catalytic processes. Extensive research has explored the role of spin in homogeneous, inorganic, and biochemistry catalysis, leading to valuable insights.^130,131^ Two of the most widely cited examples in quantum computing applications for drug discovery and catalytic materials discovery are cytochrome P450 (CYP)^132^ and the FeMo-cofactor (FeMo-co) of the nitrogenase enzyme,^83^ respectively. In both cases, changes in spin states during substrate binding play a crucial role in facilitating the reaction. In drug discovery, the family of CYP enzymes, which contain heme as a cofactor exhibit changes in spin state while binding oxygen (O_2_),^132,133^ where heme, is a ring-shaped iron-containing molecular component of hemoglobin, which is necessary to bind oxygen in the bloodstream. Specifically, the iron (Fe) center within heme in CYP exists in multiple oxidation and spin states depending on substrate binding and oxygen coordination.^133^ In the resting state (stable or inactive state before substrate binding or catalysis begins), the Fe typically exists in a low-spin Fe^III^ configuration, which transitions to a high-spin state upon substrate binding, facilitating the coordination and activation of molecular oxygen.^134,135^ This transition is crucial for enabling the enzyme’s function, as the high-spin state of the iron makes the binding of substrates more energetically favorable. This spin transition prepares the iron for electron transfer, enabling a series of redox steps essential for catalysis. During the reaction cycle, the heme iron also toggles between Fe^II^ and Fe^III^ oxidation states, enhancing the enzyme’s capacity to activate the bound oxygen molecule and introduce an oxygen atom into the substrate. The connection between spin state and catalytic activity in cytochrome P450 thus provides one clear example of how spin plays a role in catalytic activity in enzymes with metal center in the drug discovery setting.

The other example, is obviously the most celebrated chemical system in quantum computing setting is the FeMo-cofactor of nitrogenase catalyzes the conversion of nitrogen (N_2_) into ammonia (NH_3_) under ambient conditions, a process essential for biological nitrogen fixation.^83,136^ In contrast to CYP, which has one metal center (Fe), FeMo-co has multiple metal centers (Fe and Mo atoms), which makes the spin dynamics more interesting and more complicated to model. These iron atoms in FeMo-co exhibits a complex electronic structure, where multiple oxidation states, notably Fe^II^, Fe^III^, and Mo^IV^, lead to a large number of unpaired electrons, resulting an open-shell system containing unpaired electrons, resulting in a nonzero total spin and allowing for various spin states.^137^ The open-shell nature of FeMo-co gives rise to multiple spin states, with a stable ground-state net spin of *S* = 3/2 achieved through specific spin alignments, such as a 4-up/3-down arrangement among Fe atoms.^138^ This dynamic spin and charge state flexibility enables efficient electron and proton transfer, critical for nitrogen reduction.^83^ Oxidation state and protonation shifts also alter magnetic interactions, notably affecting Mo’s spin state and its antiferromagnetic couplings with Fe, thereby influencing reactivity.^139^ These spin state modulations are believed to be essential for the enzyme’s efficiency, underlining spin’s role in reaction energetics—a factor now being rigorously explored in quantum computing.^137^ However, in line with the focus of this review on heterogeneous catalysis, the discussion on spin will specifically center around its significance in chemical reactions occurring at surfaces and nanoparticles.

The role of spin in chemical reactions at surfaces and its significance in heterogeneous catalysis have garnered considerable attention in recent years. Early proposals on spin catalysis^140,141^ laid the foundation for exploring the influence of spin in catalytic processes. The original *d*-band center model, proposed by Hammer and Norskov,^142^ provided insights into the reactivity of metals, but it was later revised to incorporate the effects of spin polarization.^143^ The importance of spin is evident in reactions involving OCCO and CO^2^ intermediates^144^ in CO^2^ reduction reaction (CO^2^RR). In catalytic CO^2^ reduction, intermediates like OCCO and CO^2^ often adopt high-energy configurations upon chemisorption to metal surfaces, where spin uncoupling plays a crucial role in their reactivity. When CO^2^ or OCCO binds to a metal catalyst, such as platinum or copper, the interaction can lead to spin uncoupling—where the initially paired electron spins within these molecules are partially unpaired as they interact with the metal’s surface electrons. This spin uncoupling facilitates electron transfer between the adsorbed molecule and the metal, enabling activation of otherwise stable bonds in the CO^2^ molecule. For example, the formation of the OCCO intermediate involves two CO units coupling on the metal surface, with spin effects playing a role in stabilizing this high-energy configuration. The spin state of these intermediates impacts bond-breaking and bond-formation steps, such as CO bond cleavage in CO^2^, which are essential for efficient catalytic reduction pathways. Spin uncoupling in these adsorbed intermediates is therefore integral to lowering reaction barriers and improving catalytic turnover, providing valuable insights into the design of spin-tuned catalysts for sustainable CO^2^ conversion.

Furthermore, spin was found to play a crucial role in ammonia synthesis^145^ as well. Recently, Cao and Norskov conducted a systematic study that further underscored the significance of spin in chemical catalysis.^105^ They showed that inclusion of spin polarization decreased chemisorption strengths. A similar conclusion was also reached in earlier studies for N_2_ adsorption on various transition metal surfaces^143^ and for O adsorption on Pt_3_-transition metal alloys.^103^ Spin effects are also prominent in the context of molecular adsorption on Pt_3_M alloy catalysts, where the spin polarization of metal surfaces can significantly influence the binding strength and reactivity of adsorbates. In Pt_3_M alloys—where M represents transition metals such as Fe, Co, or Ni—the introduction of a second metal alters the electronic structure of platinum, including its spin polarization. A detailed discussion on the spin effects in Pt_3_M alloy catalysts is provided in Section 2.3.2 The lowering of chemisorption energies was attributed to filling of antibonding states of the predominant up-spin in the spin-polarized density of states. Experimental evidence supporting the predictions of spin effects in O_2_ interactions with pristine and defective graphene/graphite surfaces has only recently emerged, reinforcing the relevance and importance of spin effects in surface chemistry and heterogeneous catalysis.^146^ Spin effects have also been observed in other catalytic systems, such as phthalocyanines,^147^ electrocatalysis,^148^ and photocatalysis reactions.^149^

Given the prominence of DFT in heterogeneous catalysis, it is important to note that, in spin-DFT for open-shell systems, the spin density can be qualitatively inaccurate, especially in low-spin states, often requiring a broken-symmetry description.^130^ In nonrelativistic scenarios, setting up Kohn–Sham-DFT involves choosing between spin-restricted and spin-unrestricted formulations. While the former ensures the wave function of the noninteracting reference system is always an eigenfunction of *S*^2^, its spin density deviates from the correct one. Conversely, spin-unrestricted KS-DFT provides the correct spin density for the noninteracting reference system but precludes it from being an eigenfunction of *S*^2^. Developing exchange–correlation functional approximations is possible for either formalism, but the choice of restricted vs unrestricted imposes different constraints such as on the fractional occupancy of spin orbitals,^150^

We will highlight two practical examples where spin plays a crucial role in catalytic activity: single atom catalysts (SACs) and Pt_3_M catalysts, both involved in the oxygen reduction reaction (ORR). These examples were chosen because of the complex nature of the interacting species, involving pronounced strong correlation effects and the unconventional triplet ground state of O_2_. The subsequent sections will offer a detailed exploration of these examples.

#### 2.3.1 Single-Atom Catalysts (SACs)

Single-atom catalysts (SACs) are materials where individual isolated metal atoms act as catalytically active sites.^151^ Unlike traditional heterogeneous catalysts, where metal particles or clusters contribute to the catalytic activity, SACs consist of individual metal atoms dispersed on a support material. SACs have gained attention in catalysis research due to their potential for improving efficiency, selectivity, and atom utilization in various chemical reactions, offering advantages in terms of diversity of applications^152,153^ and economic and environmental sustainability.^154^

The unique electronic and geometric properties of these isolated metal atoms can lead to enhanced catalytic performance, as they expose a maximum number of active sites and often exhibit distinct reactivity.^155^ The computational chemistry community is particularly intrigued by SACs due to their distinct ability to catalyze important reactions using a single active center. Despite the apparent simplicity of SACs, modeling their activity poses significant complexity and challenges for theorists, highlighting the difficulty in constructing realistic models that faithfully represent the intricacies of the active site. Numerous computational studies were conducted on this topic to understand the origin of reactivity and the electronic effects governing catalytic activity and selectivity in SACs.^156−158^ In SACs, the metal atom is typically in a low coordination environment, and the unsaturated *d* shell then gives rise to strong local electron correlation. These correlation effects are hard to describe with traditional DFT methods,^158^ making SAC modeling quite sensitive to the choice of functional approximation.^158^ To illustrate this, we discuss below the spin-related aspects of SACs by considering a few specific examples.

The crucial role of spin in SACs for the ORR was studied with DFT using octahedral transition metal complexes and Fe-based SACs in N-doped graphene.^159^ This study highlights the sensitivity of spin state ordering and reactivity predictions to the chosen functional approximation. An increased Hartree–Fock (HF) exchange fraction was found to enhance accuracy, a trend transferable across various ligand environments. To advance the understanding of SACs, a multilevel approach is likely needed: addressing challenges related to spin as the Fe-center and graphene itself with high-level methods, while employing relatively affordable DFT with range-separated hybrids for larger periodic simulations. Concurrently, research on single metal atoms supported for catalysis has shown promising progress, particularly in N-coordinated Fe single atoms distributed over axial carbon micropores (d-FeN_4_).^99^ These SACs exhibit notably higher intrinsic activity in ORR compared to other catalysts. The unique spin characteristics of d-FeN_4_ contribute to faster kinetics during ORR, providing a valuable starting point for advanced energy catalysis. For understanding the operation of SACs containing 3*d*-metal single sites, their magnetic nature necessitates in-depth exploration of the oxidation state and spin state of the active site, as well as the investigation of spin polarization, indicated by its magnetic moment.^160^ DFT-calculated partial density of states (PDOS) and Wannier function analyses provide some first descriptors to this end. Using first-principles calculations, the two-dimensional ferromagnetic metal–organic framework Mn_2_C_18_H_12_ was identified as a highly efficient SAC for spin-triplet O_2_ activation and CO oxidation.^161^ The mechanism proposed, known as ’concerted charge-spin catalysis’ involved a synergistic process of charge transfer from the hosting Mn atom and spin selection facilitated by its nearest neighboring Mn atoms during O_2_ activation. This synergistic mechanism was proposed to exhibit broad applicability in O_2_ adsorption on magnetic frameworks X_2_C_18_H_12_ (X = Mn, Fe, Co, and Ni), showing a linear scaling dependence between chemical activity and spin excitation energy. Computational study on the catalytic activity of Fe single-atoms supported on C_2_N (C_2_N–Fe) in the ORR reaction uncovered a direct relationship between changes in electronic spin moments of Fe and O_2_, induced by molecular-catalyst adsorption, and the amount of electron transfer from Fe to O_2_.^162^ This electron transfer was found to enhance the ORR catalytic activity of C_2_N–Fe. Due to the observed linear correlation, the electronic spin moment was proposed as a promising catalytic descriptor for Fe-based SACs. Magnetic (spin) effects can also used to explain the weakening of the binding energies of adsorbates on SACs, especially for ORR.^163^ However, when magnetic SACs are involved, the functionals used for simulations affect the predicted relative stability of different spin states and, since the spin state may vary during the reaction process,^157^ potentially also the predicted minimum energy reaction pathway. From these examples it is evident that the electronic structure and spin-related phenomena in SACs demand the utilization of advanced computational methodologies, to check and improve the predictions made by DFT. Specifically, the application of multireference/multiconfiguration methods is likely imperative to reliably model the spin transitions occurring at the active sites, especially for applications in ORR.

#### 2.3.2 Pt_3_M Catalysts

Proton-exchange membrane fuel cells (PEMFC) hold promise for sustainable energy applications, relying on catalytic reactions such as the hydrogen oxidation reaction (HOR) and ORR.^164,165^ While platinum (Pt) has conventionally served as the standard catalyst, its high cost has prompted the search for more economical alternatives, leading to the exploration of Pt_3_M alloys (M = 3*d* transition metals). These catalysts are composed of a combination of Pt and another less expensive transition metal (denoted as M) in a ratio of 3:1. Apart from reducing the platinum content, the choice of the other metal influences significantly the catalytic performance and can thereby be used as a tuning parameter.

Past studies have established the enhanced catalytic activity of Pt_3_Ni and Pt_3_Co alloys for ORR, attributed to the inhibition of PtOH_*ad*_ formation and electronically modified Pt atoms.^166−168^ However, the underlying reasons for the increased activity on Pt_3_Ni and Pt_3_Co alloys remained unclear for a considerable period. The origin for enhanced activity was attributed to the synergy among ligand (or electronic structure) effects, strain (or geometric) effects, and ensemble effects.^169^ Conversely, by comparing spin-polarized and nonspin-polarized calculations,^102−104,170^ the influence of spin and magnetic effects, particularly the role of quantum spin exchange interactions (QESI), was elucidated as being a likely cause for the enhanced reactivity of these strongly correlated Pt_3_M catalysts.^103,104^ QSEIs and ferromagnetic spin-electron interactions play crucial roles in facilitating milder chemisorption and spin-selective electron transport, making magnetic catalysts appealing for various applications.^101^

This section underscores the growing importance of incorporating spin effects into catalysis research, highlighting recent advancements in understanding spin-related phenomena at surfaces. While it is common practice to follow reactions along a single spin-state potential energy surface using spin-polarized DFT, certain reactions exhibit two-state reactivity where spin–orbit coupling becomes crucial. The modified *d*-band center approach,^143^ emphasizing the role of spin in catalysis and explicitly considering two spin channels, has shown promise, particularly in reactions with significant spin involvement. In addition, DFT+U approach also has alleviated some problems related to strong correlation in magnetic catalysis and materials.^101,171,172^ However, as the field progresses, despite the robustness of DFT for catalysis modeling, there is a recognized need to integrate more accurate wave function electronic structure theories, such as multireference/multiconfiguration methods.^35,173^

Nevertheless, given the high computational demands of multireference methodologies, as well as their increased complexity in employing them, such as the crucial active space selection and the more complicated interpretation of results,^47^ these methods are still hard to employ routinely. In addition we note that geometry optimizations with CASPT2 is challenging, so that DFT is often used to generate potential energy surfaces, with the multireference methods used only for single-point energy calculations. However, if the DFT model is qualitatively inaccurate for these types of reactions, the reliability of the potential energy surface can be compromised. This situation calls for improved methodology that offer the possibility to work with large active spaces and cover large fractions of the potential energy surfaces of the different spin states to shed light on the interplay between spin and chemical reactivity when designing and optimizing heterogeneous catalytic systems. Since classical computational methods are, due to the factorial scaling of the configuration space with the size of the CAS space, intrinsically limited,^49,50^ quantum computing algorithms hold promise in treating strongly correlated systems for catalysis research. With the emerging role of spin in catalysis, this incorporation of such advanced computational modeling techniques can aid in optimizing and designing new materials.

## 3 Uncertainty Quantification in Heterogeneous Catalysis

Uncertainty quantification (UQ)^174,175^ is a systematic approach for identifying, quantifying, and managing uncertainties in models or simulations. It provides insights into the reliability of predictions by assessing the effects of variations in input parameters. In heterogeneous catalysis, UQ is crucial for accurately modeling complex reactions across multiple scales—from atomic-level interactions to reactor-scale processes—each introducing distinct uncertainties.^71,108,176^ The multiscale modeling framework (see Figure 1) highlights these challenges, with uncertainties arising at each stage: DFT calculations,^177^ molecular dynamics simulations,^178^ microkinetic modeling,^71^ and reactor-scale analysis.^179^ Turnover frequency (TOF), a key performance metric representing the reaction rate per active site, is especially sensitive to uncertainties in reaction mechanisms, rate constants, and surface coverages, all of which influence TOF predictions and, therefore, the reliability of catalytic assessments.^6,180−182^ While quantifying uncertainties at each scale and understanding their propagation to TOF is crucial, this discussion focuses specifically on uncertainties within the microkinetic modeling stage, where various rate equations and surface coverages come into play.

Classical methods, such as Monte Carlo simulations, are widely used to estimate uncertainties.^183^ However, they are computationally intensive due to large parameter spaces and the “curse of dimensionality,” where the cost of sampling grows exponentially with the number of uncertain parameters.^184^ Deterministic sampling techniques, though more efficient for smaller dimensions, still struggle with exponential scaling in high-dimensional UQ problems, making them impractical for complex microkinetic models. Quantum computing presents a promising alternative for efficient UQ, particularly through the Harrow–Hassidim–Lloyd (HHL) algorithm,^76^ which can solve linear systems exponentially faster than classical methods. However, HHL produces a quantum state representing the solution, and extracting meaningful numerical data from this state requires probabilistic measurements. These measurements can become impractical for large systems due to the high number of measurements required.

To address these limitations posed by classical methods, Walker et al.^73−75^ proposed using the HHL algorithm for UQ in prototypical catalytic systems, including CO oxidation on Pt(111), CO oxidation on Ru(111), and H_2_ oxidation in air. Their work focuses on UQ in microkinetic modeling, using the HHL algorithm. The steps to construct a general microkinetic model in heterogeneous catalysis^185^ are as follows:

1. Identify and define each elementary reaction step involved in the system.
2. Derive rate expressions for each of these elementary reaction steps.
3. Convert these rate expressions into a system of ordinary differential equations (ODEs).
4. Define boundary conditions (e.g., partial pressures), initial values (e.g., initial surface concentrations), and any relevant model parameters (e.g., temperature).
5. Solve the system of ODEs to obtain dynamic behavior over time.
6. Interpret the results using chemical intuition to gain insights into the reaction system.

In this quantum context, the requirement for running the HHL algorithm is to have a linear system of equations. To achieve this, the problem (step 5 from the above list) is reformulated into a set of linear rate equations (one additional step added to the above steps), which are then incorporated into the microkinetic model, with assumptions of steady-state and mass balance applied.^73^ The HHL algorithm is used to calculate the surface coverages of CO and O in this example. For uncertainty quantification, the equilibrium model is perturbed in both positive and negative directions.^74,75^ Each perturbation creates a block diagonal matrix, whose eigenvalues are computed, leading to three sets of surface coverages corresponding to these different perturbations. It is important to note that current applications of microkinetic modeling and uncertainty quantification using the HHL algorithm, demonstrated for prototypical systems, involve a very small number of qubits (3 qubits in these examples) and are run on simulators. To achieve practical speedups under realistic conditions, the algorithm needs to be extended, thoroughly understood, and reimplemented. Variational quantum linear solvers (VQLS),^77^ on the other hand, offer a more NISQ-friendly alternative, using variational methods to iteratively minimize a cost function and solve linear systems. VQLS can yield numerical solutions more directly with fewer measurements, making them potentially better suited for near-term quantum devices. Nevertheless, both HHL and VQLS face scalability challenges for large systems and may require advanced techniques like implicit operators or reduced bitstring spaces to make UQ feasible in complex catalytic models. While these efforts are in the early stages, the goal of discussing microkinetic modeling and uncertainty quantification in this review is to emphasize that aspects of heterogeneous catalysis modeling—beyond electronic structure—could benefit from quantum algorithms.

## 4 Prospects of Quantum Computing

In the previous sections, we argued that DFT, the most commonly used method, struggles to accurately capture strong correlation effects in heterogeneous catalysis. As securing a sustainable future creates a large demand for new catalysts, research on sophisticated materials such as multicomponent alloys is important. For modeling chemical reactions facilitated by these types of catalysts it is imperative to reliably treat strong correlation effects. Quantum computing provides a potential solution by offering an effective means to work with strongly multiconfigurational wave functions—an essential ingredient to better treat the regime of strong correlation. Industries have recognized the potential of quantum computing in heterogeneous catalysis and are actively investing in exploring its use cases, aiming to enhance catalyst design and optimization in heterogeneous catalysis.

Collaborations between quantum companies (companies building quantum computers and/or developing quantum algorithms) and companies seeking use cases have been established as a promising path toward technological advancements. Microsoft Azure Quantum has partnered with notable companies like Johnson Matthey, BASF, Ford, and Toyota-Tsusho Corporation to explore various applications.^186,187^ Johnson Matthey, for instance, focuses on finding improved catalysts for hydrogen fuel cells and seeks alternatives to platinum, including the exploration of alloy catalysts. BASF, a leader in catalysis, collaborates with Microsoft Azure Quantum to advance catalytic processes. Ford and Toyota-Tsusho Corporation engage in partnerships to explore battery materials and technologies.

IBM has established collaborations with renowned companies such as Daimler AG (Mercedes Benz), Exxon Mobil, Boeing, Mitsubishi Chemical, JSR, and the University of Keio.^188−192^ The collaborations aim to tackle diverse challenges. For instance, Daimler AG works with IBM to identify candidates for energy-dense battery technology, particularly focusing on lithium–sulfur (Li–S) batteries.^188^ Exxon Mobil utilizes IBM’s expertise in optimization to address problems related to maritime inventory mapping.^189^ Boeing presents two distinct challenges: the optimization of ply design, a critical aspect of aircraft manufacturing, and the development of advanced corrosion-resistant chemicals for airplane coatings.^190^ Mitsubishi Chemicals and JSR, in collaboration with the University of Keio, delve into organic light-emitting diodes and the crucial Li superoxide rearrangement step in Li–O batteries.^191,192^

Another notable consortium in the quantum technology realm is the Quantum Technology and Application Consortium (QUTAC).^193^ Its founding members include BASF, BMW Group, Boehringer Ingelheim, Bosch, Infineon, Merck, Munich Re, SAP, Siemens, and Volkswagen. QUTAC acts as a platform for collaboration and knowledge exchange among these industry leaders in quantum computing for chemistry and materials. BASF leverages the consortium to pursue novel catalysts for various chemical transformations,^194^ while Boehringer Ingelheim seeks to accelerate drug discovery processes.^195^ These collaborations between quantum companies and industry leaders demonstrate the growing recognition of quantum technologies’ potential across multiple sectors,^196^ ranging from catalysis and energy storage to drug discovery and material science. By combining expertise and resources, these partnerships aim to drive innovation and shape the future of technology-enabled solutions.

In the subsequent sections, we delve into a detailed exploration of some of the most promising quantum algorithms in the context of heterogeneous catalysis. Additionally, we examine recent applications in periodic simulations that leverage quantum algorithms, such as calculating bulk lattice constants and simulating electronic band structures, as well as exploring molecule–surface interactions involving metals and metal oxides. These applications reflect collaborative efforts between academia and industry.

## 5 Quantum Computing Algorithms for Heterogeneous Catalysis Modeling

Quantum computing presents exciting opportunities for tackling complex chemistry problems, with several major quantum algorithms proving promising in this field. Among these, the variational quantum eigensolver (VQE)^60^ and quantum phase estimation (QPE)^197^ immediately stand out. In addition, algorithms due to Harrow–Hassidim–Lloyd (HHL)^76^ are used in uncertainty quantification in heterogeneous catalysis. HHL’s near-term counterpart, variational quantum linear solver (VQLS)^77^ could potentially be used for similar applications as well. Moreover, more versatile algorithms like linear combination of unitaries (LCU)^198,199^ and quantum singular value transformation (QSVT)^200,201^ are gaining traction for various applications in the field of quantum chemistry.^202^

In the rapidly evolving landscape of quantum computing, different algorithms serve distinct purposes tailored for specific types of quantum hardware categorized by its maturity in handling errors. We categorize these algorithms into two main groups: electronic structure methods suitable for near-term devices and those typically employed in the context of fault-tolerant quantum computers. Electronic structure methods for near-term devices, such as VQE, quantum subspace expansion (QSE), and variational quantum linear solver (VQLS), leverage the capabilities of noisy intermediate-scale quantum (NISQ) devices to address problems in quantum chemistry and material science.

- VQE is particularly effective for estimating the ground and excited-state energies of quantum systems, enabling detailed explorations of molecular structures and reaction dynamics.
- QSE enhances the accuracy of VQE by expanding the solution space, thereby improving estimates of energy levels and other observables.
- VQLS is adept at solving linear systems of equations that is suitable for uncertainty quantification in heterogeneous catalysis.

These methods utilize variational principles to optimize parameters and extract meaningful information about quantum states, making them accessible for current technologies despite their limitations in coherence and error rates. In contrast, methods designed for fault-tolerant devices, including QPE, HHL, and QSVT, are primarily aimed at achieving exponential speedups for large-scale problems.

- QPE excels at estimating the eigenvalues of quantum operators, which is fundamental for applications like determining ground-state energies in quantum chemistry.
- HHL is particularly useful for efficiently solving linear systems of equations, offering potential speedups for problems that can be represented in this form, e.g., uncertainty quantification.
- QSVT provides a versatile framework for various matrix operations, such as singular value decomposition and matrix inversion, which are essential for many quantum algorithms and applications.

To be able to run these algorithms, quantum error correction together with fault-tolerant quantum hardware is required.

QPE is the pioneering algorithm demonstrating efficient estimation of eigenvalues of unitary operators, offering insights into the energy spectra and electronic structures of quantum systems.^197^ Although QPE holds significant potential for chemistry applications, its implementation on current noisy intermediate-scale quantum (NISQ) devices^203^ faces challenges such as circuit depth, high error rates, limited qubit connectivity, and scalability. The estimated number of ancilla qubits (ω) required for phase estimation, given a precision of *n* bits and success probability *p*, is determined by Nielsen’s equation:^204^
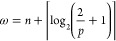
1

Despite recent progress, these methods involve large gate counts and the inability to perform a (large-scale) inverse quantum Fourier transform (QFT), requiring fault-tolerance,^205^ thereby poses challenges for near-term quantum computers. As quantum technologies advance and error correction techniques improve, QPE is in the long run expected to offer the most accurate and efficient solutions to chemistry problems on quantum computers. Alternative approaches are, however, required for practical chemistry simulations on the currently existing and upcoming quantum hardware.

Currently, no proven quantum speedup has been achieved with the variational quantum algorithms widely explored during the NISQ era. The largest molecular calculations in terms of qubit requirements and hardware resources are compiled in Table 2 of ref.^206^ Additionally, we have gathered various resource estimates for fault-tolerant quantum computing in chemistry, expressed in T/Toffoli gate counts, which vary based on estimator type and assumptions over time.^207−210^ For an overview of quantum chemistry software and hardware on quantum computers, we refer readers to a recent review.^211^ A full analysis of the advantages and limitations is beyond this work’s scope; however, we provide references to relevant metrics from surveys conducted by independent organizations, including the Unitary Fund. Last year’s survey results are available at ref.,^212^ and this year’s survey is currently underway.

The VQE, which integrates classical optimization techniques with quantum state preparation and measurement to determine ground-state energies of molecular systems, was introduced as a more practical option for near-term quantum computers.^60^ Recent developments have enabled studies on interaction energies between molecules and extended the treatment to periodic systems, which makes this algorithm relevant for heterogeneous catalysis reactions.^213^ In this section we will explore the potential of VQE, briefly touch upon a few VQE-inspired algorithms applied in the context of periodic systems, and discuss VQE’s application to excited states. Following that, other quantum algorithms relevant to quantum chemistry, including QPE, HHL, and QSVT, will be briefly discussed.

### 5.1 Near-Term (NISQ) Quantum Algorithms Relevant to Heterogeneous Catalysis

#### 5.1.1 Variational Quantum Eigensolver (VQE)

**Figure 3:**
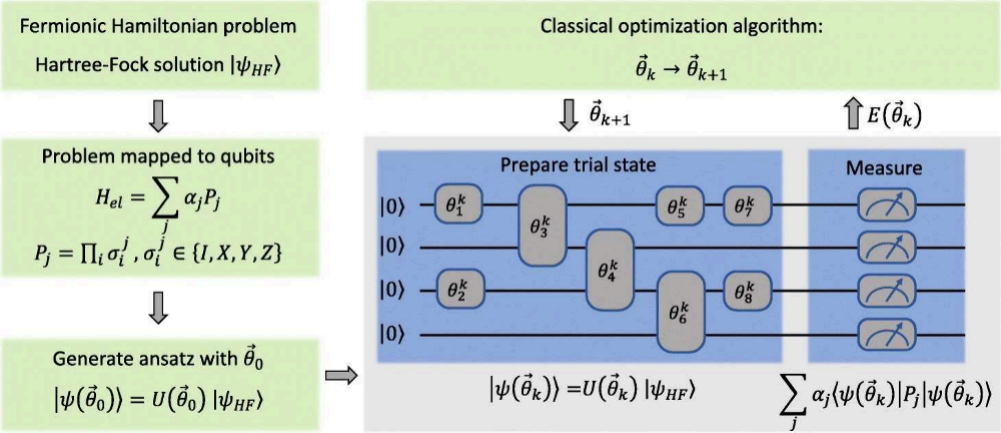
The schematic of the variational quantum eigensolver (VQE) method. This method combines classical (green) and quantum (blue) computing resources and optimizes the Hamiltonian energy ⟨ψ(θ⃗)|*H*_*el*_|ψ(θ⃗)⟩ by adjusting variational parameters θ⃗. It involves constructing a Fermionic Hamiltonian, mapping it to a qubit Hamiltonian, and initializing the wave function’s ansatz with θ⃗_0_. The trial state is prepared as a quantum circuit on a quantum computer. Iterative measurement of Hamiltonian terms helps update the parameters θ⃗_*k*+1_ via a classical algorithm until convergence is achieved. Reused from ref (214) under Creative Commons CC BY 4.0 DEED. Copyright 2022 The Author(s).

The VQE algorithm, originally proposed and realized by Peruzzo et al.^60^ in 2014 on a photonic quantum processor for computing the ground-state energy of HeH^+^, has emerged as a practical tool for calculations of ground-state energies in molecular and materials science using quantum computers. VQE belongs to the broader category of variational quantum algorithms (VQA),^215^ which are designed to solve a range of optimization problems. In essence, VQE employs the Rayleigh-Ritz variational principle to optimize a parametrized wave function or parametrized quantum circuit (PQC), ultimately minimizing the cost function, which for quantum chemistry problems is the electronic ground-state energy. VQE is a hybrid quantum–classical algorithm wherein there is a loop over classical and quantum processes, green and blue blocks, respectively in Figure 3. The quantum processor is used to evaluate the energy, through the expectation value of operators, while the classical computer runs the optimization algorithm that yields the parameter updates (θ⃗). In the NISQ era, VQE stands out as one of the best candidates for exploring the usefulness of quantum computers in chemistry simulations. In this discussion, we will provide an overview of the various steps and workings of the VQE. For more comprehensive reviews on VQE, interested readers can refer to excellent resources on the topic cited in the references:.^213,214,216^

A step-by-step workflow for implementing the VQE is given below:

1. **Step 1. Define the Hamiltonian operator:** Define the molecular Hamiltonian operator, *H*_*el*_, which describes the energy of the quantum system, in second quantization.
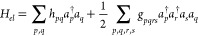
2Here, *a*^†^ and *a* are Fermionic creation and annihilation operators for placing or deleting an electron in spin orbitals, respectively. *h*_*pq*_ and *g*_*pqrs*_ are matrix elements of the one- and two-electron operators in the molecular orbitals basis that can be computed with *N*^5^ or lower computational cost on a classical computer.
2. **Step 2. Fermion-to-qubit mapping:** Next, Fermionic operators describing electron creation and annihilation (Step 1) are first mapped onto a qubit basis. This process, known as Fermion-to-qubit mapping, encodes molecular systems into quantum circuits. These circuits are essentially a series of operations represented by a linear combination of Pauli operators (acting on individual qubits) and coefficients. The key element in this mapping is the concept of occupation numbers. These numbers (0 for empty, 1 for occupied) represent the state of each electron orbital in the molecule, following the Pauli Exclusion Principle. Different mapping techniques like Jordan-Wigner,^217^ Bravyi-Kitaev,^218^ parity^219^ translate these occupation numbers into specific configurations of 0s and 1s on the qubits. Finally, the encoded electronic Hamiltonian of the molecule, represented by the eq 3, is constructed using Pauli operators/matrices (σ) acting on individual qubits (eq 4). These Pauli operators are built based on the occupation number information embedded within the qubits through the chosen mapping scheme.
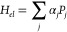
3where,
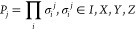
4Here, *I*, *X*, *Y*, and *Z* are identity, Pauli *X*, Pauli *Y*, and Pauli *Z* matrices (operators), respectively. (Can also be written as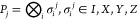).
3. **Step 3. Define the quantum circuit:** Then we define a parametrized quantum circuit, often denoted as *U*(**θ⃗**), where **θ⃗** represents a vector of variational parameters.
4. **Step 4. Prepare the trial state:** Use the quantum circuit to prepare a trial state |Ψ(**θ⃗**)⟩ by applying *U*(**θ⃗**) to an initial reference state (|0⟩), often chosen as the Hartree–Fock state:
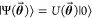
5
5. **Step 5: Calculate the expectation value:** Calculate the expectation value of the Hamiltonian *H*_*el*_ with respect to the trial state |Ψ(**θ⃗**)⟩:
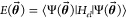
6
6. **Step 6: Minimize the energy:** Utilize a classical optimization algorithm (e.g., gradient descent or a variational optimizer) to minimize the energy *E*(**θ⃗**) by adjusting the variational parameters **θ⃗**:
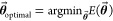
7
7. **Step 7: Extract the ground-state energy:** After optimization, the minimum energy *E*(**θ⃗**_optimal_) provides an estimate of the ground-state energy of the quantum system.

The iterative process, illustrated in Figure 3, continues until a satisfactory approximation of the ground-state energy is achieved. VQE leverages the quantum computer’s efficient trial state preparation while classically optimizing variational parameters to minimize the energy. It is a generic tool for quantum chemistry simulations allowing for various ansatzes for the unitary operator used in Step 3. The final energy estimate serves as an approximate solution to the optimization problem, constituting an upper bound to the true ground-state energy due to its variational nature. To practically estimate *E*(θ⃗), achieved through multiple samplings of the energy in Step 5, is in practice a bottleneck for the algorithm. The number of samples needed is crucial and scales with the desired precision, denoted by ϵ. This scaling comparison is notable: VQE exhibits a scaling of 1/ϵ^2^, contrasting with the 1/ϵ scaling of fault-tolerant algorithms like QPE and those approaching the Heisenberg limit. This distinction underscores the trade-off between precision and computational resources, prompting ongoing efforts to optimize sampling strategies and improve VQE’s efficiency. To validate the solution, comparisons can be made with the currently available quantum hardware or simulators, often with known exact solutions. When this is no longer possible and VQE calculations surpass what is classically computable, one may still examine consistency of solutions by validation with known symmetries or other system properties. This validation step ensures the reliability of the VQE-derived solution. The obtained energy and its derivatives can be applied for predictive purposes or decision-making based on the optimized parameters. For instance, it can help identify optimal molecular configurations or calculate interaction energies such as adsorption and reaction energies in heterogeneous catalysis.

#### 5.1.2 Excited States

Excited states play a crucial role in the study of heterogeneous catalysis, particularly in heterogeneous photocatalysis^220−223^ and plasmonic catalysis,^224−228^ due to their influence on the electronic structure and reactivity of catalysts. In photocatalysis, light excitation of a catalyst creates electron–hole pairs (excitations), which can participate in surface reactions. Understanding these excited states helps identify key pathways for charge separation, transfer, and recombination, which directly affect reaction rates and efficiency.^220^ The band structure of the catalyst dictates the energy levels at which such excitations can occur, and the alignment of the catalyst’s conduction and valence bands with those of reactant molecules determines whether an excited state can effectively drive the desired reaction. In plasmonic catalysis, excited collective oscillations of free electrons (plasmonic excitations) are generated when metal nanoparticles interact with light. These excitations can significantly enhance local electromagnetic fields at the catalyst surface, thereby increasing the reactivity of adsorbed molecules.^225^ The band structure of the plasmonic material affects how these surface plasmons are excited and how they interact with molecular orbitals of the reactants.^226^ The excited states in this case are critical for energy transfer and enhancing catalytic processes that rely on light-matter interactions. In both cases, the catalyst’s band structure 6.3 determines the nature and efficiency of excitation, and the excited states are central to understanding how energy is transferred or absorbed during catalytic processes, ultimately influencing reaction outcomes.

Due to their importance, several VQE extensions have been developed to compute excited states of a given Hamiltonian *H*. Quantum subspace expansion (QSE) is a method that resembles the configuration interaction approach in quantum chemistry and is particularly useful for mitigating noise errors in NISQ devices.^229^ Subspace-search VQE (SS-VQE) is an algorithm designed for this purpose, enabling the identification of excited states beyond the ground state.^230^ Additionally, multistate, contracted VQE (MC-VQE) is an extension of VQE that calculates excited states of the Hamiltonian *H*, resembling a simplified version of the SS-VQE algorithm.^231^ Moreover, two papers propose an alternative approach to compute excited states sequentially by incorporating overlap amplitudes between the ansatz state |ψ(θ⃗)⟩ and previously found eigenstates into the cost function of VQE.^232,233^ These extensions offer valuable tools for efficiently obtaining a comprehensive understanding of the excited states of quantum systems. This review will highlight two types of approaches to give an impression of what is currently possible. Quantum Subspace Expansion (QSE), utilized for computing excited states in periodic systems^234−237^ and addressing error mitigation,^56,238−240^ is briefly discussed below. Furthermore, we consider state-averaged approaches^241,242^ which provide a democratic description of both ground and excited states, as is valuable when studying photocatalytic reactions.

#### Quantum Subspace Expansion (QSE)

Quantum Subspace Expansion (QSE) is employed in quantum chemistry for calculating excited-state properties of molecular systems.^229^ It extends the framework of the VQE to capture the excited states by introducing a subspace spanned by a set of trial wave functions created from the optimized ground-state wave function. The excited states are then obtained by diagonalizing the Hamiltonian within this subspace. This method resembles the classical configuration interaction method and is applicable to a wide range of quantum systems.

Starting with a VQE, the trial wave function |Ψ(θ)⟩ is parametrized by a set of variational parameters θ. The ground-state energy *E*_0_ is minimized by optimizing these parameters. To extend this approach to excited states, QSE introduces additional parameters ϕ_i_ to create a subspace of trial wave functions. The excited states are then obtained by diagonalizing the Hamiltonian within this subspace, leading to the eigenvalue problem *H*|Φ_*i*_(ϕ)⟩ = *E*_*i*_|Φ_*i*_(ϕ)⟩, where *H* is the molecular Hamiltonian. The excited-state wave functions |Φ_*i*_(ϕ)⟩ are constructed as linear combinations of the ground-state |Ψ(θ)⟩ and the subspace generated via the operation of a set of operators (*O*_*j*_) on the ground state. Mathematically, this can be expressed as |Φ_*i*_(ϕ)⟩= (1 + ∑_*j*_ϕ_*j*_*O*_*j*_)|Ψ(θ)⟩, where *O*_*j*_ are the additional operators introduced to create excited states. The subspace expansion allows for a flexible representation of excited states, and can capture complex wave functions in a computationally efficient manner. QSE has been successfully applied to study various molecular systems, providing accurate and reliable results for excited-state properties in quantum chemistry simulations. Some examples of application of QSE to periodic systems, especially to the prototypical strong correlation benchmark model of hydrogen chains are discussed in Section 6.1.

#### State-Averaged Orbital-Optimized Variational Quantum Eigensolver (SA-OO-VQE)

**Figure 4:**
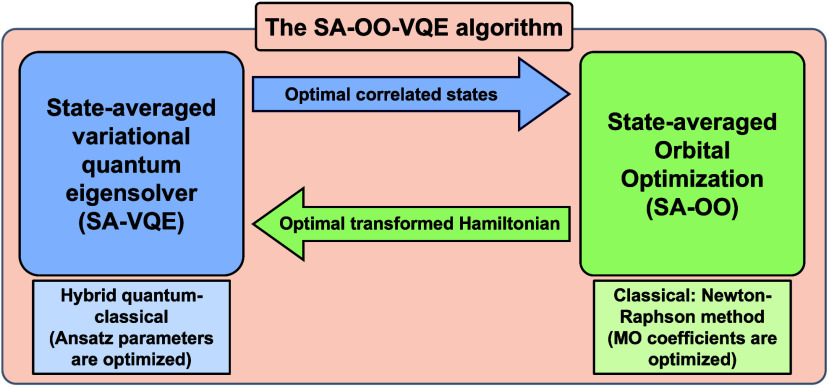
Schematic diagram of the SA-OO-VQE method, which achieves a balanced treatment of multiple electronic states in quantum computing for computational chemistry. It employs two algorithms in a cyclic manner: SA-VQE (hybrid quantum–classical, blue block) and SA-orbital-optimization (purely classical, green block). SA-VQE uses a quantum circuit to determine multiple low-lying eigenstates via state-averaged energy minimization. The correlated states are then transferred to SA-OO, which optimizes the molecular orbitals using the full orbital space to allow for further energy minimization. The process iterates, making the SA-OO-VQE algorithm a better scaling alternative to CASSCF for studying heterogeneous photocatalysis reactions on quantum computers.

In heterogeneous photocatalytic reactions, where both the catalyst and the initiation of the reaction by light play a role, being able to model both ground and excited states is crucial. Performing separate calculations for the ground state and excited state is time-consuming and does (unless the excited state has a different symmetry) not guarantee that the obtained excited state is fully orthogonal to the ground state as it should be for an exact solution. To address this issue and provide a democratic description of ground and excited states in photochemical reactions, the state-averaged orbital-optimized variational quantum eigensolver (SA-OO-VQE)^241^ method was developed. The main steps of the algorithm are explained in the diagram depicted in Figure 4. The method was later extended to be able to calculate analytical gradients and nonadiabatic coupling vectors, thereby enabling the study of excited-state dynamics.^242^ The method can also be used to detect conical intersection, a point in the potential energy surface where two electronic states are degenerate and nonadiabatic transitions between the states occur in photochemical reactions.^243^ So far, this approach has been primarily applied to a prototype of a single-molecule photoisomerization reaction. Our research group is currently exploring the application of this method to heterogeneous photocatalytic systems, specifically focusing on H_2_O dissociation on TiO_2_.

#### 5.1.3 Variational Quantum Linear Solver (VQLS)

The variational quantum linear solver (VQLS) is a quantum algorithm suited for near-term devices, leveraging a variational approach to approximate solutions for linear systems *A***x** = **b**.^77^ The vector **b** is encoded into a quantum state, while a parametrized quantum circuit (ansatz) encodes the solution vector **x**. A classical optimizer iteratively adjusts the parameters of this circuit to minimize a cost function—typically the residual error ∥*A***x** – **b**∥—until the quantum state approximates **x**. By measuring this state, specific components of **x** can be retrieved.

Compared to HHL (*vide infra*), which requires quantum phase estimation and works best with sparse, diagonalizable matrices, VQLS offers more flexibility for a broader range of matrices, including both sparse and dense types. This flexibility makes VQLS more practical for noisy, intermediate-scale quantum (NISQ) devices, which struggle with the high resource demands of HHL’s phase estimation. However, VQLS has limitations: it depends on classical optimization, provides only approximate solutions influenced by the ansatz and iteration count, and requires a quantum circuit for each optimization step, which can be resource-intensive for large systems.

Similar to the VQE, which approximates ground-state energies in quantum chemistry, VQLS uses a variational process, but it is specifically designed for solving linear systems of equations. Despite its challenges, VQLS offers a practical solution for quantum linear systems on NISQ devices, filling an important role until fault-tolerant quantum computers become available.

### 5.2 Fault-Tolerant Quantum Algorithms Relevant to Heterogeneous Catalysis

#### 5.2.1 Quantum Phase Estimation (QPE)

**Figure 5:**
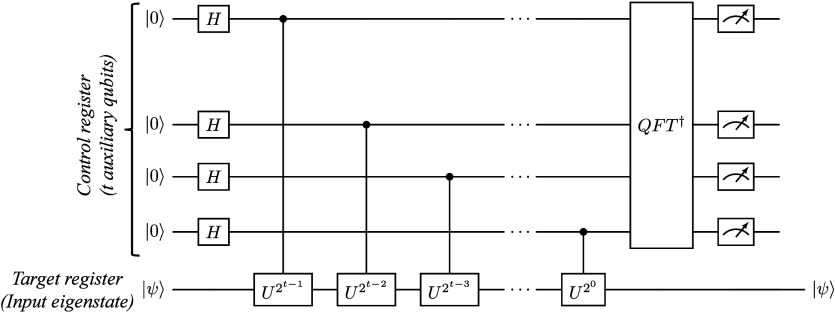
Quantum circuit for quantum phase estimation (QPE). The quantum circuit for the QPE algorithm involves several essential components. First, the control register containing *t* qubits are initialized to a uniform superposition of states using Hadamard (*H*) gates. Next, a sequence of controlled unitary operations is performed on the input state |ψ⟩, incorporating the unitary operator for which we aim to estimate the eigenvalues. Through these controlled operations, the input state becomes entangled with the control qubits, thereby encoding the phase information from the eigenvalues. Following the controlled unitary operations, the circuit proceeds to the quantum phase estimation process where an inverse quantum Fourier transform (QFT) is applied to extract the phase information from the control qubits. Finally, the outcome of the QFT is measured in the computational basis to provide an estimation of the phase, which corresponds to the eigenvalue of the unitary operator.

The Quantum Phase Estimation (QPE) algorithm is a quantum algorithm designed to efficiently estimate the eigenvalues of a unitary operator, which is typically represented by the Hamiltonian of the quantum system of interest in chemistry.^197^ The key idea behind QPE is to encode the eigenvalue information on the Hamiltonian into the phase of a quantum state. The algorithm requires two quantum registers: the control register, typically prepared in a superposition of states, and the target register, initialized in an eigenstate of the unitary operator (state with some considerable overlap with the ground state of the given molecule). The quantum circuit used to illustrate the different steps of the QPE algorithm is shown in Figure 5.

The QPE algorithm essentially involves two main steps:

1. **Phase kickback**: In this step, a controlled unitary operation is applied between the control and target registers, where the control qubits are set to a superposition of states using Hadamard (*H*) gates. The controlled unitary operation effectively “kicks back” the phase of the target register’s state based on the eigenvalue corresponding to the eigenstate of the Hamiltonian. This phase information is encoded in the quantum state of the target register.
2. **Inverse quantum Fourier transform**: After phase kickback, the inverse quantum Fourier transform (QFT) is applied to the control register. The inverse QFT transforms the superposition of states in the control register into a state whose phases represent the eigenvalues of the Hamiltonian. Measuring the control register then provides an estimation of the eigenvalues.

By repeating the QPE algorithm multiple times and using postprocessing techniques, more accurate estimates of the eigenvalues can be obtained. These estimated eigenvalues directly correspond to the energy levels and electronic properties of the molecular system of interest. Implementing QPE on current NISQ devices faces enormous challenges,^244^ stemming from circuit depth, error rates, and qubit connectivity, which limit the accuracy achievable with the inverse quantum Fourier transform (QFT^†^). To address these limitations, much research is focused on advancing the error mitigation techniques and otherwise optimizing implementations.^245^ Both such algorithmic advances as well as hardware scale-up will be needed to bring the potential of the QPE algorithm for quantum chemistry applications to life. As quantum technologies keep maturing, QPE is expected to become valuable in quantum chemistry research, and increasingly suitable to address the challenges in describing the strongly correlated electronic states encountered in the discovery of new catalytic materials and in studying chemical reaction mechanisms.

#### 5.2.2 Harrow–Hassidim–Lloyd (HHL) algorithm

The HHL (Harrow–Hassidim–Lloyd) algorithm^76^ is a quantum algorithm specifically designed for solving linear systems of equations, which play a crucial role in various scientific and engineering applications, including quantum chemistry. In the context of heterogeneous catalysis, the HHL algorithm has been proposed to be used in uncertainty quantification (UQ).^73−75^ UQ is the process of assessing and representing uncertainties in model simulations such that their impacts on the quantities of interest can be determined.

**Figure 6:**
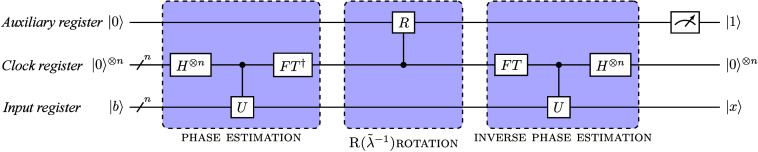
Quantum circuit for implementing the Harrow–Hassidim–Lloyd (HHL) algorithm. The HHL quantum circuit involves three main steps: quantum phase estimation (QPE), rotation, and inverse QPE. In the QPE step, the algorithm encodes a classical vector into a quantum state and, using QPE, it estimates the eigenvalues of a given linear system’s matrix. In the rotation step, the quantum state is rotated based on the eigenvalue estimation. Finally, in the inverse QPE step, the algorithm uncomputes the eigenvalue estimation, and the result of the quantum computation is the solution to the linear system, which can be efficiently obtained from the quantum state amplitudes.

The HHL algorithm can efficiently solve the linear system *Ax* = *b*, where *A* is a Hermitian matrix representing the quantum system’s Hamiltonian, *x* is the unknown vector representing the solution, and *b* is the input vector encoding the problem to be solved. In short, the HHL algorithm, employs QPE to encode the eigenvalues of matrix *A* into the quantum state and then performs controlled rotations to extract the desired solution *x*. The final step involves performing measurements on the quantum state, yielding the solution to the linear system with high probability. To provide a bit more detail, the HHL algorithm follows a structured sequence of three steps designed to solve linear systems (Figure 6). The first step is the QPE (Section 5.2.1), which allows one to approximate the eigenvalues of a Hermitian matrix *A* when the input state |*b*⟩ is one of its eigenvectors. The eigenvalues λ_*j*_ and eigenstates |*u*_*j*_⟩ of *A* are computed with certain precision through QPE. The clock register stores the values of the phase of the eigenvalues of the *A* matrix after the QPE. Subsequently, we move to the second step, where a controlled rotation, dependent on λ_*j*_, is implemented. To achieve this, a third auxiliary register initialized as |0⟩ is introduced, and a controlled σ_*y*_-rotation is performed based on our λ_*j*_ estimate stored in the clock register. When this is successful, the result resembles the answer |*x*⟩ that we are looking for. In the third step, the uncomputation is done by using inverse QPE i.e., we undo the QPE to set the register that contained the estimate back to |0⟩. In this step, the qubits in the clock register and the *b*-register are disentangled and the input-register |*b*⟩ stores the solution |*x*⟩.

The application of HHL algorithm in microkinetic modeling stems fromt he fact that one can represent the rate equations of a catalytic reaction network as a system of linear equations in which, *A* is the rate constant matrix, capturing reaction rate constants that describe reaction pathways and relationships among species, *x* is the coverage vector, representing the fractional surface coverage of each species involved in the reaction and *b* is the rate vector, encapsulating the net production or consumption rates of the species.^73^ Using the HHL algorithm in this context would allow one to compute *x* efficiently, but its value goes beyond just finding solutions—it can also serve in uncertainty quantification. Since reaction rates (elements of *A*) are often uncertain due to experimental variations or intrinsic limitations in reaction models, quantum approaches like HHL can incorporate uncertainty directly by solving *Ax* = *b* across distributions of potential *A* matrices. This can reveal how variability in reaction rates impacts surface coverage and reaction outcomes. For example, one could repeatedly solve for *x* with different probabilistic variations in *A*, enabling an uncertainty quantification framework that assesses how fluctuations in rate constants affect the system.

While the HHL algorithm holds potential for quantum chemistry applications, especially in uncertainty quantification for heterogeneous catalysis and other scientific fields, its implementation on current NISQ devices faces challenges similar to other fault-tolerant quantum algorithms like QPE.^246^ The algorithm offers exponential speedup in solving linear systems of equations, particularly beneficial for sparse or structurally specific matrices,^247^ closely tied to the QPE algorithm for efficient eigenvalue determination. However, for solving general large-scale linear systems, the HHL algorithm offers a polynomial, not exponential, speedup over classical methods.^248^ Despite its theoretical potential, practical implementation is hindered by issues such as quantum gate errors, decoherence, and the need for error correction. Nevertheless, with the advancements in quantum computing hardware and error correction techniques, the HHL algorithm is anticipated to make positive contributions in uncertainty quantification for computational heterogeneous catalysis.

#### 5.2.3 Quantum Singular Value Transformation (QSVT)

The spectral theorem in quantum chemistry facilitates the diagonalization of Hermitian operators, simplifying complex quantum systems by expressing these operators as a sum of eigenvalues and orthogonal projectors. Singular value decomposition (SVD) is a valuable mathematical tool widely used in quantum chemistry for matrix analysis, allowing dissection of matrices into key components: unitary matrices *U* and *V*^†^, and a diagonal matrix Σ with singular values. Unlike the spectral theorem, SVD is applicable to rectangular matrices, making it more versatile for application in quantum chemistry where rectangular matrices often occur. Quantum Singular Value Transformation (QSVT),^200^ a quantum algorithm analogous to SVD, applies polynomial transformations to singular values using quantum computers, serving as a generalization of Quantum Signal Processing (QSP).^249−251^ QSP systematically applies quantum gates to qubits, initially designed for square matrices and extended to nonsquare matrices through QSVT. Together, QSP and QSVT form a versatile framework recognized as the ’grand unification of quantum algorithms,’^201^ providing a unifying foundation for fault-tolerant quantum algorithms, such as QPE and HHL algorithms discussed above, showcasing its potential in advancing fault-tolerant quantum computing methodologies. While delving into the details of how each algorithm is constructed within this framework lies beyond the scope of this review, it is noteworthy that the QSVT framework provides an abstract and versatile approach that underlies the development of these fault-tolerant quantum algorithms. Despite its potential, the QSVT algorithm faces challenges in constructing accurate polynomial approximations for desired functions, particularly as matrix size and complexity increase. Researchers actively explore strategies to enhance the applicability and efficiency of QSVT in quantum algorithms and simulations.^252−255^

### 5.3 First Quantization and Plane Waves

It has been already discussed that DFT is a powerful tool for understanding the electronic structure and reactivity of catalyst surfaces in heterogeneous catalysis.^21,256^ By employing periodic boundary conditions and expanding the wave functions in terms of plane waves, DFT can relatively accurately capture the periodicity of the crystal lattice and the interactions between the catalyst surface and adsorbates. This allowed for the investigation of key processes involved in catalytic reactions, such as adsorption, activation, and reaction pathways. Recently, there have been proposals to utilize plane waves within the framework of first quantization for fault-tolerant quantum computing.^257−259^ First quantization refers to the direct description of a quantum system in terms of its wave functions and operators, without resorting to the second quantization formalism (creation and annihilation operators) commonly used in quantum chemistry. The use of plane waves in first quantization approaches aims to harness their periodic nature and Fourier transform properties to efficiently represent and manipulate quantum states in large-scale quantum computations. Two such examples are discussed in Section 6.4.3 and Section 6.4.4.

## 6 Application of Quantum Computing Algorithms for Simulating Periodic Systems

When modeling periodic systems, integrals are to be evaluated in reciprocal space, typically at points within the first Brillouin zone (BZ). Calculations performed at the central point of the BZ, denoted as the Γ-point, is akin to modeling molecules. In systems with periodic boundary conditions (PBC), integral calculations extend across the BZ, requiring assessment at multiple *k*-points. The accuracy increases with the number of *k*-points, but such an increase also leads to a higher computational cost. For bulk materials PBC are needed in all three spatial dimensions and balancing accuracy and computational cost is crucial. For surfaces, which only exhibit periodicity in the *x*- and *y*-directions one may devise a dedicated approach, but simpler is to introduce a vacuum in the *z*-direction. The latter approach allows for straightforward use of the full periodic plane wave approach, but requires careful consideration of *k*-point sampling. A surface model in DFT requires a vacuum in the *z*-direction to prevent interactions with its periodic replica. With a sufficiently large vacuum a realistic representation of the surface can be obtained. If the vacuum is sufficiently extended (around 15 Å), modeling surfaces with just one *k*-point along the *z*-axis is feasible. Surfaces, lacking the full symmetry of bulk systems, require larger unit cells, increasing computational demands. In addition, the high density of electronic states near the Fermi level found in surface models does further increase computational costs.^256^ DFT implies the use of a density function approximation (DFA) for the exchange-correlation energy. For molecules such DFAs usually incorporate a fraction of nonlocal “exact” exchange to improve their performance. Thus, going beyond pure DFT, various hybrid DFAs demonstrate improved performance relative to the best (semi)local functionals, especially in systems with strong correlation. However, using nonlocal exchange for electronic band structures and molecule–surface interactions is computationally demanding and the improvement in performance is often problem-specific.^33^ The surge in interest in quantum computing, coupled with advancements in quantum hardware and software, has prompted studies addressing electronic structure calculations in periodic systems and molecule–surface interactions relevant to heterogeneous catalysis (Table 1). This offers promising avenues to overcome computational and methodological challenges encountered in the DFT approaches. Variational quantum algorithms^215^Section 5.1.1 rooted in the second quantization of the electronic Hamiltonian have also been considered for simulating periodic systems as we will discuss below. Quantum simulations using plane wave basis sets Section 5.3, while not offering substantial advantages in the near-term,^257^ are also discussed in this review, highlighting the potential of quantum computing both in the near-term and the fault-tolerant era. To learn more about both basis sets and the difference between molecular and periodic simulations, in the context of quantum computers the readers are referred to this resource.^264^

**Table 1:** Overview of Periodic Systems Analyzed in the Literature, Including the Hardware Platform, Number of Qubits, and Quantum Algorithms Employed^a^

Periodic System	Platform	Qubits	Algorithm
**Hydrogen chains**			
Linear H-chain^234^	Emulator	-	VQE/K2G, VQE/QSE
Linear H-chain^235^	Emulator	8 (4 tapered)	TransQSE
Linear H-chain^236^	Emulator	12	VQE
H_2_ dimer^236^	Emulator	8	VQE
**Band structures**			
H_2_O/Mg(001)^260^	Superconducting qubits (IBM)	2, 10	VQE (EF)
O_2_/Pt(111)^261^	Trapped ions (Quantinuum - H1)	6, 8	VQE
Fe^235^	Superconducting qubits (IBM)	2	VQE
Battery materials^259^	Cost estimation only	-	QPE (qubitization)
Transition metal oxides (NiO and PdO)^258^	Cost estimation only	-	QPE (Wannier and Bloch orbitals)
**Embedding**			
NV center in diamond^262^	Emulator	6^b^	QPE (QDET)
NV center in diamond^262^	Emulator	2	VQE (QDET)
NV center in diamond^262^	Superconducting qubits (IBM)	2	VQE (QDET)
CO_2_ capture^263^	Emulator	4–16	VQE (DMET)
**Kinetics and uncertainty quantification**			
CO oxidation on Pt(111)^73^	Emulator	3	HHL
CO oxidation on Rh(111)^74^	Emulator	3	HHL
Hydrogen-air combustion^75^	Emulator	3	HHL

a For fault-tolerant quantum algorithms, such as QPE, resource or cost estimates are provided where available. Entries without reported qubit counts are retained for consistency. In this context, “emulator” (also referred to as a simulator) denotes quantum algorithms classically emulated on conventional computing systems. Abbreviations: VQE (variational quantum eigensolver), QSE (quantum subspace expansion), EF (entanglement forging), QPE (quantum phase estimation), QDET (quantum defect embedding theory), HHL (Harrow–Hassidim–Lloyd algorithm).

b Number of ancilla qubits.

### 6.1 Hydrogen Chains

Hydrogen atom chains are widely used as model systems in quantum chemistry and condensed matter physics for studying strong correlation effects.^265−267^ These chains consist of linear arrays of hydrogen atoms, each contributing a single electron. Due to their simplicity, hydrogen chains provide a clean environment to isolate and investigate the effects of electron–electron interactions. When hydrogen atoms are spaced at intermediate distances, the electrons become strongly correlated, making the system difficult to describe using mean-field theories like Hartree–Fock. This strong correlation emerges because the electrons interact strongly with each other, leading to phenomena that simpler methods fail to capture. Hydrogen chains, particularly when modeled as infinite systems with periodic boundary conditions, serve as useful approximations for periodic systems with strong correlations, such as Mott insulators in condensed matter physics.^267^ Their relatively simple structure, combined with the challenging correlation effects, make them ideal for benchmarking advanced methods.

The VQE algorithm, described in (Section 5.1.1), has shown to be applicable for computing ground-state energies of molecular systems. Building upon this success, researchers have extended the VQE algorithm to be able to simulate periodic systems. As a proof of principle that can be studied with a small number of qubits, initial applications of the VQE to periodic systems focused primarily on the ground-state properties of one-dimensional hydrogen chains.^234−236^

Using PBC and the Hartree–Fock method to obtain orbitals, one-dimensional hydrogen chains were studied using VQE by Liu and co-workers.^234^ They compared the performance of three ansatzes: unitary coupled cluster singles and doubles (UCCSD),^268^ unitary coupled cluster generalized singles and doubles (UCCGSD)^269^ and adaptive derivative-assembled pseudotrotter (ADAPT)^270^ method. Looking at the potential energy curve as a function of the H–H lattice distance, they found UCCSD-VQE and ADAPT to deviate significantly from the full CI reference result, while the absolute error of UCCGSD-VQE ansatz was acceptable, but still one to 2 orders of magnitude larger than in nonperiodic simulations. The problem in these approaches was found to be due to the imaginary component of the periodic wave function which invalidates an assumption made when deriving the energy optimization algorithm. To overcome this problem and to be able to model periodic systems at various *k*-points in the Brillouin zone, Liu et al.,^234^ proposed two modified VQE algorithms: VQE-K2G and VQE/QSE. The VQE-K2G approach involves the conversion of HF orbitals at sampling *k*-points in a unit cell into real orbitals at Γ-point in the corresponding supercell. Subsequently, the wave function and Hamiltonian are then defined in the real space so that the optimization method is valid again. This change of basis allows VQE-K2G for periodic systems to match the accuracy of VQE-K2G accuracy to VQE for molecular systems. The second approach, combining VQE with QSE, referred to as VQE/QSE was also proposed to enhance the accuracy of VQE. In VQE/QSE, a reference state is prepared using VQE, and the ground-state wave function is obtained by diagonalizing the Hamiltonian sampled in the linear-response space of the reference state. VQE/QSE could provide a reliable estimation of the exact wave function, provided that VQE can generate a suitable reference state. Their calculations demonstrate that both VQE-K2G and VQE/QSE approaches provide reasonable results for describing the potential energy surfaces of one-dimensional hydrogen chain with the SVZ (While the authors mention SVZ, we think it is split valence polarized (SVP) because we could not find a SVZ basis set within PySCF basis set library.^271^) basis set together with GTH pseudopotentials.^272,273^ It was also noted that for achieving converged results with practically relevant systems and also for the long-term, other type of basis sets, such as plane waves, should be used. Among these ansatzes, UCCGSD-VQE was found to be more stable than UCCSD-VQE. However, in the comparison between UCCGSD and ADAPT, no clear winner emerges due to the trade-off between flexibility, accuracy, and cost.

A hybrid quantum–classical algorithm, extending the unitary coupled cluster (UCC) framework, was utilized to calculate the electronic structure of periodic systems (linear hydrogen chains and dimer hydrogen chains), determining ground states and quasiparticle band structures.^236^ A variation of QSE was employed for the computation of the quasiparticle band structure. This approach shares conceptual similarities with ionization-potential/electron-attached EOM-CC (IP-EOM-CC, EA-EOM-CC),^274^ a variant of equation-of-motion coupled cluster (EOM-CC).^275^ The algorithm’s efficiency was validated in simulating the hydrogen chain for both weakly and strongly correlated electronic structures using the VQE.

In another study, the adaptation of the UCC ansatz to periodic boundary conditions is presented in both real space and momentum space representations showing the application of VQE in the simulation of solid-state crystalline materials.^235^ This adaptation involves the direct mapping of complex cluster operators to a quantum circuit ansatz, capitalizing on the reduced number of excitation operators and Hamiltonian terms due to momentum conservation. A translational Quantum Subspace Expansion method (TransQSE) is proposed for the localized representation of the periodic Hamiltonian. The investigation includes a comparative analysis of accuracy and computational costs across various geometries for 1D chains of dimerized hydrogen, helium, and lithium hydride, incorporating an increasing number of momentum space grid points. Additionally, VQE calculations are demonstrated for two-dimensional and three-dimensional hydrogen and helium lattices. The UCCSD-PBC ansatz is identified as the most favorable in the momentum space representation, considering circuit depth. Notably, the adoption of a smaller supercell, proves effective in trading accuracy against the expensive scaling associated with the full UCCSD-PBC approach. However, the authors emphasize that this strategy is applicable exclusively to insulating systems, where orbital occupation remains constant across different *k*-points. For metals, characterized by band lines crossing the Fermi level, the transformation introduces complexity, mixing occupied and virtual orbitals, making the preparation of the reference state nontrivial.

Furthermore, Mizuta et al.^276^ introduced an enhanced version of the DeepVQE protocol,^277^ emphasizing the efficient computation of low-energy eigenstates, with a particular focus on simulating periodic materials. The refined DeepVQE approach was specifically designed and tested using a periodic hydrogen chain system for its simplicity. Advancements over the initial DeepVQE proposal involve optimized strategies for handling periodicity, ensuring precise simulations of periodic materials. In addition, the updated protocol integrates advanced techniques to minimize the number of parameters in the quantum circuit, enhancing the efficiency of computations for low-energy eigenstates.

These studies share a common thread in their utilization of the VQE adapted for systems governed by PBCs. Notably, each study applied their respective methodologies to compute the energy of the one-dimensional hydrogen chain, with some extending their analysis to encompass two- and three-dimensional model systems. An additional noteworthy parallel lies in the incorporation of the QSE method for calculating excited-state energies across these investigations. The collective findings signify an increasing interest in quantum computing methodologies tailored for periodic systems, as evidenced by the adaptation of established algorithms from molecular studies. While these studies primarily serve as a proof of concept, there is a growing imperative to extend investigations beyond hydrogen chains to more realistic systems. The evolving landscape of quantum algorithms promises insights into their performance compared to established methods like DFT and their applicability in modeling various periodic systems. This exploration aims to uncover advantages in accuracy, computational efficiency, and adaptability across different material types.

### 6.2 Bulk bcc-Fe: Quantinuum–Nippon

**Figure 7:**
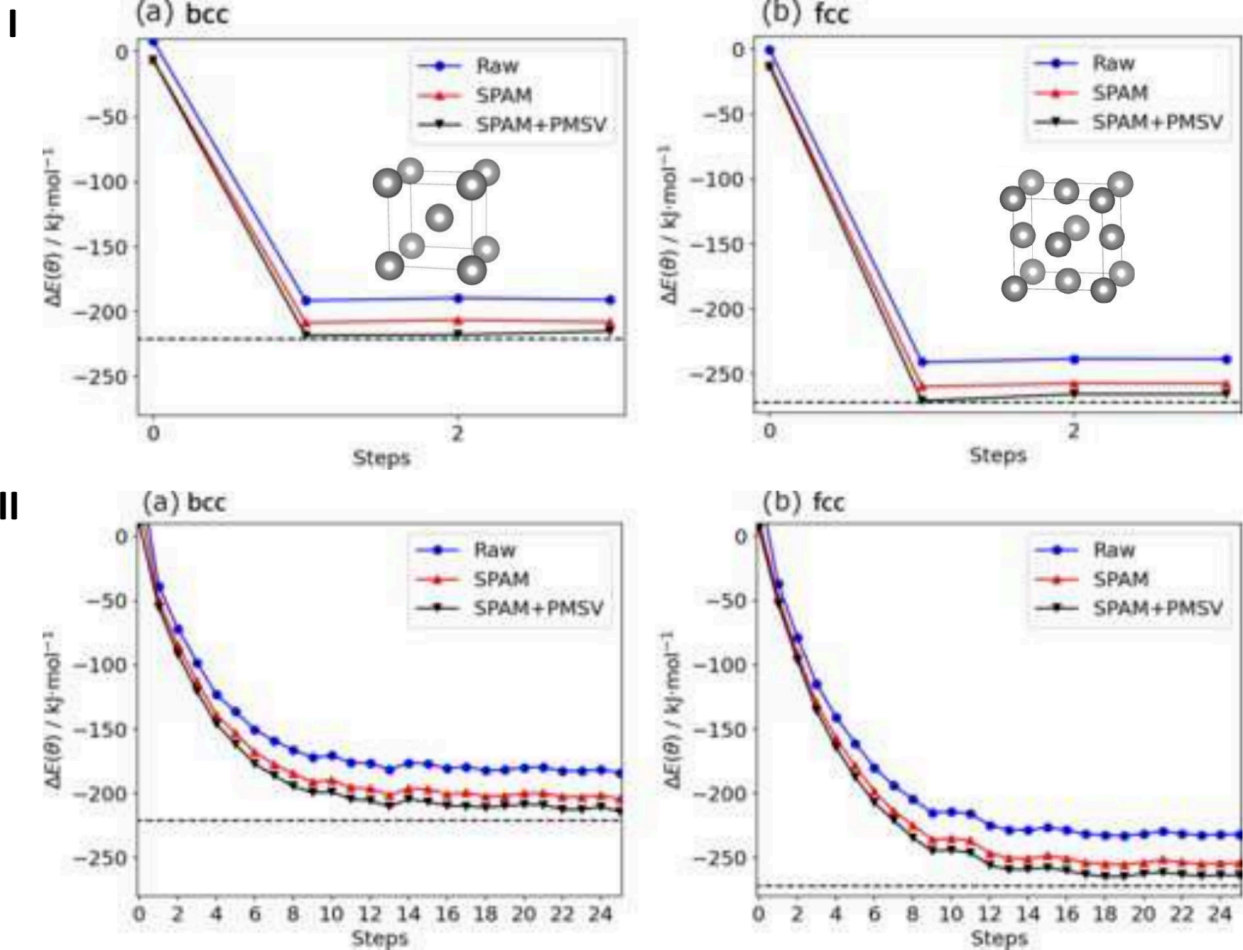
Lattice constants of bcc and fcc iron crystals. Panel I: Evolution of energy *ΔE* during the Rotosolve optimization process. Panel II: Evolution of energy *ΔE* during the stochastic gradient descent optimization process for both bcc and fcc iron crystals (crystal structure shown in inset). Blue circles represent hardware results from the *ibmq*_*casablanca* device. The dashed black lines indicates *ΔE* for the model Hamiltonian. Application of noise mitigation schemes SPAM (red triangles) and SPAM+PMSV (black triangles) (see text) is shown, which presents raw and noise-corrected *ΔE* for each optimization step to illustrate noise mitigation effects. Reused from ref (278) under Creative Commons CC BY 4.0 DEED. Copyright 2022 The Author(s).

The bulk lattice constant refers to the equilibrium lattice parameter or the optimal interatomic distance in a crystalline material in its bulk or three-dimensional form. It is a fundamental property of a crystal and is often a key parameter in characterizing its structure. Computationally, to find the equilibrium lattice constant, one performs calculations for different lattice constants, varying the interatomic distances. The lattice constant at which the total energy is minimized corresponds to the equilibrium lattice constant. Quantum hardware calculations were conducted for solid-state model systems under PBCs, focusing on a distorted hydrogen chain and fcc and bcc iron crystals (Inset of Figure 7.I).^278^ Utilizing two-qubit one-parameter ansatz, the translational quantum subspace expansion (TransQSE) method^235^ was applied to the hydrogen chain, while the PBC-adapted VQE method was employed for iron crystals. Variational optimization employed classical algorithms, Rotosolve^279^ and Stochastic Gradient Descent (SGD),^280^ for both methods. Quantum hardware experiments were executed on the IBM Quantum Falcon processor, specifically *ibmq*_*casablanca*. Noise mitigation techniques, including state preparation and measurement (SPAM)^281^ and partition-measurement symmetry verification (PMSV),^278^ significantly improved accuracy compared to exact values obtained through classical simulations (Figure 7.I and II). Despite the simplicity of the model systems, these results serve as a foundational step for advancing quantum chemical calculations on quantum computers, with potential improvements anticipated as quantum hardware evolves to accommodate larger basis sets and *k*-point grids for more accurate total energy estimates.

### 6.3 Electronic Properties: Band Structures

Band structures are fundamental to understanding the electronic properties of solid materials and play a crucial role in heterogeneous catalysis. One key concept linking band structure to catalytic performance is the *d*-band center model,^19^ which has been highly successful in predicting and optimizing the catalytic behavior of transition metals. This model directly correlates the position of the *d*-electron states relative to the Fermi level with the strength of adsorption and reactivity on metal surfaces. A higher *d*-band center leads to stronger adsorption interactions, while a lower *d*-band center results in weaker binding. Since catalytic efficiency depends on achieving an optimal balance between strong and weak adsorption (Sabatier’s principle), tuning the band structure through techniques like alloying or nanostructuring becomes essential for improving catalytic performance.^282^ This concept is particularly important in industrial applications, including fuel cells, hydrogenation, and CO^2^ reduction. Moreover, band structures are also critical in heterogeneous photocatalysis,^283−286^ where they govern the interaction between the photocatalyst and light. The ability of a photocatalyst to absorb light, generate electron–hole pairs, and facilitate charge carrier mobility depends on its band structure. By engineering the band structure, one can optimize processes like water splitting, pollutant degradation, and CO^2^ reduction. Given the crucial role of band structures in both heterogeneous catalysis and heterogeneous photocatalysis, we extend our discussion to include the calculation of band structures using quantum computing methods, as these are relevant for modeling in heterogeneous catalysis.

**Figure 8:**
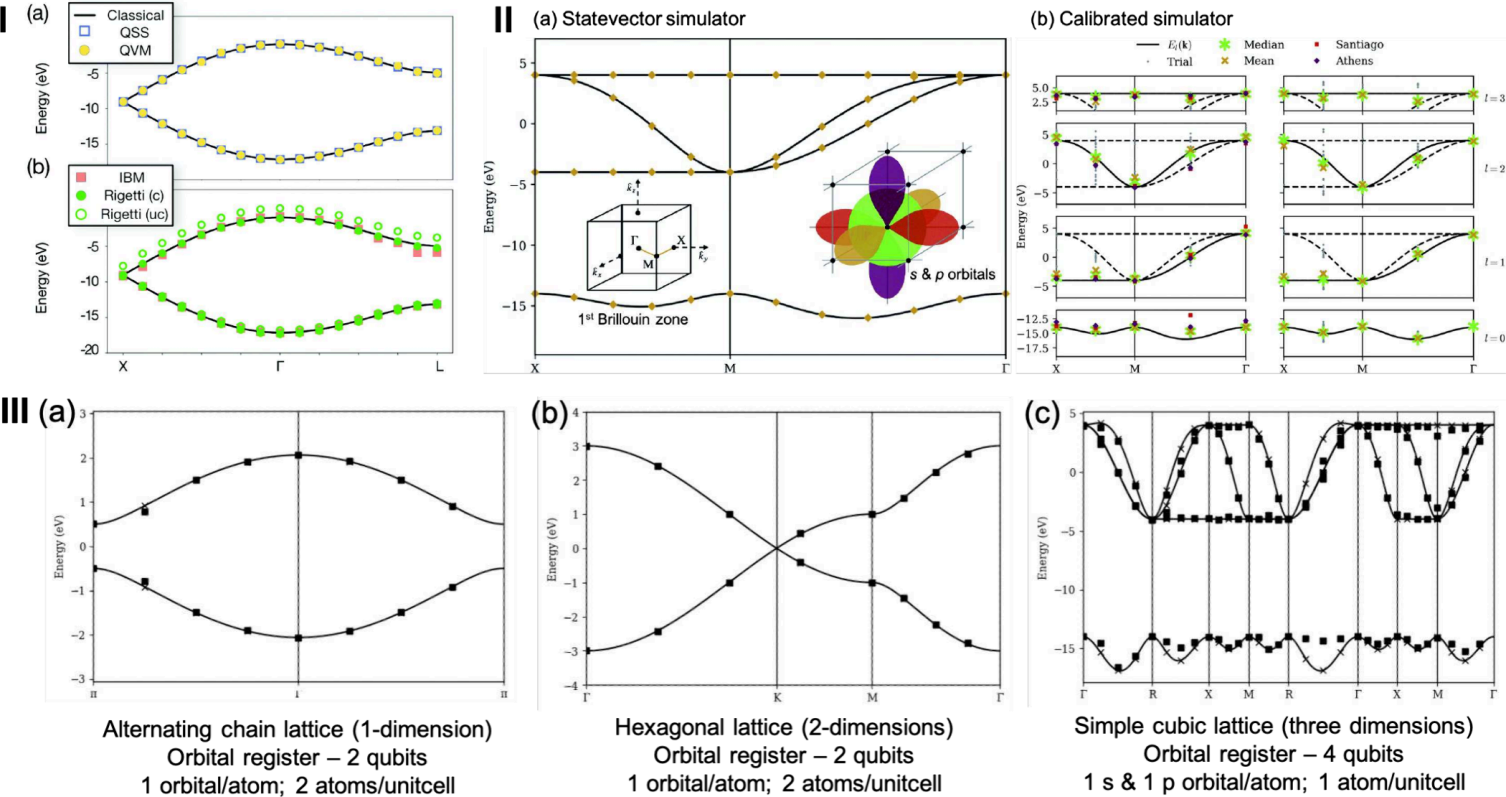
Electronic band structures. (I) (a) Comparison of the two-band electronic structure of silicon along the X-G-L line obtained through classical diagonalization (black solid line), a hybrid quantum–classical algorithm on a quantum state simulator (QSS, blue squares), and a quantum virtual machine (QVM, yellow circles). (b) Similar comparison as in (a) but executed on the quantum processing units (QPUs) of IBM (red squares) and Rigetti (green circles—before and after correction for readout errors). (II) (a) The band structure of a simple cubic lattice along the high-symmetry path. Solid curves: classical (exact) diagonalization. Diamonds: median optimization result from a noiseless statevector simulator. (b). Simulating low-fidelity qubits, along with rudimentary calibration. Left column: raw optimization results. Right column: energy obtained by QPE refinement. Gray dots: results from each of 32 trials, with each band given its own row (*l* = 0, 1, 2, 3). Asterisks and crosses: the median and mean, respectively. Squares and diamonds: energies measured on quantum devices *ibmq*_*santiago* and *ibmq*_*athens*, respectively, using the least-error optimization results obtained with the calibrated simulation data. (III) Band structures of model systems in one, two, and three dimensions with *N* = 8 for each dimension and 3 qubits per dimension. Analytically calculation using the standard classical algorithm (solid curves). Values estimated through the simulation of the quantum algorithm (squares). Values obtained under ideal conditions featuring perfect optimization and no sampling noise (crosses). Figure used with permission from refs (287−290) under Creative Commons CC-BY 3.0 (refs (287) and (288)) and Creative Commons CC-BY 4.0 (ref (290)).

Nardelli and co-workers^287−290^ explored the evaluation of band structures, an essential aspect for understanding electronic properties of solid materials. They developed an approach to compute properties of periodic solids, exemplified by calculating the band structure of silicon using the VQE algorithm on Rigetti Aspen and IBMQ Armonk quantum hardware.^287^ Comparative calculations were performed on Quantum Virtual Machine (QVM) and Quantum State Simulator (QSS). While the quantum-computed bands generally align with classically computed bands, slight deviations are observed near high-symmetry points (G and L) for Rigetti and IBM, respectively (Figure 8I). The authors suggest that various sources of errors, including probabilistic aspects and noise simulation, may contribute to these discrepancies, with gate noise and readout errors influencing measured energy and shifting expectation values toward different eigenstates.

A hybrid quantum–classical algorithm was designed for determining the band structure of periodic systems described by tight-binding models.^288^ While no quantum advantage is to be expected for tight-binding as it is a method with low-order scaling (at most cubic) and a small prefactor, such models are useful to explore quantum–classical algorithms. To illustrate the correctness of the approach, the algorithm is applied to compute the band structure of a simple-cubic crystal with one *s* and three *p* orbitals per site, serving as a model for polonium. The computations include simulations on quantum simulators with varying noise levels, concluding with experiments conducted on IBM quantum computers (Figure 8II). The findings demonstrate the algorithm’s reliability in low-noise environments, functional adaptability to present-day noisy quantum computers, and a scaling complexity similar to classical counterparts.

The next study explores an approach to quantum computing in materials science by focusing on the calculation of a periodic system’s single-electron band structure. Traditional methods involve constructing unique Hamiltonian operators for each *k*-point, requiring numerous optimizations to generate a single band. The proposed approach adopts a direct space method, utilizing a hybrid qubit mapping to construct a single Hamiltonian and cost-function suitable for solving the entire electronic band structure.^290^ The results of band structures calculated for model systems in one, two, and three dimensions are shown in Figure 8III. This approach supposedly simplifies the quantum algorithm for band structure calculations, offering technical and conceptual advantages over previous methods proposed by this group.

Other researchers have proposed a new quantum algorithm, EOM-ADAPT-C, specifically designed to calculate band structures in periodic systems.^291^ This method leverages the equation-of-motion (EOM) theory and builds upon existing techniques like ADAPT (adaptive derivative-assembled pseudotrotter)^270^ and K2G methods^234^ discussed above. EOM-ADAPT-C utilizes a unique variation of the VQE called ADAPT-VQE,^270^ which constructs the wave function adaptively using a complete set of anti-Hermitian operators. Following the ground-state calculation with ADAPT-C, this algorithm employs EOM theory^292^ within QSE^229^ to determine quasiparticle energies and ultimately the band structure. Notably, the method incorporates projected excitation operators to ensure a specific condition is satisfied. In contrast to tight-binding approach that are already very efficient on classical computers and have a limited accuracy, the higher level of theory and more accurate results that can be achieved provide some perspective on quantum advantages in the future. The adequacy of EOM-ADAPT-C has been demonstrated by calculating the band structures of silicon and diamond using a quantum simulator, with results matching well with established EOM-CCSD calculations. These explorations serve as a starting point for investigating the calculation of band structures for strongly correlated catalytic materials using quantum computing methods. Continued advancements in this area are anticipated, given the crucial role of band structures in the d-band center model and heterogeneous photocatalysis.

### 6.4 Heterogeneous Catalysis: Chemical Reactions at Surfaces

#### 6.4.1 H_2_O Dissociation on Mg(001): IBM–Boeing

A novel method for modeling surface reactions on quantum computers was introduced,^260^ featuring active-space orbital selection based on the electronic density and its effect on energy, employing the VQE for the calculation of expectation values. Efficiency is enhanced by evaluating the active-space Hamiltonian’s expectation value over a simplified quantum circuit through Clifford transformations, reducing qubit and gate count. Illustrated with magnesium corrosion by water, this workflow advances DFT-based calculations, offering valuable applications for studying reactions like water adsorption on metal surfaces on near-term quantum computers.

**Figure 9:**
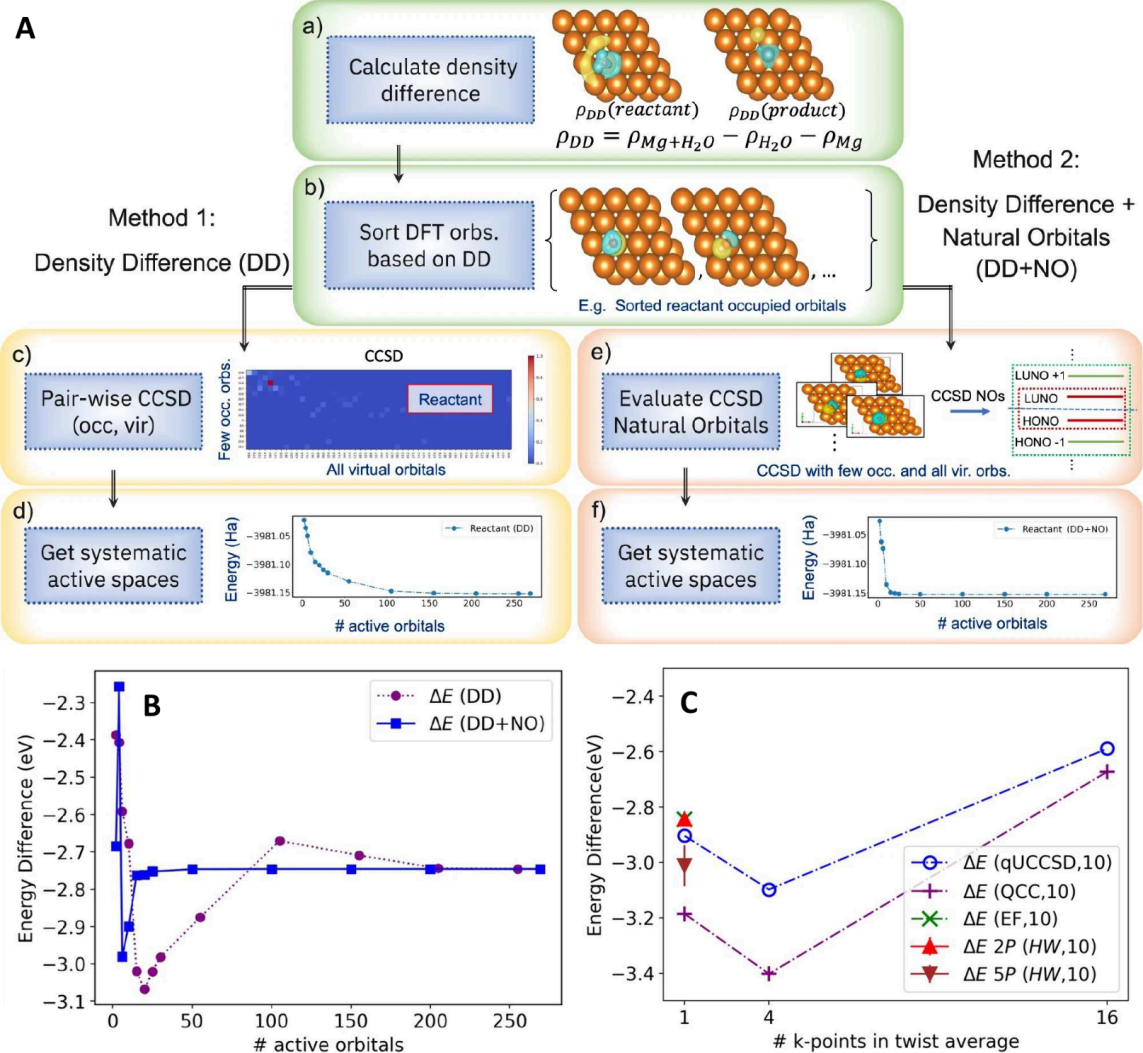
H_2_O dissociation on Mg(001). Panel (A): Common steps (a,b) shared by both methods, with left blocks (c,d) illustrating density difference (DD) method steps, and right blocks (e,f) depicting density difference + natural orbitals (DD+NO) method steps. Panel (B): Comparison of CCSD and energy differences Δ*E* calculated in active spaces constructed with DD and DD+NO methods. Panel (C): Depiction of energy differences Δ*E* derived from both noiseless classical simulations and hardware experiments. In 10-orbital active spaces, QCC was implemented with 50 Pauli operators (purple crosses) on classical simulators and with 2 and 5 Pauli operators (Δ*E* = 2*P* and Δ*E* = 5*P*) on quantum hardware. Reused from ref (260) under Creative Commons CC-BY 4.0. Copyright 2023 The Author(s).

The study begins with classical preprocessing and employs simple PBC calculations at the Γ-point for a time-reversal-symmetric Hamiltonian. To enhance convergence, twist-averaged boundary conditions (TABC)^293^ are applied. Main highlight of this work is the proposal of two strategies for constructing active spaces (Figure 9A), both initiating with the localization of occupied and virtual DFT orbitals projected onto an active region encompassing molecules involved in the reaction and a small surface portion. *Method 1*, known as the density difference (DD) approach, ranks occupied DFT orbitals based on their contribution to the electronic density difference, resulting in monotonically decreasing ground-state energies with increasing active-space size. However, this method exhibits slow convergence with increasing active-space size. *Method 2*, termed the density difference and natural orbitals (DD+NO) method, incorporates a coupled-cluster singles and doubles (CCSD) calculation in the active space, utilizing the five highest-ranking occupied DFT orbitals and all virtual orbitals. The inclusion of natural orbitals, sorted by decreasing occupation number, provides a systematic approach to defining active spaces in systems with strong correlation. The comparison reveals that DD+NO achieves faster convergence (Figure 9B), typically requiring only 15–20 natural orbitals as opposed to around 200 natural orbital with DD, but comes at a higher computational cost compared to DD. Both methods can be used complementarily to enhance the efficiency and accuracy of quantum chemical simulations.

Hardware simulations reveal a statistically consistent performance with noiseless classical simulations utilizing the same quantum circuit (Figure 9C). The study explores the efficacy of the VQE algorithm through Trotterized implementations of unitary CCSD (qUCCSD),^294^ entanglement forging (EF),^295^ and qubit coupled cluster (QCC).^296^ The calculated energy differences with QCC display a nontrivial dependence on the number of Pauli operators in the ansatz, notably with 50 Pauli operators deviating from qUCCSD by approximately 0.1–0.3 eV. The incorporation of EF results at the Γ-point facilitates effective handling of (2e,2o) (Here, e and o represents electrons and orbitals.) and (10e,10o) active spaces with 2 and 10 qubits, respectively, delivering results in good alignment with qUCCSD and QCC. It should be noted that the current implementation is restricted to Hamiltonians with time-reversal symmetry.

#### 6.4.2 O_2_ Dissociation on Pt(111): Quantinuum–BMW

**Figure 10:**
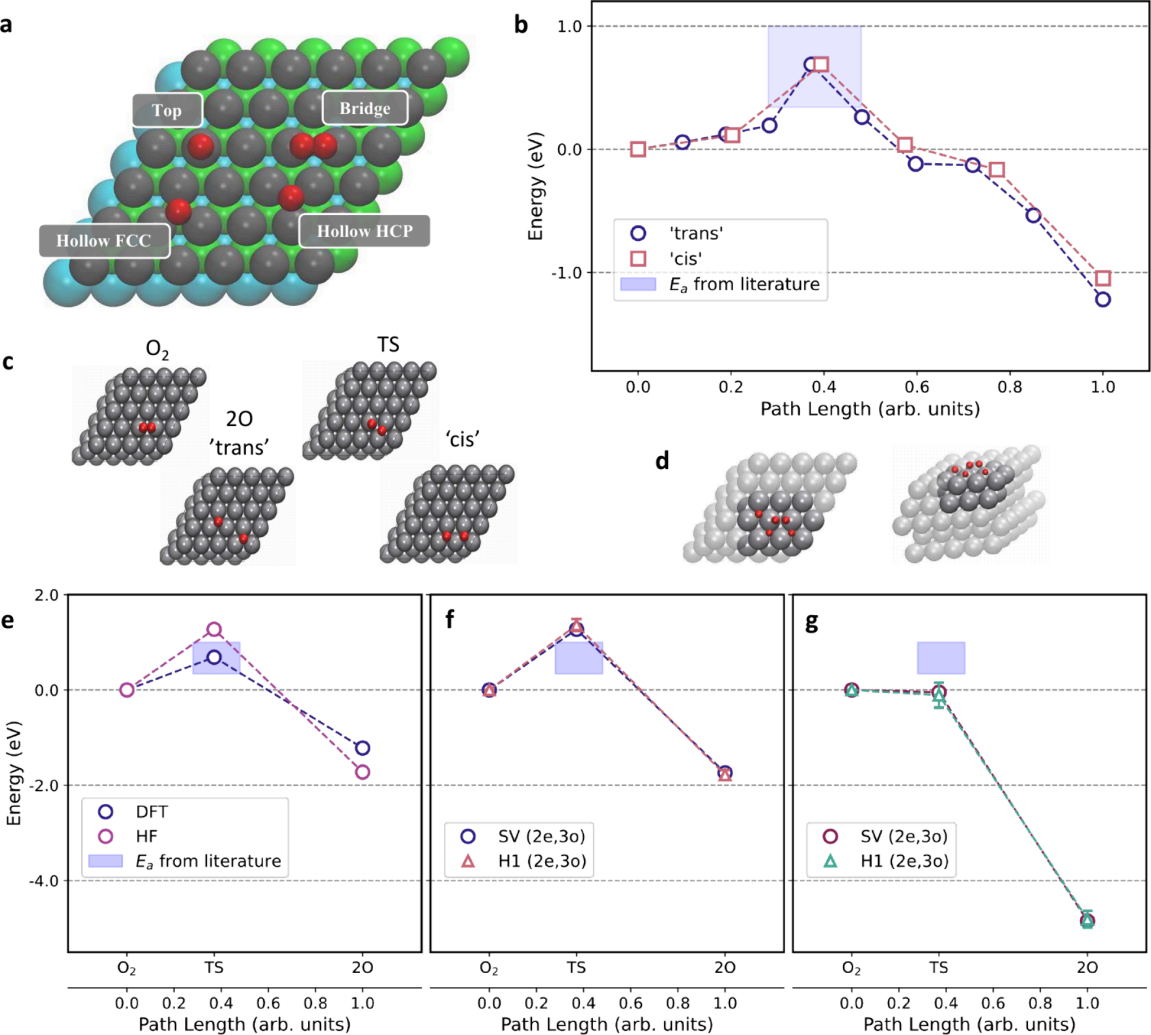
O_2_ dissociation on Pt(111). Panel (a) shows different high symmetry sites on a Pt(111) surface. Panel (b): Energy profiles calculated using nudged-elastic band (NEB) method connecting the initial adsorbed state (O_2_) to the final dissociated state (2 O) on Pt(111) surface for the “cis” (purple squares) and “trans” (violet circles). Activation energy *E*_a_ taken from literature is shown as a violet patch. Panel (c): Atomistic DFT models illustrate the reference initial O_2_, *O_2_-rotated TS, and 2O-“cis”/-“trans” states adsorbed on a Pt(5 × 5 × 5) slab, each associated with a path length value (0.00, 0.4, and 1.00, respectively) in panel (b). Panel (d): The Pt_19_O_2_ fragment using solid spheres, emphasizing potential oxygen occupation sites. In Panels (e,f), a comparison of adsorption (*E*_ads_), activation (*E*_a_), and dissociation energies (*E*_d_) calculated using various methods with respect to the initial state (O_2_ adsorbed) is presented. Panel (e) shows results from DFT and mean-field HF level calculations; panel (f) shows VQE calculations on the statevector simulator (SV) and Quantinuum “H1–1” device (H1), and panel (g) showcases VQE+NEVPT2 results on the same devices. Figure used with permission from ref (261). Copyright 2024 The Author(s).

The ORR on Pt and Co@Pt surfaces, was studied using the ADAPT-VQE algorithm^270^ on the H1–1 trapped-ion quantum computer. Static correlation exploration involved a complete active space approach on quantum hardware, while dynamic correlation was addressed with second-order perturbation theory (QRDM NEVPT2).^297^ The selection of the active space utilized the automatic regional embedding variant of the automatic valence active space (AVAS/RE) method.^298,299^ High-symmetry adsorption sites on the Pt and Co@Pt surfaces, the potential energy profile for O_2_ dissociation to dissociatively chemisorbed O atoms in cis- and trans- configurations and the initial, transition state and final configurations are displayed in Figure 10 (a), (b) and (c). For the 3-layer atomistic model, the AVAS/RE active space incorporated valence and higher orbitals of the ‘Pt_19_O_2_’ fragment Figure 10 (c). In the Co@Pt system, a model comprising 29 atoms was constructed, guided by density change analysis, traditional CAS notations, and the assurance that AVAS active orbitals were localized on O and the nearest Pt atoms. A smaller Hamiltonian was then formed with a (2e,3o) active space, compatible with hardware. The initial state preparation involved the use of ADAPT-VQE and (k = 1)-UpCCGSD. Reference converged DFT calculations demonstrated accurate results for the pure Pt catalyst with a small active space, while the Pt/Co catalyst required a larger active space to capture correlation energy, confirming strong correlation in the magnetic core–shell system.

DFT calculations indicated a halving of the barrier and a doubling of the driving force (Figure 10 (e)). The analysis of results, particularly in classical VQE statevector (SV) simulations (Figure 10 (f)), revealed a low correlation energy at the adsorption site. Simulations on H1 quantum computer aligned well for reactant (R) and product(P) states, while results for the TS state suggested an overestimation of *E*_a_ both with the SV and H1 simulations.

Challenges arose with the AVAS procedure due to finite magnetization in an restricted open-shell situation. The exclusion of half-filled Co 3*d* orbitals, contributing to total magnetism, was necessary to focus on correlations during O_2_ dissociation. Successful optimization of VQE state variational parameters on a classical CPU was achieved, but subsequent measurements of the active space Hamiltonian and spin-traced 1- and 2-RDM operators on both quantum hardware and the quantum noisy emulator presented complications.

#### 6.4.3 Battery Materials: Xanadu–Volkswagen

**Figure 11:**
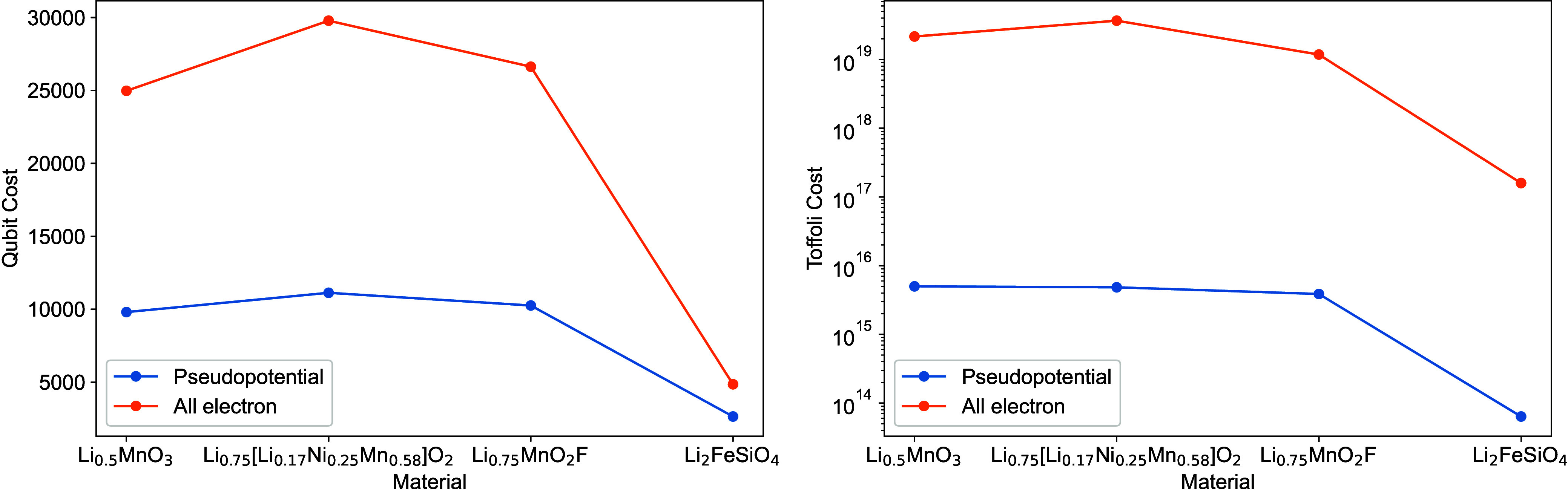
Li-excess battery materials resource estimation. Resource estimation for the pseudopotential (PP) and all-electron (AE) algorithms using η number of electrons in the supercell structural models and *N* number of plane waves required to converge the ground-state energy of the material at the level of density functional theory (refer to text for the exact numbers). Qubit cost is represented in terms of logical qubits. Plot made from data published in Table 2 of ref (259) under Creative Commons CC BY 4.0. Copyright 2023 The Author(s).

In classical simulations of materials, pseudopotentials are commonly employed to represent the effective potential arising from the nucleus and core electrons. A recent work introduces a quantum algorithm that leverages pseudopotentials to enhance the efficiency of simulating periodic materials on a quantum computer.^259^ The algorithm utilizes a qubitization-based QPE approach, employing a first-quantization representation of the Hamiltonian in a plane-wave basis. Addressing the challenge of pseudopotential complexity in quantum simulations, optimized compilation strategies for qubitization^300−304^ are developed. The computational cost of applying the algorithm to simulate lithium-excess cathode materials for batteries is estimated, including the required number of qubits and Toffoli gates for accurate simulations (Figure 11). For the calculation of resources, η values are 408 (808), 468 (968), 428 (836), and 100 (150), and *N* values are 5,473 (5.8 × 10^8^), 67,767 (8.7 × 10^8^), 57,655 (6.4 × 10^8^), and 19,549 (5.46 × 10^7^) for Li_0_·5MnO_3_, Li_0_·75[Li_0_·17Ni_0_·25Mn_0_·58]O_2_, Li_0_·75MnO_2_F, and Li_2_FeSiO_4_, respectively. The numbers outside the parentheses refer to pseudopotential, and numbers within the parentheses correspond to the all-electron implementation for both η and *N*. The optimized compilation strategies result in a pseudopotential-based quantum algorithm with a Toffoli cost 4 orders of magnitude lower than the previous state-of-the-art method, maintaining fixed target accuracy. They develop quantum read-only memories as a key component, minimizing complex arithmetic operations on a quantum computer and facilitating trade-offs between qubit and gate numbers. Overall, the quantum algorithm’s cost is reduced by about 4 orders of magnitude compared to the all-electron approach when applied to simulating lithium-excess materials (Figure 11). However, realizing the full potential of quantum computing necessitates ongoing efforts to further reduce algorithmic costs, addressing aspects such as the quality of initial state preparation methods,^62^ particularly for states with poor overlap, where repeated rounds of QPE may be required.

#### 6.4.4 Transition Metal Oxides: Riverlane–Johnson Matthey

While VQE possesses merits in specific scenarios, the prevailing consensus suggests superior scaling with system size for QPE.^81^ Therefore, in a recent study, the efficiency of QPE in estimating the ground-state energy of crystalline solids on error-corrected quantum computers is explored.^258^ The two most widely used basis sets, namely Bloch and Wannier representations,^305^ were employed in the context of qubitized QPE.^300−304^ Employing the sparse qubitization approach, the research estimates the resources required for calculating the ground-state energy of crystals with a supercell of approximately 50–70 atoms. The estimated number of T gates ranges from 10^10^ to 10^12^ when considering a basis set of 300–500 spatial orbitals. However, for realistic solids demanding at least double-ζ polarized (DZP) or triple-ζ polarized (TZP) basis sets, the T-gate count would be higher. To enable simulations of solids with larger basis sets on error-corrected quantum computers, an alternative approach involves selecting an active space within a few hundred orbitals or utilizing quantum embedding methods was suggested (Section 7). While this paper focuses solely on the single-shot cost of the total QPE circuit, it is noted that the effectiveness of QPE in estimating ground-state energy depends on the overlap between the initial state (e.g., Hartree–Fock state) and the true ground-state wave function,^62^ although this aspect was not explored in this paper.

**Figure 12:**
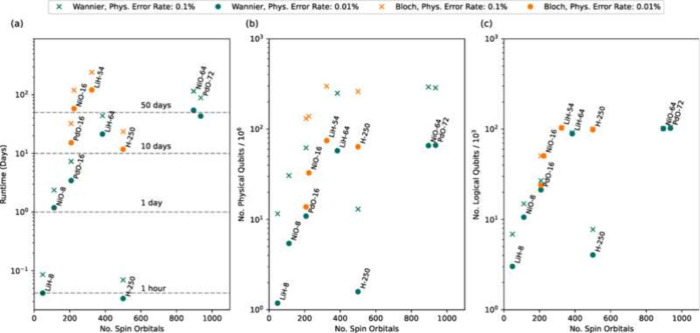
NiO and PdO resource estimation. Resource estimation for the calculation of ground-state energy in various solid-state systems employing Wannier and Bloch functions is provided. The estimations are based on L2-norm truncation, and the Hamiltonian simulations maintain an accuracy of 50 meV/f.u. (1.8 mHa/f.u.). The cycle duration of the code is 10^–6^ s, and the physical error rates are assumed to be 0.1% and 0.01%. The figures illustrate: (a) runtime in days, (b) number of physical qubits, and (c) number of logical qubits. The *x*-axis denotes the total number of spin orbitals. The numerical value alongside the crystal name indicates the number of atoms in the supercell and the size of the *k*-point mesh used for Wannier and Bloch basis sets, respectively. Reused from ref (258) under Creative Commons CC BY 4.0. Copyright 2023 The Authors.

Figure 12(a) shows the runtime for a single shot of the QPE circuit with a 50 meV/f.u. permissible error. It indicates that small-unit-cell simulations of NiO and PdO (8 and 16 atoms, respectively) take under 10 days, while larger computational cells like LiH (64 atoms) require about 50 days, even with a 0.1% physical error rate. NiO (64 atoms) and PdO (72 atoms) in supercells need approximately 100 days at a 0.1% physical error rate. A 10-fold reduction in the physical error rate to 0.01% roughly halves the runtime for all systems. Figure 12(b) and Figure 12(c) display physical and logical qubit counts. The smallest simulations need a few million physical qubits at a 0.01% error rate, while the largest simulations of NiO and PdO require around 65 million physical qubits. For a 0.1% error rate, quantum error correction raises the required number of physical qubits 4–5 times. Figure 12(c) indicates that small-cell simulations need a few thousand logical qubits, while large supercells require around 10^5^ logical qubits.

## 7 Embedding Approaches

So far we have explored the potential of quantum computers in simulating molecular and material properties, highlighting emerging algorithms for both NISQ and fault-tolerant systems with examples from academia and industry. However, current quantum computers are limited to performing *ab initio* calculations on only a few states due to qubit constraints. To tackle complex chemistry and material science problems with NISQ computers, it is crucial to reduce the number of electrons treated explicitly with high accuracy. We already saw an example of the speed-ups that can be obtained when introducing a pseudopotential approach to treat the chemically inert core electrons. In a similar fashion, one may separate a complex system into a part that is relevant for the property of interest and an environment that can be treated with a more approximate method or be neglected. In the context of quantum computing one may think of a hybrid approach, in which most of the calculation is done with a classical computer, letting the quantum computer tackle only that part of the problem that can not be described well by classical algorithms. To successfully utilize near-term quantum computers for larger systems, such hybrid quantum–classical methods are necessary, focusing on quantum computation only for specific parts of the system. This is particularly relevant for molecules and solids, where the required precision in the active region is much higher than that of the surrounding (bath) region. Many embedding theories have been proposed to address this challenge, as discussed in previous sections Section 5. Before we delve into the details of the individual techniques and their applicability to heterogeneous catalysis, we explain the general idea of embedding here.

**Figure 13:**
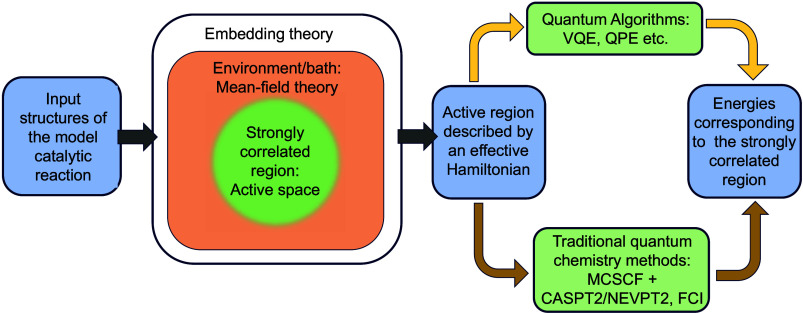
The general idea for the embedding approaches. Embedding involves dividing the entire system into two parts: the strongly correlated region (active space) and its surrounding environment or bath. The active space includes electronic states described by an effective Hamiltonian, solvable through traditional quantum chemistry methods such as multiconfigurational self-consistent field (MCSCF) + complete active space second-order perturbation theory (CASPT2)/*n*-electron valence-state second-order perturbation theory (NEVPT2), or full configuration interaction (FCI), represented by the dark yellow arrows. Quantum algorithms, such as quantum phase estimation (QPE) (Section 5.2.1) and variational quantum eigensolver (VQE) (Section 5.1.1), represented by light yellow arrows, can also be employed to calculate the energies of the strongly correlated regions.

Embedding is a powerful technique for studying complex chemical systems, particularly those involving large molecules (inorganic complexes) or extended systems (heterogeneous catalysis).^64^ The central theme of embedding involves partitioning the full system into an active region, treated using highly accurate quantum mechanics or quantum algorithms, and an environment region, treated using low-accuracy methods Figure 13. An embedding potential is used to account for the effects of the surrounding environment on the subsystems. An embedding Hamiltonian describes the interaction between the active region and the environment, facilitating the transfer of information and correlation between the two regions. This approach significantly reduces computational costs compared to a full quantum mechanical treatment while maintaining high accuracy. Numerous embedding techniques have been developed for quantum computing and periodic systems. In the upcoming discussion, we will explore these techniques, some of which have been mentioned in preceding sections, providing detailed insights into each method.

### 7.1 Embedding Using Localized Molecular Orbitals (LMO)

Molecular orbitals (MOs) play a crucial role in understanding chemical concepts and properties. Mean-field calculations such as Hartree–Fock (HF) or Kohn–Sham density functional theory (KS-DFT) yield valence orbitals with a well-defined energy, which is useful when studying electronic excitations and spectroscopy. A drawback for extended systems is, however, the spatial delocalization that MOs typically exhibit. Spatially localized MOs can be constructed to provide a better understanding of chemical bonding and photochemistry of the system.^306^ Such localized MOs (LMOs) are particularly important in local correlation treatments within post-HF methods. These LMOs serve as excellent starting orbitals for multiconfiguration calculations, such as state-averaged complete active space self-consistent field (SA-CASSCF),^307,308^ as well as for quantum calculations like state-averaged orbital-optimzied variational quantum eigensolver (SA-OO-VQE).^241,242^ Various schemes exist for generating localized orbitals, and for a comprehensive list we refer to the introduction section of earlier reviews.^306,309^ One notable scheme is the introduction of intrinsic atomic and bonding orbitals by Knizia,^310^ initially for occupied MOs and later extended to molecular fragments and relativistic spinors^311^ as well as to provide additional localized virtuals for correlation and use in time-dependent DFT.^312^ The procedure comprises two steps, definition of intrinsic fragment orbitals, followed by localization of the occupied and virtual subspaces by a generalization of the Pipek-Mezey localization algorithm.^313^ This scheme has been implemented in a standalone program known as the Reduction of Orbital Space Extent (ROSE), which has been interfaced with various quantum chemistry packages^311^ and was demonstrated by application in systems such as benzene, acrylic acid, ferrocene, Ir_*ppy*_3, microsolvated astatine anion, and tellurazol oxide complexes.^311^ While LMOs do not have a well-defined energy, they can be recanonicalized within each fragment to make them better suited for embedding purposes. Such a set of recanonicalized orbitals was recently used for calculating charge transfer states in chlorophyll dimer^314,315^ within the linear response framework of time-dependent density functional theory (TDDFT).^312^ By considering molecular fragments instead of isolated atoms, the convergence of the localization procedure can be improved, which is particularly valuable for embedding techniques such as the automated valence active space method.^298^ In summary, the use of LMOs yields a simple approach to define a reduced size local Hamiltonian that is compatible with many electronic structure methods, including the quantum algorithms discussed in this review.

In the following paragraph, the procedure followed in the construction of recanonicalized molecular orbitals is summarized. The process of embedding using LMOs begins with a supersystem HF calculation, which yields a set of canonical molecular orbitals (CMOs) for the supersystem. Subsequently, these CMOs are explicitly localized within each subsystem (I). This localization procedure aligns with the concept of intrinsic atomic and bonding orbitals, as introduced by Knizia (referred to as IAOs and IBOs), which has been extended to molecular fragments in ROSE. In this approach, the localization is carried out within a minimal basis of intrinsic fragment orbitals (IFOs), and the reference orbitals are defined by fragment MOs (RFOs) acquired through separate HF SCF calculations for each subsystem. For the details of the construction of the IFOs and the recanonicalized LMOs we refer the reader to references.^311,312^ Briefly, the steps for the construction of the recanonicalized LMOs can be summarized as

**Figure 14:**
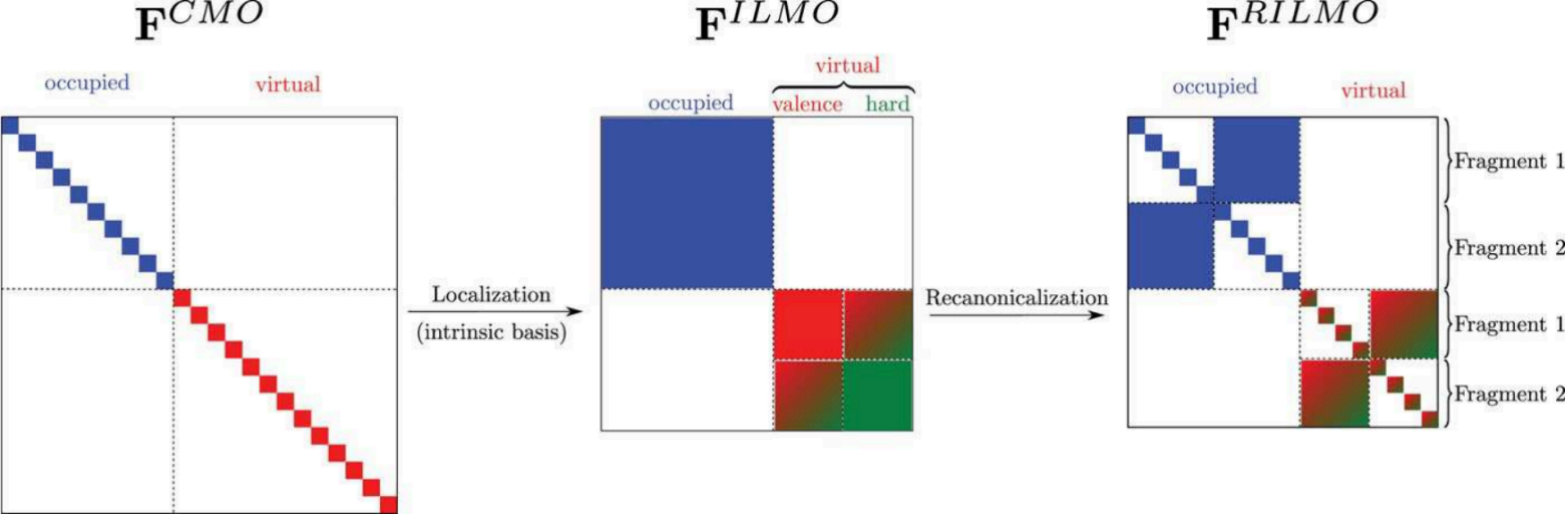
From canonical molecular orbitals (CMOs) to recanonicalized intrinsic localized molecular orbitals (RILMOs). Illustration of the Fock matrix in various bases with color-coded blocks denoting nonzero matrix elements, while smaller squares depict individual matrix elements. Reused from ref (312) under Creative Commons CC BY 4.0. Copyright 2023 The Author(s).

1. Construction of Intrinsic Fragment Orbitals (IFOs) using a predefined set of RFOs.
2. Separate localization of the occupied, valence virtual and hard virtual orbitals generating the so-called intrinsic LMOs (ILMOs).
3. Diagonalization of the Fock matrix in the ILMO basis insie each fragment generating our fianl recanonicalized intrinsic localized molecular orbitals (RILMOs) (Figure 14).

These RILMOs provide a foundation for detailed analyses and computations in the context of embedding and quantum chemistry. Additional technical details related to the LMO construction process can be explored in the literature for a more comprehensive understanding of the methodology. This procedure has been implemented in the Reduction of Orbital Space Extent (ROSE) code,^316^ a standalone code which is independent of the underlying electronic structure code used. This code can be used to generate LMOs that can be used as a starting point for any quantum chemistry calculation on quantum computers.

### 7.2 Dynamical Mean-Field Theory (DMFT)

Dynamic Mean Field Theory (DMFT) is a powerful theoretical framework in quantum chemistry and condensed matter physics that focuses on capturing the effects of strong electron–electron correlations in strongly correlated systems, such as transition metal compounds and high-temperature superconductors.^317−323^ DMFT, a variant of the popular mean field theory (MFT), takes into account the dynamics of the system, hence its name. DMFT achieves this by self-consistently embedding the Green’s function of local fragments within a fluctuating environment.

**Figure 15:**
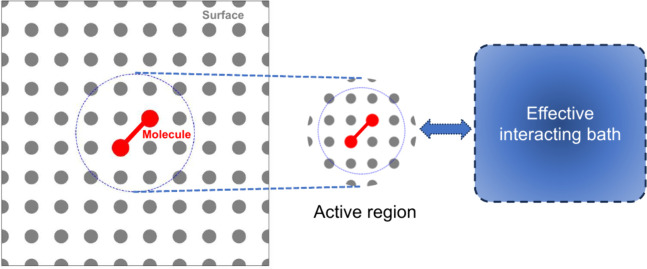
The idea of dynamical mean-field theory (DMFT). DMFT replaces the idea of a solid with a single atom exchanging electrons with a self-consistent medium by capturing local many-body correlations on each site. It describes a strongly correlated system by coupling a “local moment” on each lattice site to a “bath” of noninteracting electrons to effectively consider electron–electron interactions and correlations in the system. When DMFT is applied to modeling molecule–surface reactions, the strongly correlated region of interest, such as a localized molecular orbital or an adsorbate on a surface, effectively interacts with an effective bath formed by the rest of the system. The left figure shows the active region on a surface (gray circles) with an adsorbed molecule (red circles with a bond). On the right, the strongly correlated region marked with a blue dotted circle interacting with an effective bath formed by the rest of the system (blue patch) is shown.

One of DMFT’s significant contributions is its ability to extend quantum chemical methods, originally designed for finite systems, to tackle infinite periodic problems while employing a local correlation approximation. This locality of correlation suggests that the daunting computational scaling with Brillouin zone (BZ) size can be circumvented, as DMFT effectively operates as a self-consistent theory for a single unit cell within a crystal lattice.^324^ For example, consider a crystal lattice composed of unit cells, where one unit cell is embedded in the surrounding medium constituted by the rest of the crystal. This arrangement, dictated by translational symmetry, necessitates a self-consistent embedding theory. While an exact embedding calculation would be as computationally intensive as a full crystal calculation, DMFT offers a solution. By neglecting intercell correlations based on their localized nature, DMFT efficiently addresses the computational challenges associated with the scaling of the Brillouin zone size. This approximation enables DMFT to treat the crystal effectively, considering it as a self-consistent theory focused on a single unit cell. Effectively, DMFT replaces the description of a solid with a simplified model where each lattice site is coupled to a self-consistent medium. This medium captures local many-body correlations on each site, effectively treating the system as a collection of single atoms interacting with this medium. This approach allows DMFT to capture electron–electron interactions and correlations in strongly correlated systems. This idea can be extended to the application of DMFT to modeling molecule–surface reactions (Figure 15) relevant to heterogeneous catalysis. In this context, the strongly correlated region of interest, such as a localized molecular orbital or an adsorbate on a surface, is effectively treated as the “local moment” within DMFT. This region interacts with an effective bath formed by the rest of the system, which accounts for the nonlocal correlations and interactions. This description is consistent with the general framework of DMFT, where the local properties of the strongly correlated region are coupled to an effective environment.

DMFT’s formulation revolves around Green’s functions and is structured as a self-consistent theory for the Green’s function of a unit cell, which could be a primitive cell or a computational supercell. Notably, the local correlation approximation in DMFT assumes that the self-energy is local, implying that intercell elements of the self-energy vanish or, in momentum space, that the self-energy is momentum-independent. It is worth emphasizing that while DMFT considers correlation effects between unit cells through the embedding method (e.g., DMFT+LDA will have LDA correlations between unit cells; LDA - local density approximation) and accounts for one-electron delocalization effects between them. This, coupled with DMFT’s self-consistent nature, sets it apart from simpler quantum chemical embedding formalisms that incorporate quantum mechanical clusters into a medium described by molecular mechanics.

In DMFT, the self-consistency condition is a crucial concept that ensures the consistency between local and nonlocal properties of the system. The local subset of degrees of freedom, often referred to as the active space, usually consists of localized orbitals or lattice sites where strong electronic correlations are present. The Green’s function (a mathematical representation of the correlation between particles in a quantum system) for this active space is denoted as *G*_loc_(ω). On the other hand, the total system’s Green’s function, denoted as *G*(**k**, ω) includes contributions from all momentum (**k**) points in the Brillouin zone. The self-consistency condition in DMFT demands that the local Green’s function *G*_loc_(ω) should be equivalent to the average of the total system’s Green’s function *G*(**k**, ω) over all momentum points (**k**). In mathematical terms:
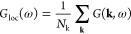
8where *N*_k_ is the total number of momentum points. Physically, this condition implies that the local properties of the system (captured by *G*_loc_(ω)) should be representative of the average behavior of the system across all momentum points. This is justified because the higher order correlation effects are dominantly local.^324^ Achieving this equivalence typically involves an iterative procedure. One starts with an initial guess for *G*_loc_(ω) and computes the total system’s Green’s function *G*(**k**, ω) using this guess. Then, one updates the local Green’s function based on the average of *G*(**k**, ω) over all momentum points. This process is repeated until convergence is achieved, i.e., until *G*_loc_(ω) and *G*(**k**, ω) are consistent with each other. In summary, the self-consistency condition ensures that the local properties captured by are consistent with the behavior of the system across all momentum (**k**) points, thus providing a reliable description of strongly correlated electron systems within the DMFT framework.

In the quantum computing context, a hybrid approach was proposed to be used on a quantum computer that integrates classical and quantum algorithms into the DFT + DMFT embedding framework.^325^ Within this scheme, a cost-effective DFT calculation is employed to establish a set of orbitals and ascertain the electronic structure for the majority of these orbitals. Simultaneously, a more computationally intensive many-body method is applied to solve a reduced model comprising a significantly smaller set of correlated orbitals. In a recent study, researchers proposed an alternative approach that leverages the VQE method for ground and excited states in the context of an exact diagonalization.^326^ The algorithm is specifically designed for a two-site DMFT system, addressing the single-band Hubbard model on the Bethe lattice with infinite connectivity using exact diagonalization of a two-site impurity problem comprising one interacting and one bath site. Through comprehensive benchmarks conducted on superconducting and trapped ion qubits for the 2-site DMFT model, it was demonstrated that practical calculations with minimal error are feasible. Overall, this proof-of-concept demonstration showcases the viability of running DMFT calculations on contemporary quantum hardware. Furthermore, utilizing the quantum circuit simulator Qulacs,^327^ the algorithm was validated by computing Green’s functions for various impurity models, including the dimer and four-site impurity models derived from DMFT.^328^ The results, including the imaginary-time Green’s function and Matsubara Green’s function, exhibited very good agreement with exact solutions. Additionally, an efficient computation of the imaginary-time Green’s function was achieved by employing a nonuniform mesh, while addressing numerical instabilities through adaptive mesh generation and energy convergence conditions. It is essential to note that while these applications have demonstrated success with model systems, their adaptation to realistic heterogeneous catalytic systems remains unexplored and will require further studies on scaling and accuracy.

In summary, DMFT offers a versatile framework for tackling strong electron–electron correlations in materials, enabling the extension of quantum chemical methods from finite systems to infinite crystals while circumventing the computational burden associated with large Brillouin zones. It achieves this through a local correlation approximation and self-consistent embedding, making it a valuable tool in the study of correlated electron systems in heterogeneous catalysis.

### 7.3 Quantum Defect Embedding Theory (QDET)

A quantum defect embedding theory (QDET) for calculating strongly correlated electronic states of active regions using a highly accurate method, while using random phase approximation (RPA) to describe the rest of the system was proposed by Galli and co-workers.^329^ QDET draws heavy inspiration from the constrained random phase approximation (cRPA) method.^330^ In cRPA, the active space typically consists of a subset of electrons or degrees of freedom that are strongly correlated and of interest for the particular system being studied. RPA approximates the polarization by a summation of all particle-hole excitations in the system. In cRPA, all the particle-hole excitations except those within the active space are considered. This polarizibility is then used to effective parametrize the interactions within the active space. By imposing these constraints, cRPA provides a systematic way to incorporate nonlocal correlations from the environment into the description of the active space, leading to improved accuracy in the treatment of strongly correlated systems. Modified and improved implementations of cRPA have been used for calculating electronic excitations on large-scale simulations of nitrogen-vacancy states in a periodic hBN monolayer and hBN-graphene heterostructure,^331^ electronic states of twisted bilayer graphene (tBLG) characterized by giant unit cells and correlated electronic states,^332^ and optical excitations in the negatively charged nitrogen vacancy (NV) center defect in diamond.^262^

In cRPA calculations, two common approximations are typically made to evaluate dielectric screening: first, the adoption of the random phase approximation (RPA) to the screened Coulomb interaction, which only approximately captures exchange-correlation effects between electrons and may lead to inaccuracies; and second, the Adler-Wiser formalism, which involves explicit summations over empty states, potentially hampering computational efficiency. In contrast, QDET addresses both of these approximations. It goes beyond the RPA by computing dielectric screening with inclusion of exchange-correlation effects, which are evaluated using a finite-field algorithm.^333−335^ Moreover, QDET circumvents the need for explicit summations over empty states by employing a compact basis derived from the spectral decomposition of density response functions.^336−339^ This approach enhances both the accuracy and efficiency of calculations. The methodology behind QDET, detailed elsewhere^65,340^ offers scalability advantages, particularly for materials containing thousands of electrons, as it does not necessitate the explicit evaluation of virtual electronic states.^336,339^ The stepwise strategy used for QDET is shown below.

1. Perform spin-restricted DFT calculation on the entire system using hybrid functionals
2. Selection of active space: choose single-particle defect wave function; include relevant resonant and band-edge states; verify the choice of active space size - converged excitation energies
3. Construct effective Hamiltonians including exchange correlation effects
4. Obtain many-body ground and excited state using quantum algorithms (QPE and VQE) and compare with classical FCI calculations, when available

**Figure 16:**
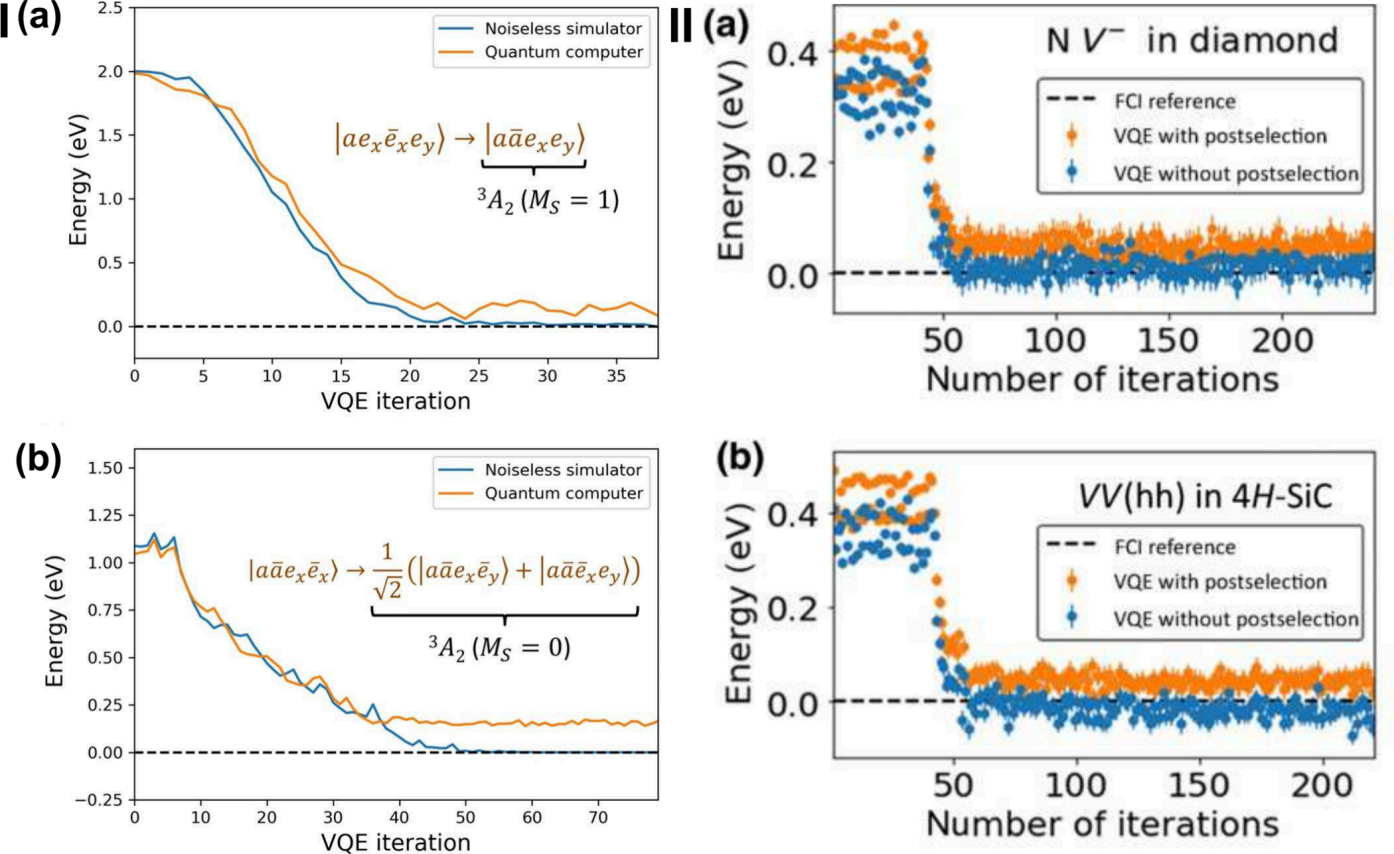
(I) (a) VQE estimation of the ground-state energy of NV center diamond starting from *M*_*S*_ = 1 state. (b) The VQE estimation of the ground-state energy NV center diamond starting from a *M*_*S*_ = 0 state results in a strongly correlated state with an error of 0.2 eV. (II) VQE optimization of the ground-state energy for (a) the NV^–^ center in diamond and (b) VV in 4H-SiC. The optimization is conducted using the VQE algorithm with four and six qubits, respectively, on *ibmq*_*casablanca*. The orange dots represent the results with postselection of states, while the blue dots depict results without postselection. FCI energy is provided for reference. Figure reused from ref (329) [Copyright 2020 The Author(s)] and ref (237) (Copyright 2022 The Authors) under Creative commons CC BY 4.0.

Using QDET, the ground and excited-state properties of spin-defects, encompassing the nitrogen vacancy (NV) center in diamond, silicon vacancy (SiV) in diamond, and Cr impurity 4^+^ in 4H-SiC were calculated. Full Configuration Interaction (FCI) simulations for NV center diamond reveal the correct symmetry and ordering of low-lying electronic states. SiV diamond exhibits similar values in active space bands with or without exchange-correlation effects. For the hexagonal configuration of Cr-4H-SiC, QDET’s effective Hamiltonians enable the investigation of electron–electron spin flip transitions for the half-filled level. While FCI calculations were performed for all three systems, quantum simulations were conducted only on the NV center diamond using QPE and VQE algorithms on 6 qubits, representing 4 electrons in 3 orbitals, with UCCSD ansätze. Using a simulator, convergence was demonstrated for the exact ground-state energy. QPE simulations on a simulator show good agreement with FCI, with increasing auxiliary qubits converging to FCI. QPE calculations were not performed on a quantum hardware. In VQE calculations, the active space size was further reduced, and correlated and uncorrelated states with 4 qubits were computed on a simulator and an actual quantum computer (IBMQ Yorktown). While VQE on simulators converged to FCI energies for both uncorrelated and correlated states, only the uncorrelated calculations on the hardware converged (Figure 16.I). The quantum hardware results display a 0.2 eV error for the uncorrelated state.

In an another study, electronic structure calculations were performed on strongly correlated ground and excited states of the N–V^–^ center in diamond and the VV in 4H SiC, both of which are point defects in semiconductors.^237^ The calculations utilized a combination of DFT and QDET. The ground states were computed using VQE, while the excited states employed QSE. Notably, these calculations were executed on quantum hardware. As some results fell below FCI energies, which is deemed unphysical, a postselection method based on partial constraints on the number of electrons was successfully applied. Within their postselection method, all measurements that do not conserve the number of electrons are discarded. This postselection method ensured physically meaningful and converged results during the VQE calculation (Figure 16.II). Further, to address noise issues, an error mitigation technique within the Zero Noise Extrapolation (ZNE) scheme,^341^ utilizing an exponential block to enhance the control over quantum errors in Unitary Coupled Cluster (UCC) type ansätze was introduced. The authors assert the method’s applicability without prior knowledge of the hardware noise source, all without increasing the number of qubits.

In the original formulation of QDET presented in ref (329), the authors adopted an approximate double counting correction based on Hartree–Fock theory. In a recent work, a more rigorous derivation of QDET is presented based on Green’s functions, and an exact double counting correction is derived^342^ which is similar to what was was already known in the community.^330^ This correction is exact within the *G*_0_*W*_0_ approximation (The *G*_0_*W*_0_ approximation is a commonly employed technique, where the self-energy is formulated as the convolution of a noninteracting Green’s function (*G*_0_) and a screened Coulomb interaction (*W*_0_) in the frequency domain. This also holds true for the *GW* self-energy beyond *G*_0_*W*_0_. The main feature of *G*_0_*W*_0_ is that the off-diagonal elements of the self-energy are neglected and the KS orbital energies are therefore corrected perturbatively.) and when retardation effects are neglected.^343^ The authors refer to this correction as EDC@*G*_0_*W*_0_ (exact double counting at the *G*_0_*W*_0_ level of theory). Furthermore, quantum embedding theories were compiled in a recent review on embedding theories designed for electronic structure calculations of solids on noisy intermediate-scale quantum computers.^344^ Specifically, the focus is on a class of materials, solid materials housing spin defects, with examples highlighting their application. However, it is important to note that embedding schemes, including QDET, demonstrate potential versatility by being applicable to diverse localized, highly correlated states. This includes states found in solvated ions, nanostructures, surface adsorbates, as well as catalytic sites at surfaces and interfaces that are extremely relevant to heterogeneous catalysis.

### 7.4 Density Matrix Embedding Theory (DMET)

Density Matrix Embedding Theory (DMET) is another embedding theory that is used to simulate strongly correlated electronic systems.^345,346^ Like DMFT, DMET employs a strategy where a localized fragment, treated with high precision, is embedded within a surrounding environment treated with lower precision. This approach allows for a focused treatment of the important region while simplifying the representation of the entire system. The primary distinction between DMET and DMFT lies in their embedding strategies. DMET embeds the ground-state density matrix exclusively, eliminating the need for a frequency-dependent formulation. In contrast, DMFT embeds the Green’s function, resulting in a different approach to describing the system-environment interaction. The density matrix of the active region is then used to embed the active region into the environment region, thus correlating the two regions. This method allows for the treatment of strongly correlated systems, such as transition metal complexes, with high accuracy at a relatively low computational cost. Additionally, by treating the active region with an exact method and the environment region with a mean-field method, DMET can capture both short-range and long-range correlation effects in the system. It can also be used to simulate large and complex systems that are difficult to treat using traditional methods, such as systems pertaining to heterogeneous catalysis. For more in-depth exploration of this topic, readers are directed to a tutorial-level review on DMET.^347^

In the quantum computing context, DMET has been used to model carbon capture on metal–organic frameworks.^263^ DMET combined with the VQE and active space approach was used to study CO^2^ adsorption in Al-fumarate metal–organic frameworks (MOF), an important reaction in carbon capture.^263^ We note that the starting second quantized Hamiltonian was formed in a minimal STO-3G basis so results should not be compared directly to experimental observations. The quantum simulations were performed on noise-free and noisy emulator backends and error mitigation schemes were applied on the results. Four different fragmentation strategies were used to calculate CO^2^-MOF bond stretching energy, which for larger bond distances, r ≫ 2 Å corresponds to the bond dissociation energy. All four fragmentation strategies gave different results while one of the four schemes provided reasonable results for the bond dissociation energy. In an other case, simplified models of hydrogen chain and iron crystals were studied using DMET and VQE.^278^ This study is already discussed in Section 6.2. Given its usefulness, it has been integrated to be a part of the workflow of Inquanto,^348^ a software developed by Quantinuum that is capable of performing chemistry calculations using quantum algorithms. In addition to these algorithms, two new approaches have been proposed to leverage embedding techniques in near-term quantum computers. One approach is based on DMET,^349^ while the other is based on the projection-based embedding method.^350^ However, the details of these algorithms will not be discussed here.

### 7.5 Embedding Approaches: Summary and Outlook

Considering the size of the active space essential for accurately capturing strong correlation effects in catalytically relevant materials and the number of atoms required to define realistic models of heterogeneous catalysts, embedding approaches emerge as the most practical and promising strategy to make calculations feasible. The continuous advancement of quantum computing, marked by improvements in qubit quality and classical-quantum communication connectivity, opens up opportunities for embedding approaches that utilize both classical and quantum computing techniques. It is essential to recognize that increase in the number of qubits in a quantum processor must be accompanied by improvements in qubit fidelity to achieve meaningful progress. This realization has prompted discussions about quantum-centric supercomputing,^67,68^ a paradigm that emphasizes the unique strengths of quantum computers, particularly their suitability for specific problem types. The ongoing collaboration between the high-performance computing (HPC) and quantum computing communities holds great promise, showcasing a collective effort to harness the strengths of both paradigms.^351−353^ The development of tools for circuit cutting and knitting^354^ is particularly noteworthy, as it enables more seamless integration of embedding approaches into the broader computational landscape. Looking forward, as embedding approaches become more practical and viable in the near future, in line with previous observations,^68^ we can see a quantum-centric computing approach to materials modeling emerge. Such an approach can leverage the strengths of both classical and quantum computing, offering a synergistic solution to the challenges faced in heterogeneous catalysis modeling.

## 8 Kinetics and Uncertainty Quantification

A final topic in which quantum computing may have an impact is in the modeling the entire set of reactions that can occur in a certain heterogeneous catalytic process. Understanding these kinetics and quantifying uncertainties that result from incomplete knowledge of the reaction constants or even mechanisms are vital for optimizing catalytic processes. Furthermore, this step serves as a connection between the atomistic scale to the reactor scale processes. Kinetic models, particularly microkinetic models, serve thereby as valuable tools for elucidating reaction mechanisms and predicting reaction rates. One notable advantage of microkinetic models is their ability to describe reactions because they can be cast as linear systems of equations in a straightforward manner.

Recent advancements in quantum computing have shown promise for the application of the Harrow, Hassidim, and Lloyd (HHL) algorithm^76^ in the realm of kinetics and uncertainty quantification in heterogeneous catalysis. The HHL algorithm is a quantum algorithm specialized in numerically solving linear systems of equations (Section 5.2.2). Writing the kinetic equations as a system of linear equations, Walker and his co-workers have explored these areas.^73−75^ One of their works emphasizes the setup of a CO oxidation microkinetic model using a quantum circuit, emphasizing the advantage of microkinetic models that eliminate the need for an encoding step.^73^ In the HHL algorithm, the vector |*b*⟩ is encoded using additional qubits in a quantum register. Each element of the vector |*b*⟩ is represented by the state of these qubits. This encoding typically involves mapping the amplitudes of the vector onto the quantum states of the qubits. In their algorithm, Walker et al. utilize the steady state approximation and mass balance to represent the input vector |*b*⟩ with binary encoding, effectively eliminating the need for a separate encoding step. It demonstrates that the linearized set of equations can be solved with reasonable accuracy in a single iteration. In another paper, they present a method for uncertainty quantification using reduced microkinetic models and the logarithmic scaling of qubits, again, leveraging the HHL algorithm to solve linear systems.^74^ Comparisons with classical methods are made, and the potential for quantum advantage is highlighted, along with the challenges encountered when dealing with larger systems. Furthermore, in a more recent paper, they introduce a quantum circuit approach for modeling steady-state behavior in homogeneous hydrogen-air combustion.^75^ Empirical testing reveals critical factors influencing the accuracy of the HHL algorithm, providing valuable insights for the preconditioning of reduced models. These papers showcase the potential of quantum computing and the HHL algorithm in kinetics and uncertainty quantification, paving the way for further advances in heterogeneous catalysis research. That being said, the HHL algorithm, meant for solving linear systems of equations, is not ideal for NISQ computers because it needs high qubit connectivity, long coherence times, and low errors in gates and measurements. The challenges of implementing the HHL algorithm on NISQ computers and the conditions necessary for achieving speedup are discussed in Section 5.2.2.

## 9 Summary and Outlook

In summary, this review has explored the emerging research field of modeling heterogeneous catalysis through quantum computing algorithms, encompassing academic advancements, industry demands and collaborative efforts of academia and industry. The quest for active and selective catalysts, including emerging materials such as multicomponent alloys, single-atom catalysts, and magnetic catalysts, has underscored the limitations of conventional methods like DFT, particularly in capturing strong correlation effects and spin-related phenomena. Quantum computing has the potential to emerge as a transformative tool, as it is intrinsically better suited to overcome these challenges than conventional computational chemistry methods.

Within quantum computing algorithms, our primary focus has been on the variational quantum eigensolver (VQE), as this is the most extensively researched algorithm in the current noisy intermediate-scale quantum (NISQ) era. Considering the current landscape with only a few thousand qubits within reach, it is likely that VQE will remain a dominant tool for computational tasks in the near future. However, to look further ahead we also briefly discussed other algorithms such as quantum phase estimation (QPE), the Harrow–Hassidim–Lloyd (HHL) algorithm, and quantum singular value transformation (QSVT). These algorithms are poised to play an important role in the transition to the early fault tolerant quantum computing (EFTQC) era and the subsequent fault-tolerant quantum computing (FTQC) era, where the availability of a few hundred thousand error-corrected qubits could change the landscape of computational approaches for heterogeneous catalysis research.

In our exploration of quantum computing applications in heterogeneous catalysis, we underscored the preliminary but promising utility in industrial use cases. While current applications concern proof-of-principle studies with basis sets that are far too small to reach chemical accuracy or even rival classical computing approaches, they serve to explore what will be possible with more potent quantum computers. The many collaborative efforts between academia and industrial partners in researching these applications are illustrative for the rapidly increasing interest in this field.

As an example, we highlighted studies where VQE, initially designed for modeling molecular systems, was extended to address periodic systems. We furthermore looked at efforts to use VQE in calculating electronic band structures, a crucial component for studying photocatalysis applications. Additionally, we discussed use cases involving the computation of bulk lattice constants and molecule–surface reactions, where embedding approaches were employed to tackle challenges posed by the system’s size. We dedicated a section and delved into the details of embedding methods, drawing attention to a hybrid strategy where quantum computing algorithms handle the strongly correlated region, while reasonably accurate and cost-effective traditional quantum chemistry algorithms, like DFT, address the remainder of the system. Finally, we briefly touched upon uncertainty quantification in heterogeneous catalysis, which find a potential application in modeling catalysis at the reactor scale.

Looking ahead, the utilization of quantum computing in heterogeneous catalysis research with its complex and large to solve models has considerable potential to lead to break-through developments. This observation is corroborated by the investments by both academic and industrial players which signal a growing interest in exploring quantum computing in this context. This review emphasizes the importance of adapting computational methodologies for strongly correlated systems where quantum computing can provide an advantage over classical algorithms. If theory, algorithms, and hardware developments continue to progress, quantum computing may reach its potential and start to play an important role in modeling heterogeneous catalysis. Envisioning such a future where quantum algorithms seamlessly integrate into catalysis research workflows, we expect that the journey into quantum computing can help to push the boundaries of our understanding in heterogeneous catalysis.

## Glossary

Heterogeneous catalysis: Heterogeneous catalysis is a type of catalysis where the catalyst is present in a different phase (solid, liquid, or gas) from the reactants. The reactants adsorb onto the surface of the catalyst, undergo chemical reactions, and then desorb as products. Heterogeneous catalysis plays a crucial role in various industrial processes, such as petroleum refining, chemical synthesis, and pollution control.
Single-atom catalysts (SACs): SACs represent a novel class of catalysts consisting of isolated metal atoms dispersed on a support material. Their unique electronic and geometric structures enable exceptional catalytic activity and selectivity, offering promising avenues for sustainable chemical processes. SACs provide precise control over catalytic reactions because of their high surface area and tunable active sites, making them highly desirable for various industrial applications.
Multicomponent alloys: Multicomponent alloys serve as versatile catalysts in heterogeneous catalysis by offering tunable properties that can enhance catalytic performance. By combining multiple elements, these alloys can exhibit synergistic effects, which promotes desirable catalytic reactions while minimizing undesired byproducts. Their tailored composition enables precise control over surface reactivity and electronic structure, which is crucial for catalyzing complex chemical transformations.
Density functional theory (DFT): DFT is a computational method widely used in quantum chemistry and condensed matter physics to calculate the electronic structure and properties of molecules, solids, and surfaces. It is based on the concept that the total energy of a system can be determined by the electron density rather than the wave function. DFT employs the exchange-correlation functional to describe the electron–electron interactions, which makes it a computationally efficient method for large systems.
First quantization: First quantization is a formalism used to describe quantum systems by directly considering the wave function of individual particles or the system as a whole. It involves solving the Schrödinger equation for the wave function in terms of the coordinates and momenta of the particles. First quantization is commonly used in introductory quantum mechanics courses and provides a foundation for understanding the principles of quantum theory.
Second quantization: Second quantization is a mathematical framework used to describe quantum systems with multiple identical particles. It treats the particles as indistinguishable entities and represents the quantum state of the system in terms of creation and annihilation operators acting on a vacuum state. Second quantization is widely used in quantum field theory, many-body physics, and quantum chemistry to describe systems with variable numbers of particles.
Atom-centered basis set: An atom-centered basis set is a set of mathematical functions used to represent the electronic wave functions in molecules or molecular systems. These basis functions are centered on individual atoms and describe the spatial distribution of electrons around each atom. Basis sets can be composed of Gaussian functions or numerical grid representations and are crucial for accurate quantum chemical calculations of molecular properties.
Plane waves: Plane waves, in the context of computational materials science and electronic structure calculations, are a mathematical basis set used to represent the electronic wave functions in periodic systems. They are characterized by having a constant amplitude and a wavefront that is a plane perpendicular to the direction of propagation. Plane wave methods are commonly employed in solid-state physics and materials simulations.
Wave function: In quantum mechanics, a wave function represents the quantum state of a system. It is a mathematical function that describes the probability amplitudes of different possible outcomes when measuring observables of the system. The square of the wave function gives the probability density of finding the system in a particular state.
Hamiltonian: The Hamiltonian is an operator in quantum mechanics that represents the total energy of a system. It includes the kinetic energy and potential energy terms and is used to describe the time evolution of the wave function according to the Schrödinger equation. The Hamiltonian operator provides information about the observable properties and behavior of a quantum system.
Full configuration interaction (FCI): FCI is a quantum chemical method that provides an exact solution to the electronic Schrödinger equation within a given basis set. It involves considering all possible configurations of electron occupation in the molecular orbitals leading to an exact description of the electronic wave function. However, the computational cost of FCI scales exponentially with the system size, thereby limiting its application to small systems.
Active space: In quantum chemistry, the active space refers to the subset of molecular orbitals and electrons considered as the most relevant for describing the electronic structure of a system. It is typically chosen based on the specific chemical and physical properties under investigation. The active space is defined by selecting a specific number of occupied and virtual orbitals and the corresponding electrons.
Strong correlation: Strong correlation refers to situations in quantum systems where the standard mean-field methods, such as Hartree–Fock theory or density functional theory, fail to accurately describe the electronic structure due to strong electron–electron interactions. Strong correlation effects are prevalent in systems with open-shell configurations, transition metals, and molecules with significant electronic delocalization.
Static correlation: Static correlation refers to the correlation effects in a molecular system that arise because of the degeneracy or near-degeneracy of two or more electronic states. It is characterized by the mixing of electronic configurations with significantly different occupancies, leading to difficulties in describing the electronic structure with single-reference methods.
Dynamic correlation: Dynamic correlation refers to the correlation effects in a molecular system beyond the static correlation effects. It involves the multitude of weak correlations, like the dispersion interactions between different molecules, as well as interactions between the highly localized core electrons and the spatially more distributed valence electrons. Dynamic correlation effects are crucial for accurately describing the potential energy surfaces and reaction mechanisms.
Embedding: In the context of quantum chemistry calculations, embedding refers to a computational approach that combines different levels of theory to describe a system. It involves partitioning the system into a primary region of interest and an embedding environment. The primary region is treated at a higher level of theory, while the environment is described using a lower level of theory. Embedding methods allow for the accurate treatment of a small region of interest embedded in a larger system.
Canonical molecular orbitals: Canonical molecular orbitals, also known as spectroscopic orbitals or Hartree–Fock orbitals, are solutions to the electronic Schrödinger equation obtained within the Hartree–Fock approximation in quantum chemistry. They represent the molecular orbitals of a system and are obtained by diagonalizing the molecular orbital matrix. Canonical molecular orbitals provide a basis for describing the electronic structure of a molecule and are often used in electronic structure calculations to analyze bonding, molecular properties, and chemical reactivity.
Localized molecular orbitals (LMOs): LMOs are alternative representations of molecular orbitals that provide a localized description of electron density. LMOs are derived from canonical molecular orbitals through a transformation that maximizes the localization of electron density on specific atoms or groups within a molecule. LMOs are particularly useful for analyzing chemical bonding, molecular reactivity, and electron delocalization. They offer an intuitive and chemically interpretable representation of electron distribution in a molecule.
State-averaged: State-averaged refers to a computational approach in quantum chemistry and molecular electronic structure calculations. It involves optimizing wave functions for an average of electronic energies over multiple electronic states, such as different spin states or excited states. State-averaged methods are used to obtain accurate descriptions of molecular systems that exhibit strong degeneracy or to create a balanced treatment of multiple electronic states.
Orbital optimization: Orbital optimization is a procedure used in quantum chemical calculations to optimize the molecular orbitals that describe the electronic structure of a molecule. It involves iteratively adjusting the molecular orbitals to minimize the electronic energy of the system, typically using methods based on the variational principle. Orbital optimization helps improve the accuracy of electronic structure calculations and provides a more reliable description of molecular properties.
Variational quantum eigensovler (VQE): VQE is a quantum algorithm designed to solve for the ground-state energy of a quantum system using a variational approach. It combines classical optimization techniques with a quantum circuit ansatz to find the lowest energy eigenstate of a given Hamiltonian. VQE is a promising approach for near-term quantum computers to tackle problems in quantum chemistry and materials science.
Qubit: A qubit is the fundamental unit of quantum information in quantum computing. It is the quantum analogue of a classical bit, which represents a two-level quantum system with states usually referred to as |0⟩ and |1⟩. Qubits can exist in a superposition of states, which allows for parallel processing and the potential for exponential computational speedup in certain algorithms.
Quantum gates: Quantum gates are fundamental building blocks of quantum circuits and are analogous to classical logic gates in classical computing. They are represented by unitary operators that act on the quantum states of qubits. Quantum gates manipulate the quantum state of qubits to perform specific operations, such as rotations, entanglement generation, and information processing, in quantum algorithms.
Quantum circuit: A quantum circuit is a sequence of quantum gates applied to qubits to perform quantum computations. Similar to classical circuits composed of logic gates, quantum circuits manipulate the quantum state of qubits to implement quantum algorithms and computations. The gates in a quantum circuit typically perform unitary operations on qubits, such as rotations, entanglement operations, and measurements.
Unitary operations: In the context of quantum computing, unitary operations refer to transformations that preserve the norm of the quantum state and are reversible. A unitary operator is represented by a square matrix that is both Hermitian (equal to its own conjugate transpose) and unitary (its inverse is equal to its conjugate transpose). Unitary operations are fundamental in quantum mechanics and play a crucial role in quantum circuits where they enable the manipulation and evolution of quantum states while preserving their probabilistic interpretation.
Ansatz: In the context of quantum algorithms and quantum computing, an ansatz refers to a trial wave function or a specific form of a quantum circuit used to prepare a state of interest. The ansatz is chosen based on heuristics, intuition, or prior knowledge and is designed to capture the relevant features of the desired quantum state. The optimization of the ansatz parameters allows for the exploration of the solution space in variational quantum algorithms.
State preparation: State preparation, also known as initial state preparation, is the process of preparing a quantum system in a desired quantum state. In the context of quantum computing, it involves initializing the qubits or quantum registers in a specific configuration or superposition state required for a particular quantum algorithm or computation. State preparation is a crucial step in performing quantum computations and experiments.
Measurement: Measurement in quantum mechanics refers to the process of extracting information or obtaining outcomes from a quantum system. It involves interacting with the system in a way that projects it onto a particular eigenstate or superposition state. The measurement process in quantum computing is typically carried out by applying appropriate quantum gates followed by a measurement operation to provide classical data based on the quantum state of the qubits.
Noisy-intermediate scale quantum (NISQ): Near-term quantum computing, often referred to as noisy intermediate-scale quantum (NISQ) computing, represents the current state of quantum technology where devices possess a limited number of qubits and short coherence times. Research in NISQ focuses on harnessing these devices to explore quantum algorithms, error mitigation techniques, and applications in various fields, thereby paving the way for future advancements in quantum computing technologies.
Error mitigation: Error mitigation techniques play a crucial role in improving the reliability and accuracy of quantum computations, especially on noisy intermediate-scale quantum (NISQ) devices. By identifying and correcting errors that arise during quantum operations, these techniques help mitigate the impact of noise and imperfections inherent in current quantum hardware. Various approaches, such as randomized benchmarking, Pauli twirling, zero-noise extrapolation, machine learning-based methods, etc., are being explored to mitigate errors in NISQ quantum computations.
Error correction: Error correction is a vital aspect of quantum computing aimed at mitigating errors introduced during quantum operations. Quantum error correction codes, such as the surface code and the repetition code, are designed to detect and correct errors that occur because of noise and decoherence in quantum systems. These codes use redundancy and logical qubits to protect quantum information from errors, thereby enhancing the reliability of quantum computations. Error correction techniques play a crucial role in building fault-tolerant quantum computers capable of performing complex calculations with high accuracy and reliability.
Quantum advantage/Quantum supremacy: Quantum advantage, also known as quantum supremacy, refers to the state where a quantum computer can perform a specific computational task that is beyond the capabilities of the best classical computers. It signifies the ability of a quantum computer to solve certain problems more efficiently or to tackle computations that would take an impractically long time for classical computers. Achieving quantum advantage is a major goal in the field of quantum computing.
Time-reversal symmetric Hamiltonian: A time-reversal symmetric Hamiltonian, denoted as *H*, is a quantum mechanical operator representing the total energy of a system. This operator preserves its form under time reversal operations, which ensures that the system’s physical laws remain unchanged when the direction of time is reversed. Mathematically, the time reversal operator *T* satisfies the condition: *THT*^–1^ = *H*, which indicates that applying time reversal to the Hamiltonian restores its original form.

## References

[ref1] Schlögl R. Heterogeneous Catalysis. Angew. Chem., Int. Ed. 2015, 54, 3465–3520. 10.1002/anie.201410738. 25693734

[ref2] Van Santen R. A. Modern heterogeneous catalysis: an introduction; John Wiley & Sons, 2017.

[ref3] Friend C. M.; Xu B. Heterogeneous Catalysis: A Central Science for a Sustainable Future. Acc. Chem. Res. 2017, 50, 517–521. 10.1021/acs.accounts.6b00510. 28945397

[ref4] Bahrani S.; Mousavi S. M.; Hashemi S. A.; Ghaedi M. In Photocatalysis: Fundamental Processes and Applications; Ghaedi M., Ed.; Interface Science and Technology, Vol. 32; Elsevier, 2021; pp 443–498.

[ref5] Shimizu K.-i. Heterogeneous catalysis for the direct synthesis of chemicals by borrowing hydrogen methodology. Catal. Sci. Technol. 2015, 5, 1412–1427. 10.1039/C4CY01170H.

[ref6] Vogt C.; Weckhuysen B. M. The concept of active site in heterogeneous catalysis. Nature Reviews Chemistry 2022, 6, 89–111. 10.1038/s41570-021-00340-y. 37117296

[ref7] Schauermann S.; Nilius N.; Shaikhutdinov S.; Freund H.-J. Nanoparticles for Heterogeneous Catalysis: New Mechanistic Insights. Acc. Chem. Res. 2013, 46, 1673–1681. 10.1021/ar300225s. 23252628

[ref8] Huang W.; Li W.-X. Surface and interface design for heterogeneous catalysis. Phys. Chem. Chem. Phys. 2019, 21, 523–536. 10.1039/C8CP05717F. 30556573

[ref9] Kalz K. F.; Kraehnert R.; Dvoyashkin M.; Dittmeyer R.; Gläser R.; Krewer U.; Reuter K.; Grunwaldt J.-D. Future Challenges in Heterogeneous Catalysis: Understanding Catalysts under Dynamic Reaction Conditions. ChemCatChem. 2017, 9, 17–29. 10.1002/cctc.201600996. 28239429 PMC5299475

[ref10] Rana R.; Vila F. D.; Kulkarni A. R.; Bare S. R. Bridging the Gap between the X-ray Absorption Spectroscopy and the Computational Catalysis Communities in Heterogeneous Catalysis: A Perspective on the Current and Future Research Directions. ACS Catal. 2022, 12, 13813–13830. 10.1021/acscatal.2c03863.

[ref11] Weckhuysen B. M. Preface: recent advances in the in-situ characterization of heterogeneous catalysts. Chem. Soc. Rev. 2010, 39, 4557–4559. 10.1039/c0cs90031a.

[ref12] Chadwick H.; Beck R. D. Quantum state resolved gas-surface reaction dynamics experiments: a tutorial review. Chem. Soc. Rev. 2016, 45, 3576–3594. 10.1039/C5CS00476D. 26235656

[ref13] Park G. B.; Kruger B. C.; Borodin D.; Kitsopoulos T. N.; Wodtke A. M. Fundamental mechanisms for molecular energy conversion and chemical reactions at surfaces. Rep. Prog. Phys. 2019, 82, 096401 10.1088/1361-6633/ab320e. 31304916

[ref14] Auerbach D. J.; Tully J. C.; Wodtke A. M. Chemical dynamics from the gas-phase to surfaces. Natural Sciences 2021, 1, e10005 10.1002/ntls.10005.

[ref15] Rupprechter G. Operando Surface Spectroscopy and Microscopy during Catalytic Reactions: From Clusters via Nanoparticles to Meso-Scale Aggregates. Small 2021, 17, 2004289 10.1002/smll.202004289. 33694320 PMC11475487

[ref16] Groot I. M. N. Investigation of Active Catalysts at Work. Acc. Chem. Res. 2021, 54, 4334–4341. 10.1021/acs.accounts.1c00429. 34797651 PMC8655979

[ref17] Cheng H.-W.; Wang S.; Chen G.; Liu Z.; Caracciolo D.; Madiou M.; Shan S.; Zhang J.; He H.; Che R.; Zhong C.-J. Insights into Heterogeneous Catalysts under Reaction Conditions by In Situ/Operando Electron Microscopy. Adv. Energy Mater. 2022, 12, 2202097 10.1002/aenm.202202097.

[ref18] Banares M. A.; Daturi M. Understanding Catalysts by Time-/Space-Resolved Operando Methodologies. Catalysis Today 2023, 423, 114255 10.1016/j.cattod.2023.114255.

[ref19] Nørskov J. K.; Abild-Pedersen F.; Studt F.; Bligaard T. Density functional theory in surface chemistry and catalysis. Proc. Natl. Acad. Sci. U. S. A. 2011, 108, 937–943. 10.1073/pnas.1006652108. 21220337 PMC3024687

[ref20] Zhou L.; Zhuo L.; Yuan R.; Fu G. Theoretical modeling for interfacial catalysis. WIREs Computational Molecular Science 2021, 11, e1531 10.1002/wcms.1531.

[ref21] Chen B. W. J.; Xu L.; Mavrikakis M. Computational Methods in Heterogeneous Catalysis. Chem. Rev. 2021, 121, 1007–1048. 10.1021/acs.chemrev.0c01060. 33350813

[ref22] Stamatakis M. Kinetic modelling of heterogeneous catalytic systems. J. Phys.: Condens. Matter 2015, 27, 013001 10.1088/0953-8984/27/1/013001. 25393371

[ref23] Matera S.; Schneider W. F.; Heyden A.; Savara A. Progress in Accurate Chemical Kinetic Modeling, Simulations, and Parameter Estimation for Heterogeneous Catalysis. ACS Catal. 2019, 9, 6624–6647. 10.1021/acscatal.9b01234.

[ref24] Bruix A.; Margraf J. T.; Andersen M.; Reuter K. First-principles-based multiscale modelling of heterogeneous catalysis. Nature Catalysis 2019, 2, 659–670. 10.1038/s41929-019-0298-3.

[ref25] Jiang B.; Guo H. Dynamics in reactions on metal surfaces: A theoretical perspective. J. Chem. Phys. 2019, 150, 180901 10.1063/1.5096869. 31091904

[ref26] Dou W.; Subotnik J. E. Nonadiabatic Molecular Dynamics at Metal Surfaces. J. Phys. Chem. A 2020, 124, 757–771. 10.1021/acs.jpca.9b10698. 31916769

[ref27] Kroes G.-J. Computational approaches to dissociative chemisorption on metals: towards chemical accuracy. Phys. Chem. Chem. Phys. 2021, 23, 8962–9048. 10.1039/D1CP00044F. 33885053

[ref28] Grajciar L.; Heard C. J.; Bondarenko A. A.; Polynski M. V.; Meeprasert J.; Pidko E. A.; Nachtigall P. Towards operando computational modeling in heterogeneous catalysis. Chem. Soc. Rev. 2018, 47, 8307–8348. 10.1039/C8CS00398J. 30204184 PMC6240816

[ref29] Collinge G.; Yuk S. F.; Nguyen M.-T.; Lee M.-S.; Glezakou V.-A.; Rousseau R. Effect of Collective Dynamics and Anharmonicity on Entropy in Heterogenous Catalysis: Building the Case for Advanced Molecular Simulations. ACS Catal. 2020, 10, 9236–9260. 10.1021/acscatal.0c01501.

[ref30] Piccini G.; Lee M.-S.; Yuk S. F.; Zhang D.; Collinge G.; Kollias L.; Nguyen M.-T.; Glezakou V.-A.; Rousseau R. Ab initio molecular dynamics with enhanced sampling in heterogeneous catalysis. Catal. Sci. Technol. 2022, 12, 12–37. 10.1039/D1CY01329G.

[ref31] Schlexer-Lamoureux P.; Winther K. T.; Garrido-Torres J. A.; Streibel V.; Zhao M.; Bajdich M.; Abild-Pedersen F.; Bligaard T. Machine Learning for Computational Heterogeneous Catalysis. ChemCatChem. 2019, 11, 3581–3601. 10.1002/cctc.201900595.

[ref32] Jones R. O. Density functional theory: Its origins, rise to prominence, and future. Rev. Mod. Phys. 2015, 87, 897–923. 10.1103/RevModPhys.87.897.

[ref33] Mardirossian N.; Head-Gordon M. Thirty years of density functional theory in computational chemistry: an overview and extensive assessment of 200 density functionals. Mol. Phys. 2017, 115, 2315–2372. 10.1080/00268976.2017.1333644.

[ref34] Sholl D. S.; Steckel J. A. Density functional theory: a practical introduction; John Wiley & Sons, 2022.

[ref35] Gaggioli C. A.; Stoneburner S. J.; Cramer C. J.; Gagliardi L. Beyond Density Functional Theory: The Multiconfigurational Approach To Model Heterogeneous Catalysis. ACS Catal. 2019, 9, 8481–8502. 10.1021/acscatal.9b01775.

[ref36] Janesko B. G. Strong correlation in surface chemistry. Mol. Simul. 2017, 43, 394–405. 10.1080/08927022.2016.1261136.

[ref37] Chen J.; Jin Z.; Dou W.; Subotnik J. Electronic Structure for Multielectronic Molecules near a Metal Surface. J. Phys. Chem. C 2021, 125, 2884–2899. 10.1021/acs.jpcc.0c08750.

[ref38] Chen J.; Dou W.; Subotnik J. Active Spaces and Non-Orthogonal Configuration Interaction Approaches for Investigating Molecules on Metal Surfaces. J. Chem. Theory Comput. 2022, 18, 7321–7335. 10.1021/acs.jctc.2c00740. 36371807

[ref39] Roos B. A new method for large-scale Cl calculations. Chem. Phys. Lett. 1972, 15, 153–159. 10.1016/0009-2614(72)80140-4.

[ref40] Olsen J.; Roos B. O.; Jorgensen P.; Jensen H. J. A. Determinant based configuration interaction algorithms for complete and restricted configuration interaction spaces. J. Chem. Phys. 1988, 89, 2185–2192. 10.1063/1.455063.

[ref41] Roos B. O.; Linse P.; Siegbahn P. E.; Blomberg M. R. A simple method for the evaluation of the second-order-perturbation energy from external double-excitations with a CASSCF reference wavefunction. Chem. Phys. 1982, 66, 197–207. 10.1016/0301-0104(82)88019-1.

[ref42] Andersson K.; Malmqvist P. A.; Roos B. O.; Sadlej A. J.; Wolinski K. Second-order perturbation theory with a CASSCF reference function. J. Phys. Chem. 1990, 94, 5483–5488. 10.1021/j100377a012.

[ref43] Andersson K.; Malmqvist P.; Roos B. O. Second-order perturbation theory with a complete active space self-consistent field reference function. J. Chem. Phys. 1992, 96, 1218–1226. 10.1063/1.462209.

[ref44] Angeli C.; Cimiraglia R.; Evangelisti S.; Leininger T.; Malrieu J.-P. Introduction of n-electron valence states for multireference perturbation theory. J. Chem. Phys. 2001, 114, 10252–10264. 10.1063/1.1361246.

[ref45] Angeli C.; Cimiraglia R.; Malrieu J.-P. N-electron valence state perturbation theory: a fast implementation of the strongly contracted variant. Chem. Phys. Lett. 2001, 350, 297–305. 10.1016/S0009-2614(01)01303-3.

[ref46] Angeli C.; Cimiraglia R.; Malrieu J.-P. n-electron valence state perturbation theory: A spinless formulation and an efficient implementation of the strongly contracted and of the partially contracted variants. J. Chem. Phys. 2002, 117, 9138–9153. 10.1063/1.1515317.

[ref47] Olsen J. The CASSCF method: A perspective and commentary. Int. J. Quantum Chem. 2011, 111, 3267–3272. 10.1002/qua.23107.

[ref48] Vogiatzis K. D.; Ma D.; Olsen J.; Gagliardi L.; de Jong W. A. Pushing configuration-interaction to the limit: Towards massively parallel MCSCF calculations. J. Chem. Phys. 2017, 147, 184111 10.1063/1.4989858. 29141437

[ref49] Smith J. E. T.; Mussard B.; Holmes A. A.; Sharma S. Cheap Near Exact CASSCF with Large Active Spaces. J. Chem. Theory Comput. 2017, 13, 5468–5478. 10.1021/acs.jctc.7b00900. 28968097

[ref50] Levine D. S.; Hait D.; Tubman N. M.; Lehtola S.; Whaley K. B.; Head-Gordon M. CASSCF with Extremely Large Active Spaces Using the Adaptive Sampling Configuration Interaction Method. J. Chem. Theory Comput. 2020, 16, 2340–2354. 10.1021/acs.jctc.9b01255. 32109055

[ref51] Chan G. K.-L.; Sharma S. The Density Matrix Renormalization Group in Quantum Chemistry. Annu. Rev. Phys. Chem. 2011, 62, 465–481. 10.1146/annurev-physchem-032210-103338. 21219144

[ref52] Frahm L.-H.; Pfannkuche D. Ultrafast ab Initio Quantum Chemistry Using Matrix Product States. J. Chem. Theory Comput. 2019, 15, 2154–2165. 10.1021/acs.jctc.8b01291. 30817156

[ref53] Nakatani N.; Chan G. K.-L. Efficient tree tensor network states (TTNS) for quantum chemistry: Generalizations of the density matrix renormalization group algorithm. J. Chem. Phys. 2013, 138, 134113 10.1063/1.4798639. 23574214

[ref54] Kassal I.; Whitfield J. D.; Perdomo-Ortiz A.; Yung M.-H.; Aspuru-Guzik A. Simulating Chemistry Using Quantum Computers. Annu. Rev. Phys. Chem. 2011, 62, 185–207. 10.1146/annurev-physchem-032210-103512. 21166541

[ref55] Cao Y.; Romero J.; Olson J. P.; Degroote M.; Johnson P. D.; Kieferová M.; Kivlichan I. D.; Menke T.; Peropadre B.; Sawaya N. P. D.; Sim S.; Veis L.; Aspuru-Guzik A. Quantum Chemistry in the Age of Quantum Computing. Chem. Rev. 2019, 119, 10856–10915. 10.1021/acs.chemrev.8b00803. 31469277

[ref56] McArdle S.; Endo S.; Aspuru-Guzik A.; Benjamin S. C.; Yuan X. Quantum computational chemistry. Rev. Mod. Phys. 2020, 92, 015003 10.1103/RevModPhys.92.015003.

[ref57] Bauer B.; Bravyi S.; Motta M.; Chan G. K.-L. Quantum Algorithms for Quantum Chemistry and Quantum Materials Science. Chem. Rev. 2020, 120, 12685–12717. 10.1021/acs.chemrev.9b00829. 33090772

[ref58] Motta M.; Rice J. E. Emerging quantum computing algorithms for quantum chemistry. WIREs Computational Molecular Science 2022, 12, e1580 10.1002/wcms.1580.

[ref59] Liu H.; Low G. H.; Steiger D. S.; Häner T.; Reiher M.; Troyer M. Prospects of quantum computing for molecular sciences. Materials Theory 2022, 6, 11 10.1186/s41313-021-00039-z.

[ref60] Peruzzo A.; McClean J.; Shadbolt P.; Yung M.-H.; Zhou X.-Q.; Love P. J.; Aspuru-Guzik A.; O'Brien J. L. A variational eigenvalue solver on a photonic quantum processor. Nat. Commun. 2014, 5, 4213 10.1038/ncomms5213. 25055053 PMC4124861

[ref61] Larocca M.; Thanasilp S.; Wang S.; Sharma K.; Biamonte J.; Coles P. J.; Cincio L.; McClean J. R.; Holmes Z.; Cerezo M. A Review of Barren Plateaus in Variational Quantum Computing. arXiv, May 1, 2024, 2405.00781, ver. 1. 10.48550/arXiv.2405.00781.

[ref62] Lee S.; Lee J.; Zhai H.; Tong Y.; Dalzell A. M.; Kumar A.; Helms P.; Gray J.; Cui Z.-H.; Liu W.; et al. Evaluating the evidence for exponential quantum advantage in ground-state quantum chemistry. Nat. Commun. 2023, 14, 1952 10.1038/s41467-023-37587-6. 37029105 PMC10082187

[ref63] Berry D. W.; Tong Y.; Khattar T.; White A.; Kim T. I.; Boixo S.; Lin L.; Lee S.; Chan G. K.-L.; Babbush R.; Rubin N. C. Rapid initial state preparation for the quantum simulation of strongly correlated molecules. arXiv, September 18, 2024, 2409.11748, ver. 1. 10.48550/arXiv.2409.11748.

[ref64] Jones L. O.; Mosquera M. A.; Schatz G. C.; Ratner M. A. Embedding Methods for Quantum Chemistry: Applications from Materials to Life Sciences. J. Am. Chem. Soc. 2020, 142, 3281–3295. 10.1021/jacs.9b10780. 31986877

[ref65] Ma H.; Govoni M.; Galli G. Quantum simulations of materials on near-term quantum computers. npj Computational Materials 2020, 6, 85 10.1038/s41524-020-00353-z.

[ref66] Muechler L.; Badrtdinov D. I.; Hampel A.; Cano J.; Rosner M.; Dreyer C. E. Quantum embedding methods for correlated excited states of point defects: Case studies and challenges. Phys. Rev. B 2022, 105, 235104 10.1103/PhysRevB.105.235104.

[ref67] Bravyi S.; Dial O.; Gambetta J. M.; Gil D.; Nazario Z. The future of quantum computing with superconducting qubits. J. Appl. Phys. 2022, 132, 160902 10.1063/5.0082975.

[ref68] Alexeev Y.; Amsler M.; Barroca M. A.; Bassini S.; Battelle T.; Camps D.; Casanova D.; Choi Y. J.; Chong F. T.; Chung C.; et al. Quantum-centric Supercomputing for Materials Science: A Perspective on Challenges and Future Directions. Future Generation Computer Systems 2024, 160, 666–710. 10.1016/j.future.2024.04.060.

[ref69] Medford A. J.; Wellendorff J.; Vojvodic A.; Studt F.; Abild-Pedersen F.; Jacobsen K. W.; Bligaard T.; Nørskov J. K. Assessing the reliability of calculated catalytic ammonia synthesis rates. Science 2014, 345, 197–200. 10.1126/science.1253486. 25013071

[ref70] Sutton J. E.; Guo W.; Katsoulakis M. A.; Vlachos D. G. Effects of correlated parameters and uncertainty in electronic-structure-based chemical kinetic modelling. Nature Chem. 2016, 8, 331 10.1038/nchem.2454. 27001728

[ref71] Walker E.; Ammal S. C.; Terejanu G. A.; Heyden A. Uncertainty quantification framework applied to the water-gas shift reaction over Pt-based catalysts. J. Phys. Chem. C 2016, 120, 10328–10339. 10.1021/acs.jpcc.6b01348.

[ref72] Janet J. P.; Duan C.; Yang T.; Nandy A.; Kulik H. J. A quantitative uncertainty metric controls error in neural network-driven chemical discovery. Chemical science 2019, 10, 7913–7922. 10.1039/C9SC02298H. 31588334 PMC6764470

[ref73] Walker E. A.; Pallathadka S. A. How a Quantum Computer Could Solve a Microkinetic Model. J. Phys. Chem. Lett. 2021, 12, 592–597. 10.1021/acs.jpclett.0c03363. 33382628

[ref74] Becerra A.; Prabhu A.; Rongali M. S.; Velpur S. C. S.; Debusschere B.; Walker E. A. How a Quantum Computer Could Quantify Uncertainty in Microkinetic Models. J. Phys. Chem. Lett. 2021, 12, 6955–6960. 10.1021/acs.jpclett.1c01917. 34283593

[ref75] Becerra A.; Diaz-Ibarra O. H.; Kim K.; Debusschere B.; Walker E. A. How a quantum computer could accurately solve a hydrogen-air combustion model. Digital Discovery 2022, 1, 511–518. 10.1039/D2DD00049K.

[ref76] Harrow A. W.; Hassidim A.; Lloyd S. Quantum Algorithm for Linear Systems of Equations. Phys. Rev. Lett. 2009, 103, 150502 10.1103/PhysRevLett.103.150502. 19905613

[ref77] Bravo-Prieto C.; LaRose R.; Cerezo M.; Subasi Y.; Cincio L.; Coles P. J. Variational Quantum Linear Solver. Quantum 2023, 7, 1188 10.22331/q-2023-11-22-1188.

[ref78] Paudel H. P.; Syamlal M.; Crawford S. E.; Lee Y.-L.; Shugayev R. A.; Lu P.; Ohodnicki P. R.; Mollot D.; Duan Y. Quantum Computing and Simulations for Energy Applications: Review and Perspective. ACS Engineering Au 2022, 2, 151–196. 10.1021/acsengineeringau.1c00033.

[ref79] Cheng H.-P.; Deumens E.; Freericks J. K.; Li C.; Sanders B. A. Application of Quantum Computing to Biochemical Systems: A Look to the Future. Frontiers in Chemistry 2020, 8. 10.3389/fchem.2020.587143 PMC7732423 33330375

[ref80] Baiardi A.; Christandl M.; Reiher M. Quantum Computing for Molecular Biology**. ChemBioChem. 2023, 24, e202300120 10.1002/cbic.202300120. 37151197

[ref81] Blunt N. S.; Camps J.; Crawford O.; Izsak R.; Leontica S.; Mirani A.; Moylett A. E.; Scivier S. A.; Sanderhauf C.; Schopf P.; Taylor J. M.; Holzmann N. Perspective on the Current State-of-the-Art of Quantum Computing for Drug Discovery Applications. J. Chem. Theory Comput. 2022, 18, 7001–7023. 10.1021/acs.jctc.2c00574. 36355616 PMC9753588

[ref82] Joseph I.; Shi Y.; Porter M. D.; Castelli A. R.; Geyko V. I.; Graziani F. R.; Libby S. B.; DuBois J. L. Quantum computing for fusion energy science applications. Physics of Plasmas 2023, 30, 010501 10.1063/5.0123765.

[ref83] Reiher M.; Wiebe N.; Svore K. M.; Wecker D.; Troyer M. Elucidating reaction mechanisms on quantum computers. Proc. Natl. Acad. Sci. U. S. A. 2017, 114, 7555–7560. 10.1073/pnas.1619152114. 28674011 PMC5530650

[ref84] Tiwari A. K.; Nave S.; Jackson B. Methane Dissociation on Ni(111): A New Understanding of the Lattice Effect. Phys. Rev. Lett. 2009, 103, 253201 10.1103/PhysRevLett.103.253201. 20366254

[ref85] Jackson B.; Nattino F.; Kroes G.-J. Dissociative chemisorption of methane on metal surfaces: Tests of dynamical assumptions using quantum models and ab initio molecular dynamics. J. Chem. Phys. 2014, 141, 054102 10.1063/1.4891327. 25106565

[ref86] Rogal J.; Reuter K. Ab Initio Atomistic Thermodynamics for Surfaces: A Primer. In Experiment Modeling and Simulation of GasSurface Interactions for Reactive Flows in Hypersonic Flights; NATO Research and Technology Organisation: Neuilly-sur-Seine, France, 2007; pp 2-1–2-18

[ref87] Reuter K. Ab Initio Thermodynamics and First-Principles Microkinetics for Surface Catalysis. Catal. Lett. 2016, 146, 541–563. 10.1007/s10562-015-1684-3.

[ref88] Chizallet C. Achievements and Expectations in the Field of Computational Heterogeneous Catalysis in an Innovation Context. Top. Catal. 2022, 65, 69–81. 10.1007/s11244-021-01489-y.

[ref89] Sarwar M.; Cooper C.; Briquet L.; Ukpong A.; Perry C.; Jones G. Atomic-scale modelling and its application to catalytic materials science. Johnson Matthey Technology Review 2015, 59, 257–283. 10.1595/205651315X687975.

[ref90] Jones G. Industrial computational catalysis and its relation to the digital revolution. Nature Catalysis 2018, 1, 311–313. 10.1038/s41929-018-0074-9.

[ref91] Ras E.-J.; Rothenberg G. Heterogeneous catalyst discovery using 21st century tools: a tutorial. RSC Adv. 2014, 4, 5963–5974. 10.1039/c3ra45852k.

[ref92] Holewinski A.; Xin H.; Nikolla E.; Linic S. Identifying optimal active sites for heterogeneous catalysis by metal alloys based on molecular descriptors and electronic structure engineering. Current Opinion in Chemical Engineering 2013, 2, 312–319. 10.1016/j.coche.2013.04.006.

[ref93] Pirro L.; Mendes P. S. F.; Paret S.; Vandegehuchte B. D.; Marin G. B.; Thybaut J. W. Descriptor-property relationships in heterogeneous catalysis: exploiting synergies between statistics and fundamental kinetic modelling. Catal. Sci. Technol. 2019, 9, 3109–3125. 10.1039/C9CY00719A.

[ref94] Zhao Z.-J.; Liu S.; Zha S.; Cheng D.; Studt F.; Henkelman G.; Gong J. Theory-guided design of catalytic materials using scaling relationships and reactivity descriptors. Nature Reviews Materials 2019, 4, 792–804. 10.1038/s41578-019-0152-x.

[ref95] Lazaridou A.; Smith L. R.; Pattisson S.; Dummer N. F.; Smit J. J.; Johnston P.; Hutchings G. J. Recognizing the best catalyst for a reaction. Nature Reviews Chemistry 2023, 7, 287–295. 10.1038/s41570-023-00470-5. 37117418

[ref96] Araujo R. B.; Rodrigues G. L. S.; dos Santos E. C.; Pettersson L. G. M. Adsorption energies on transition metal surfaces: towards an accurate and balanced description. Nat. Commun. 2022, 13, 6853 10.1038/s41467-022-34507-y. 36369277 PMC9652424

[ref97] Eisenstein O.; Shaik S. Computational Catalysis: A Land of Opportunities. Top. Catal. 2022, 65, 1–5. 10.1007/s11244-021-01555-5.35153451

[ref98] Kim J.; Choi S.; Cho J.; Kim S. Y.; Jang H. W. Toward Multicomponent Single-Atom Catalysis for Efficient Electrochemical Energy Conversion. ACS Materials Au 2022, 2, 1–20. 10.1021/acsmaterialsau.1c00041. 36855696 PMC9888646

[ref99] Li Z.; Ma R.; Ju Q.; Liu Q.; Liu L.; Zhu Y.; Yang M.; Wang J. Spin engineering of single-site metal catalysts. Innovation 2022, 3, 100268 10.1016/j.xinn.2022.100268. 35789959 PMC9249949

[ref100] Li X.; Gong H.; Zhuang Q.; Wang B.; Zheng X.; Yang J. Reaction on a Rink: Kondo-Enhanced Heterogeneous Single-Atom Catalysis. J. Phys. Chem. C 2021, 125, 21488–21495. 10.1021/acs.jpcc.1c07731.

[ref101] Biz C.; Gracia J.; Fianchini M. Review on Magnetism in Catalysis: From Theory to PEMFC Applications of 3d Metal Pt-Based Alloys. International Journal of Molecular Sciences 2022, 23, 14768 10.3390/ijms232314768. 36499096 PMC9739051

[ref102] Biz C.; Fianchini M.; Gracia J. Strongly Correlated Electrons in Catalysis: Focus on Quantum Exchange. ACS Catal. 2021, 11, 14249–14261. 10.1021/acscatal.1c03135.

[ref103] Biz C.; Fianchini M.; Polo V.; Gracia J. Magnetism and Heterogeneous Catalysis: In Depth on the Quantum Spin-Exchange Interactions in Pt3M (M = V, Cr, Mn, Fe, Co, Ni, and Y)(111) Alloys. ACS Appl. Mater. Interfaces 2020, 12, 50484–50494. 10.1021/acsami.0c15353. 33124822

[ref104] Biz C.; Fianchini M.; Gracia J. Catalysis Meets Spintronics; Spin Potentials Associated with Open-Shell Orbital Configurations Enhance the Activity of Pt3Co Nanostructures for Oxygen Reduction: A Density Functional Theory Study. ACS Applied Nano Materials 2020, 3, 506–515. 10.1021/acsanm.9b02067.

[ref105] Cao A.; Nørskov J. K. Spin Effects in Chemisorption and Catalysis. ACS Catal. 2023, 13, 3456–3462. 10.1021/acscatal.2c06319.

[ref106] Xie W.; Xu J.; Chen J.; Wang H.; Hu P. Achieving theory–experiment parity for activity and selectivity in heterogeneous catalysis using microkinetic modeling. Acc. Chem. Res. 2022, 55, 1237–1248. 10.1021/acs.accounts.2c00058. 35442027 PMC9069691

[ref107] Motagamwala A. H.; Ball M. R.; Dumesic J. A. Microkinetic analysis and scaling relations for catalyst design. Annu. Rev. Chem. Biomol. Eng. 2018, 9, 413–450. 10.1146/annurev-chembioeng-060817-084103. 29641915

[ref108] Matera S.; Schneider W. F.; Heyden A.; Savara A. Progress in accurate chemical kinetic modeling, simulations, and parameter estimation for heterogeneous catalysis. ACS Catal. 2019, 9, 6624–6647. 10.1021/acscatal.9b01234.

[ref109] Grimme S. Semiempirical GGA-type density functional constructed with a long-range dispersion correction. J. Comput. Chem. 2006, 27, 1787–1799. 10.1002/jcc.20495. 16955487

[ref110] Grimme S.; Antony J.; Ehrlich S.; Krieg H. A consistent and accurate ab initio parametrization of density functional dispersion correction (DFT-D) for the 94 elements H-Pu. J. Chem. Phys. 2010, 132, 154104 10.1063/1.3382344. 20423165

[ref111] Caldeweyher E.; Mewes J.-M.; Ehlert S.; Grimme S. Extension and evaluation of the D4 London-dispersion model for periodic systems. Phys. Chem. Chem. Phys. 2020, 22, 8499–8512. 10.1039/D0CP00502A. 32292979

[ref112] Tkatchenko A.; Scheffler M. Accurate Molecular Van Der Waals Interactions from Ground-State Electron Density and Free-Atom Reference Data. Phys. Rev. Lett. 2009, 102, 073005 10.1103/PhysRevLett.102.073005. 19257665

[ref113] Gould T.; Lebégue S.; Ángyán J. G.; Bučko T. A Fractionally Ionic Approach to Polarizability and van der Waals Many-Body Dispersion Calculations. J. Chem. Theory Comput. 2016, 12, 5920–5930. 10.1021/acs.jctc.6b00925. 27951673

[ref114] Olsen T.; Thygesen K. S. Random phase approximation applied to solids, molecules, and graphene-metal interfaces: From van der Waals to covalent bonding. Phys. Rev. B 2013, 87, 075111 10.1103/PhysRevB.87.075111.

[ref115] Gerrits N.; Smeets E. W. F.; Vuckovic S.; Powell A. D.; Doblhoff-Dier K.; Kroes G.-J. Density Functional Theory for Molecule–Metal Surface Reactions: When Does the Generalized Gradient Approximation Get It Right, and What to Do If It Does Not. J. Phys. Chem. Lett. 2020, 11, 10552–10560. 10.1021/acs.jpclett.0c02452. 33295770 PMC7751010

[ref116] Wodtke A. M.; Matsiev D.; Auerbach D. J. Energy transfer and chemical dynamics at solid surfaces: The special role of charge transfer. Prog. Surf. Sci. 2008, 83, 167–214. 10.1016/j.progsurf.2008.02.001.

[ref117] Golibrzuch K.; Bartels N.; Auerbach D. J.; Wodtke A. M. The Dynamics of Molecular Interactions and Chemical Reactions at Metal Surfaces: Testing the Foundations of Theory. Annu. Rev. Phys. Chem. 2015, 66, 399–425. 10.1146/annurev-physchem-040214-121958. 25580627

[ref118] Libisch F.; Huang C.; Liao P.; Pavone M.; Carter E. A. Origin of the energy barrier to chemical reactions of O 2 on Al (111): Evidence for charge transfer, not spin selection. Physical review letters 2012, 109, 198303 10.1103/PhysRevLett.109.198303. 23215432

[ref119] Przybylski K.; Koutecky J.; Bonačić-Koutecký V.; von Ragué-Schleyer P.; Guest M. F. An ab-initio configuration interaction study of the reaction between small lithium clusters (Li4, Li6) and H2 molecule. J. Chem. Phys. 1991, 94, 5533–5543. 10.1063/1.460489.

[ref120] Healy S. B.; Filippi C.; Kratzer P.; Penev E.; Scheffler M. Role of Electronic Correlation in the Si(100) Reconstruction: A Quantum Monte Carlo Study. Phys. Rev. Lett. 2001, 87, 016105 10.1103/PhysRevLett.87.016105. 11461481

[ref121] Choi J.-H.; Kim K. S.; Cho J.-H. Antiferromagnetic spin ordering in the dissociative adsorption of H2 on Si(001): Density-functional calculations. J. Chem. Phys. 2009, 131, 244704 10.1063/1.3276916. 20059096

[ref122] Montemore M. M.; van Spronsen M. A.; Madix R. J.; Friend C. M. O2 Activation by Metal Surfaces: Implications for Bonding and Reactivity on Heterogeneous Catalysts. Chem. Rev. 2018, 118, 2816–2862. 10.1021/acs.chemrev.7b00217. 29116787

[ref123] Behler J.; Delley B.; Lorenz S.; Reuter K.; Scheffler M. Dissociation of O_2_ at Al(111): The Role of Spin Selection Rules. Phys. Rev. Lett. 2005, 94, 036104 10.1103/PhysRevLett.94.036104. 15698287

[ref124] Behler J.; Delley B.; Reuter K.; Scheffler M. Nonadiabatic potential-energy surfaces by constrained density-functional theory. Phys. Rev. B 2007, 75, 115409 10.1103/PhysRevB.75.115409.

[ref125] Carbogno C.; Behler J.; Groß A.; Reuter K. Fingerprints for Spin-Selection Rules in the Interaction Dynamics of O_2_ at Al(111). Phys. Rev. Lett. 2008, 101, 096104 10.1103/PhysRevLett.101.096104. 18851627

[ref126] Carbogno C.; Behler J.; Reuter K.; Groß A. Signatures of nonadiabatic O_2_ dissociation at Al(111): First-principles fewest-switches study. Phys. Rev. B 2010, 81, 035410 10.1103/PhysRevB.81.035410.

[ref127] Katz G.; Kosloff R.; Zeiri Y. Abstractive dissociation of oxygen over Al(111): A nonadiabatic quantum model. J. Chem. Phys. 2004, 120, 3931–3948. 10.1063/1.1635360. 15268559

[ref128] Behler J.; Reuter K.; Scheffler M. Nonadiabatic effects in the dissociation of oxygen molecules at the Al(111) surface. Phys. Rev. B 2008, 77, 115421 10.1103/PhysRevB.77.115421.

[ref129] Libisch F.; Huang C.; Carter E. A. Embedded Correlated Wavefunction Schemes: Theory and Applications. Acc. Chem. Res. 2014, 47, 2768–2775. 10.1021/ar500086h. 24873211

[ref130] Swart M.; Costas M. Spin states in biochemistry and inorganic chemistry; John Wiley & Sons: Nashville, TN, 2015.

[ref131] Swart M. In New Directions in the Modeling of Organometallic Reactions; Lledós A., Ujaque G., Eds.; Springer International Publishing: Cham, 2020; pp 191–226.

[ref132] Goings J. J.; White A.; Lee J.; Tautermann C. S.; Degroote M.; Gidney C.; Shiozaki T.; Babbush R.; Rubin N. C. Reliably assessing the electronic structure of cytochrome P450 on today’s classical computers and tomorrow’s quantum computers. Proc. Natl. Acad. Sci. U. S. A. 2022, 119, e2203533119 10.1073/pnas.2203533119. 36095200 PMC9499570

[ref133] Tsai R.; Yu C. A.; Gunsalus I. C.; Peisach J.; Blumberg W.; Orme-Johnson W. H.; Beinert H. Spin-State Changes in Cytochrome P-450cam on Binding of Specific Substrates. Proc. Natl. Acad. Sci. U. S. A. 1970, 66, 1157–1163. 10.1073/pnas.66.4.1157. 4319883 PMC335800

[ref134] McQuarters A. B.; Wolf M. W.; Hunt A. P.; Lehnert N. 1958–2014: After 56 years of research, cytochrome P450 reactivity is finally explained. Angew. Chem. Int. Ed. 2014, 53 (19), 4750–4752. 10.1002/anie.201402404. 24711286

[ref135] Lu X.; Wang S.; Qin J.-H. Isolating Fe-O2 intermediates in dioxygen activation by iron porphyrin complexes. Molecules 2022, 27, 4690 10.3390/molecules27154690. 35897870 PMC9332324

[ref136] Cao L.; Ryde U. Influence of the protein and DFT method on the broken-symmetry and spin states in nitrogenase. Int. J. Quantum Chem. 2018, 118, e25627 10.1002/qua.25627.

[ref137] Li Z.; Li J.; Dattani N. S.; Umrigar C. J.; Chan G. K.-L. The electronic complexity of the ground-state of the FeMo cofactor of nitrogenase as relevant to quantum simulations. J. Chem. Phys. 2019, 150, 024302 10.1063/1.5063376. 30646701

[ref138] Spatzal T.; Einsle O.; Andrade S. L. Analysis of the Magnetic Properties of Nitrogenase FeMo Cofactor by Single-Crystal EPR Spectroscopy. Angew. Chem., Int. Ed. 2013, 52, 10116–10119. 10.1002/anie.201303000. 23929797

[ref139] Siegbahn P. E. The mechanism for nitrogenase including all steps. Phys. Chem. Chem. Phys. 2019, 21, 15747–15759. 10.1039/C9CP02073J. 31276128

[ref140] Buchachenko A. L.; Berdinsky V. L. Spin catalysis as a new type of catalysis in chemistry. Russ. Chem. Rev. 2004, 73, 1033 10.1070/RC2004v073n11ABEH000888.

[ref141] Khavryuchenko O. V.; Khavryuchenko V. D.; Su D. Spin catalysts: A quantum trigger for chemical reactions. Chinese Journal of Catalysis 2015, 36, 1656–1661. 10.1016/S1872-2067(15)60948-2.

[ref142] Hammer B.; Nørskov J. Electronic factors determining the reactivity of metal surfaces. Surf. Sci. 1995, 343, 211–220. 10.1016/0039-6028(96)80007-0.

[ref143] Bhattacharjee S.; Waghmare U. V.; Lee S.-C. An improved d-band model of the catalytic activity of magnetic transition metal surfaces. Sci. Rep. 2016, 6, 35916 10.1038/srep35916. 27808100 PMC5093898

[ref144] Hedstrom S.; dos Santos E. C.; Liu C.; Chan K.; Abild-Pedersen F.; Pettersson L. G. M. Spin Uncoupling in Chemisorbed OCCO and CO2 Two High-Energy Intermediates in Catalytic CO2 Reduction. J. Phys. Chem. C 2018, 122, 12251–12258. 10.1021/acs.jpcc.8b02165.

[ref145] Cao A.; Bukas V. J.; Shadravan V.; Wang Z.; Li H.; Kibsgaard J.; Chorkendorff I.; Nørskov J. K. A spin promotion effect in catalytic ammonia synthesis. Nat. Commun. 2022, 13, 2382 10.1038/s41467-022-30034-y. 35501341 PMC9061734

[ref146] Zhao Z.; Wang Y.; Yang X.; Quan J.; Kruger B. C.; Stoicescu P.; Nieman R.; Auerbach D. J.; Wodtke A. M.; Guo H.; Park G. B. Spin-dependent reactivity and spin-flipping dynamics in oxygen atom scattering from graphite. Nat. Chem. 2023, 15, 1006 10.1038/s41557-023-01204-2. 37217785 PMC10322699

[ref147] Zhao Y.; Li B.; Yin R.; Wang Y.; Yang J.; Zhang Z.; Wang B. Spin Selection Rule in Single-Site Catalysis of Molecular Oxygen Adsorption on Transition-Metal Phthalocyanines. J. Phys. Chem. C 2019, 123, 28158–28167. 10.1021/acs.jpcc.9b07720.

[ref148] Liang Y.; Lihter M.; Lingenfelder M. Spin-Control in Electrocatalysis for Clean Energy. Isr. J. Chem. 2022, 62, e202200052 10.1002/ijch.202200052.

[ref149] Lin C.-C.; et al. Spin-Polarized Photocatalytic CO2 Reduction of Mn-Doped Perovskite Nanoplates. J. Am. Chem. Soc. 2022, 144, 15718–15726. 10.1021/jacs.2c06060. 35975916

[ref150] Jacob C. R.; Reiher M. Spin in density-functional theory. Int. J. Quantum Chem. 2012, 112, 3661–3684. 10.1002/qua.24309.

[ref151] Yang X.-F.; Wang A.; Qiao B.; Li J.; Liu J.; Zhang T. Single-Atom Catalysts: A New Frontier in Heterogeneous Catalysis. Acc. Chem. Res. 2013, 46, 1740–1748. 10.1021/ar300361m. 23815772

[ref152] Beniya A.; Higashi S. Towards dense single-atom catalysts for future automotive applications. Nature Catalysis 2019, 2, 590–602. 10.1038/s41929-019-0282-y.

[ref153] Zhang H.; Lu X. F.; Wu Z.-P.; Lou X. W. D. Emerging multifunctional single-atom catalysts/nanozymes. ACS Central Science 2020, 6, 1288–1301. 10.1021/acscentsci.0c00512. 32875072 PMC7453415

[ref154] Singh B.; Sharma V.; Gaikwad R. P.; Fornasiero P.; Zbořil R.; Gawande M. B. Single-atom catalysts: a sustainable pathway for the advanced catalytic applications. Small 2021, 17, 2006473 10.1002/smll.202006473. 33624397

[ref155] Shan J.; Ye C.; Jiang Y.; Jaroniec M.; Zheng Y.; Qiao S.-Z. Metal-metal interactions in correlated single-atom catalysts. Science Advances 2022, 8, eabo0762 10.1126/sciadv.abo0762. 35486734 PMC9054016

[ref156] Li L.; Chang X.; Lin X.; Zhao Z.-J.; Gong J. Theoretical insights into single-atom catalysts. Chem. Soc. Rev. 2020, 49, 8156–8178. 10.1039/D0CS00795A. 32870221

[ref157] Zhang W.; Fu Q.; Luo Q.; Sheng L.; Yang J. Understanding single-atom catalysis in view of theory. JACS Au 2021, 1, 2130–2145. 10.1021/jacsau.1c00384. 34977885 PMC8715482

[ref158] Di Liberto G.; Pacchioni G. Modeling Single-Atom Catalysis. Adv. Mater. 2023, 35, 2307150 10.1002/adma.202307150. 37749881

[ref159] Liu F.; Yang T.; Yang J.; Xu E.; Bajaj A.; Kulik H. J. Bridging the Homogeneous-Heterogeneous Divide: Modeling Spin for Reactivity in Single Atom Catalysis. Frontiers in Chemistry 2019, 7, 00219 10.3389/fchem.2019.00219. PMC6476907 31041303

[ref160] Giulimondi V.; Mitchell S.; Pérez-Ramírez J. Challenges and Opportunities in Engineering the Electronic Structure of Single-Atom Catalysts. ACS Catal. 2023, 13, 2981–2997. 10.1021/acscatal.2c05992. 36910873 PMC9990067

[ref161] Zhang L.; Ren X.; Zhao X.; Zhu Y.; Pang R.; Cui P.; Jia Y.; Li S.; Zhang Z. Synergetic charge transfer and spin selection in CO oxidation at neighboring magnetic single-atom catalyst sites. Nano Lett. 2022, 22, 3744–3750. 10.1021/acs.nanolett.2c00711. 35437988

[ref162] Zhong W.; Qiu Y.; Shen H.; Wang X.; Yuan J.; Jia C.; Bi S.; Jiang J. Electronic spin moment as a catalytic descriptor for Fe single-atom catalysts supported on C2N. J. Am. Chem. Soc. 2021, 143, 4405–4413. 10.1021/jacs.1c00889. 33705130

[ref163] Zhang C.; Dai Y.; Sun Q.; Ye C.; Lu R.; Zhou Y.; Zhao Y. Strategy to weaken the oxygen adsorption on single-atom catalysts towards oxygen-involved reactions. Materials Today Advances 2022, 16, 100280 10.1016/j.mtadv.2022.100280.

[ref164] Rosli R.; Sulong A.; Daud W.; Zulkifley M.; Husaini T.; Rosli M.; Majlan E.; Haque M. A review of high-temperature proton exchange membrane fuel cell (HT-PEMFC) system. Int. J. Hydrogen Energy 2017, 42, 9293–9314. 10.1016/j.ijhydene.2016.06.211.

[ref165] Jiao K.; Xuan J.; Du Q.; Bao Z.; Xie B.; Wang B.; Zhao Y.; Fan L.; Wang H.; Hou Z.; Huo S.; Brandon N. P.; Yin Y.; Guiver M. D. Designing the next generation of proton-exchange membrane fuel cells. Nature 2021, 595, 361–369. 10.1038/s41586-021-03482-7. 34262215

[ref166] Bing Y.; Liu H.; Zhang L.; Ghosh D.; Zhang J. Nanostructured Pt-alloy electrocatalysts for PEM fuel cell oxygen reduction reaction. Chem. Soc. Rev. 2010, 39, 2184–2202. 10.1039/b912552c. 20502804

[ref167] Čolic V.; Bandarenka A. S. Pt Alloy Electrocatalysts for the Oxygen Reduction Reaction: From Model Surfaces to Nanostructured Systems. ACS Catal. 2016, 6, 5378–5385. 10.1021/acscatal.6b00997.

[ref168] Wang X.; Li Z.; Qu Y.; Yuan T.; Wang W.; Wu Y.; Li Y. Review of Metal Catalysts for Oxygen Reduction Reaction: From Nanoscale Engineering to Atomic Design. Chem. 2019, 5, 1486–1511. 10.1016/j.chempr.2019.03.002.

[ref169] Lim C.; Fairhurst A. R.; Ransom B. J.; Haering D.; Stamenkovic V. R. Role of Transition Metals in Pt Alloy Catalysts for the Oxygen Reduction Reaction. ACS Catal. 2023, 13, 14874–14893. 10.1021/acscatal.3c03321. 38026811 PMC10660348

[ref170] Gracia J.; Fianchini M.; Biz C.; Polo V.; Gomez R. Spin polarisation in dual catalysts for the oxygen evolution and reduction reactions. Current Opinion in Electrochemistry 2021, 30, 100798 10.1016/j.coelec.2021.100798.

[ref171] Pavarini E. Solving the strong-correlation problem in materials. La Rivista del Nuovo Cimento 2021, 44, 597–640. 10.1007/s40766-021-00025-8.

[ref172] Capdevila-Cortada M.; Łodziana Z.; López N. Performance of DFT+ U approaches in the study of catalytic materials. ACS Catal. 2016, 6, 8370 10.1021/acscatal.6b01907.

[ref173] Vitillo J. G.; Cramer C. J.; Gagliardi L. Multireference methods are realistic and useful tools for modeling catalysis. Isr. J. Chem. 2022, 62, e202100136 10.1002/ijch.202100136.

[ref174] Smith R. C. Uncertainty quantification: theory, implementation, and applications; SIAM, 2014.

[ref175] Coveney P. V.; Highfield R. R. When we can trust computers (and when we can’t). Philosophical Transactions of the Royal Society A 2021, 379, 20200067 10.1098/rsta.2020.0067. PMC8059589 33775149

[ref176] Blais C. J.; Xu C.; West R. H. Uncertainty Quantification of Linear Scaling, Machine Learning, and Density Functional Theory Derived Thermodynamics for the Catalytic Partial Oxidation of Methane on Rhodium. J. Phys. Chem. C 2024, 128 (41), 17418 10.1021/acs.jpcc.4c05107. PMC11492380 39439883

[ref177] Wittreich G. R.; Gu G. H.; Robinson D. J.; Katsoulakis M. A.; Vlachos D. G. Uncertainty quantification and error propagation in the enthalpy and entropy of surface reactions arising from a single DFT functional. J. Phys. Chem. C 2021, 125, 18187–18196. 10.1021/acs.jpcc.1c04754.

[ref178] Wan S.; Sinclair R. C.; Coveney P. V. Uncertainty quantification in classical molecular dynamics. Philosophical Transactions of the Royal Society A 2021, 379, 20200082 10.1098/rsta.2020.0082. PMC8059622 33775140

[ref179] Yonge A.; Gusmão G. S.; Batchu R.; Kunz M. R.; Fang Z.; Fushimi R.; Medford A. J. Quantifying the impact of temporal analysis of products reactor initial state uncertainties on kinetic parameters. AIChE J. 2022, 68, e17776 10.1002/aic.17776.

[ref180] Boudart M. Turnover rates in heterogeneous catalysis. Chem. Rev. 1995, 95, 661–666. 10.1021/cr00035a009.

[ref181] Davis R. Turnover rates on complex heterogeneous catalysts. AIChE J. 2018, 64, 3778 10.1002/aic.16385.

[ref182] Anantharaj S.; Karthik P. E.; Noda S. The significance of properly reporting turnover frequency in electrocatalysis research. Angew. Chem., Int. Ed. 2021, 60, 23051–23067. 10.1002/anie.202110352. PMC8596788 34523770

[ref183] Zhang J. Modern Monte Carlo methods for efficient uncertainty quantification and propagation: A survey. Wiley Interdisciplinary Reviews: Computational Statistics 2021, 13, e1539 10.1002/wics.1539.

[ref184] Sudret B.; Marelli S.; Wiart J. Surrogate models for uncertainty quantification: An overview. 2017 11th European conference on antennas and propagation (EUCAP) 2017, 793–797. 10.23919/EuCAP.2017.7928679.

[ref185] Filot I. Introduction to microkinetic modeling, Ver. 1.8.1; Technische Universiteit: Eindhoven, 2022.

[ref186] Baker D. N. Microsoft and Johnson Matthey join forces to speed up hydrogen fuel cell innovation with Azure Quantum. Microsoft Azure Quantum Blog. 2023. https://cloudblogs.microsoft.com/quantum/2023/04/13/microsoft-and-johnson-matthey-join-forces-to-speed-up-hydrogen-fuel-cell-innovation-with-azure-quantum/ (accessed 30-08-2023).

[ref187] Siegel J.. Azure Quantum Elements aims to compress 250 years of chemistry into the next 25; Microsoft, 2023. https://news.microsoft.com/source/features/innovation/azure-quantum-elements-chemistry-materials-science/ (accessed 30-08-2023).

[ref188] IBM. Envisioning a new wave in power. https://www.ibm.com/case-studies/daimler (accessed 30-08-2023).

[ref189] IBM. ExxonMobil strives to solve complex energy challenges. https://www.ibm.com/case-studies/exxonmobil (accessed 30-08-2023).

[ref190] IBM. How quantum computers could help design airplanes. https://research.ibm.com/blog/boeing-case-study (accessed 30-08-2023).

[ref191] Bryant D. Architecting molecules that redefine luminescence; IBM, 2021. https://www.ibm.com/case-studies/jsr-mitsubishi-keio/ (accessed 30-8-2023).

[ref192] Mitsubishi Chemical — IBM — ibm.com. https://www.ibm.com/case-studies/mitsubishi-chemical, [Accessed 30–08–2023].

[ref193] Quantum Technology and Application Consortium (QUTAC). https://www.qutac.de/quantum-technology-and-application-consortium-qutac-2/?lang=en (accessed 30-08-2023).

[ref194] QUTAC. BASF: How quantum computing can help develop chemical catalysts. https://www.qutac.de/basf-how-quantum-computing-can-help-develop-chemical-catalysts/?lang=en (accessed 30-08-2023).

[ref195] QUTAC. Boehringer Ingelheim: A question of time. 2021. https://www.qutac.de/boehringer-ingelheim-a-question-of-time/?lang/en (accessed 30-08-2023).

[ref196] Kiser M.; Schroeder A.; Anselmetti G.-L. R.; Kumar C.; Moll N.; Streif M.; Vodola D. Classical and quantum cost of measurement strategies for quantum-enhanced auxiliary field Quantum Monte Carlo. New J. Phys. 2024, 26, 033022 10.1088/1367-2630/ad2f67.

[ref197] Aspuru-Guzik A.; Dutoi A. D.; Love P. J.; Head-Gordon M. Simulated Quantum Computation of Molecular Energies. Science 2005, 309, 1704–1707. 10.1126/science.1113479. 16151006

[ref198] Childs A. M.; Wiebe N. Hamiltonian simulation using linear combinations of unitary operations. arXiv, February 27, 2012, 1202.5822, ver. 1. 10.48550/arXiv.1202.5822.

[ref199] Berry D. W.; Childs A. M.; Cleve R.; Kothari R.; Somma R. D. Simulating Hamiltonian Dynamics with a Truncated Taylor Series. Phys. Rev. Lett. 2015, 114, 090502 10.1103/PhysRevLett.114.090502. 25793789

[ref200] Gilyén A.; Su Y.; Low G. H.; Wiebe N. Quantum singular value transformation and beyond: exponential improvements for quantum matrix arithmetics. Proceedings of the 51st Annual ACM SIGACT Symposium on Theory of Computing 2019, 193–204. 10.1145/3313276.3316366.

[ref201] Martyn J. M.; Rossi Z. M.; Tan A. K.; Chuang I. L. Grand Unification of Quantum Algorithms. PRX Quantum 2021, 2, 040203 10.1103/PRXQuantum.2.040203.

[ref202] Loaiza I.; Khah A. M.; Wiebe N.; Izmaylov A. F. Reducing molecular electronic Hamiltonian simulation cost for linear combination of unitaries approaches. Quantum Science and Technology 2023, 8, 035019 10.1088/2058-9565/acd577.

[ref203] Preskill J. Quantum Computing in the NISQ era and beyond. Quantum 2018, 2, 79 10.22331/q-2018-08-06-79.

[ref204] Nielsen M. A.; Chuang I. L. Quantum computation and quantum information; Cambridge University Press, 2010.

[ref205] Jones N. C.; Whitfield J. D.; McMahon P. L.; Yung M.-H.; Van Meter R.; Aspuru-Guzik A.; Yamamoto Y. Faster quantum chemistry simulation on fault-tolerant quantum computers. New J. Phys. 2012, 14, 115023 10.1088/1367-2630/14/11/115023.

[ref206] Nützel L.; Gresch A.; Hehn L.; Marti L.; Freund R.; Steiner A.; Marciniak C. D.; Eckstein T.; Stockinger N.; Wolf S.; Monz T.; Kühn M.; Hartmann M. J. Solving an Industrially Relevant Quantum Chemistry Problem on Quantum Hardware. arXiv, August 20, 2024, 2408.10801, ver. 1. 10.48550/arXiv.2408.10801.

[ref207] van Dam W.; Mykhailova M.; Soeken M. Using azure quantum resource estimator for assessing performance of fault tolerant quantum computation. Proceedings of the SC’23 Workshops of The International Conference on High Performance Computing, Network, Storage, and Analysis 2023, 1414–1419. 10.1145/3624062.3624211.

[ref208] Otten M.; Kang B.; Fedorov D.; Lee J.-H.; Benali A.; Habib S.; Gray S. K.; Alexeev Y. QREChem: quantum resource estimation software for chemistry applications. Frontiers in Quantum Science and Technology 2023, 2, 1232624 10.3389/frqst.2023.1232624.

[ref209] Harrigan M. P.; Khattar T.; Yuan C.; Peduri A.; Yosri N.; Malone F. D.; Babbush R.; Rubin N. C. Expressing and Analyzing Quantum Algorithms with Qualtran. arXiv, September 6, 2024, 2409.04643, ver. 1. 10.48550/arXiv.2409.04643.

[ref210] qml.resource. PennyLane. https://docs.pennylane.ai/en/stable/code/qml_resource.html (accessed 11-11-2024).

[ref211] Weidman J. D.; Sajjan M.; Mikolas C.; Stewart Z. J.; Pollanen J.; Kais S.; Wilson A. K. Quantum computing and chemistry. Cell Reports Physical Science 2024, 5, 102105 10.1016/j.xcrp.2024.102105.

[ref212] Mantegazza O.; Pasqualini V. 2023 Quantum Open Source Survey; Unitary Fund, 2023. https://unitaryfund.github.io/survey-website/ (accessed 11-11-2024).

[ref213] Tilly J.; Chen H.; Cao S.; Picozzi D.; Setia K.; Li Y.; Grant E.; Wossnig L.; Rungger I.; Booth G. H.; Tennyson J. The Variational Quantum Eigensolver: A review of methods and best practices. Phys. Rep. 2022, 986, 1–128. 10.1016/j.physrep.2022.08.003.

[ref214] Fedorov D. A.; Peng B.; Govind N.; Alexeev Y. VQE method: a short survey and recent developments. Materials Theory 2022, 6, 2 10.1186/s41313-021-00032-6.

[ref215] Cerezo M.; Arrasmith A.; Babbush R.; Benjamin S. C.; Endo S.; Fujii K.; McClean J. R.; Mitarai K.; Yuan X.; Cincio L.; Coles P. J. Variational quantum algorithms. Nature Reviews Physics 2021, 3, 625–644. 10.1038/s42254-021-00348-9.

[ref216] Bharti K.; Cervera-Lierta A.; Kyaw T. H.; Haug T.; Alperin-Lea S.; Anand A.; Degroote M.; Heimonen H.; Kottmann J. S.; Menke T.; Mok W.-K.; Sim S.; Kwek L.-C.; Aspuru-Guzik A. Noisy intermediate-scale quantum algorithms. Rev. Mod. Phys. 2022, 94, 015004 10.1103/RevModPhys.94.015004.

[ref217] Jordan P.; Wigner E. Über das Paulische Äquivalenzverbot. (German) [On Pauli’s equivalence prohibition]. Zeitschrift fÃŒr Physik 1928, 47, 631–651. 10.1007/BF01331938.

[ref218] Bravyi S. B.; Kitaev A. Y. Fermionic Quantum Computation. Annals of Physics 2002, 298, 210–226. 10.1006/aphy.2002.6254.

[ref219] Bravyi S.; Gambetta J. M.; Mezzacapo A.; Temme K. Tapering off qubits to simulate fermionic Hamiltonians. arXiv, January 27, 2017, 1701.08213, ver. 1. 10.48550/arXiv.1701.08213.

[ref220] Sousa C.; Tosoni S.; Illas F. Theoretical approaches to excited-state-related phenomena in oxide surfaces. Chem. Rev. 2013, 113, 4456–4495. 10.1021/cr300228z. 23194313

[ref221] Guo Q.; Ma Z.; Zhou C.; Ren Z.; Yang X. Single molecule photocatalysis on TiO2 surfaces: Focus review. Chem. Rev. 2019, 119, 11020–11041. 10.1021/acs.chemrev.9b00226. 31503466

[ref222] Tan H. L.; Abdi F. F.; Ng Y. H. Heterogeneous photocatalysts: an overview of classic and modern approaches for optical, electronic, and charge dynamics evaluation. Chem. Soc. Rev. 2019, 48, 1255–1271. 10.1039/C8CS00882E. 30761395

[ref223] Dutta S.; Erchinger J. E.; Strieth-Kalthoff F.; Kleinmans R.; Glorius F. Energy transfer photocatalysis: exciting modes of reactivity. Chem. Soc. Rev. 2024, 53, 1068 10.1039/D3CS00190C. 38168974

[ref224] Zhang Y.; He S.; Guo W.; Hu Y.; Huang J.; Mulcahy J. R.; Wei W. D. Surface-plasmon-driven hot electron photochemistry. Chem. Rev. 2018, 118, 2927–2954. 10.1021/acs.chemrev.7b00430. 29190069

[ref225] Zhou L.; Swearer D. F.; Zhang C.; Robatjazi H.; Zhao H.; Henderson L.; Dong L.; Christopher P.; Carter E. A.; Nordlander P.; et al. Quantifying hot carrier and thermal contributions in plasmonic photocatalysis. Science 2018, 362, 69–72. 10.1126/science.aat6967. 30287657

[ref226] Zhang Z.; Zhang C.; Zheng H.; Xu H. Plasmon-driven catalysis on molecules and nanomaterials. Accounts of chemical research 2019, 52, 2506–2515. 10.1021/acs.accounts.9b00224. 31424904

[ref227] Cortés E.; Besteiro L. V.; Alabastri A.; Baldi A.; Tagliabue G.; Demetriadou A.; Narang P. Challenges in plasmonic catalysis. ACS Nano 2020, 14, 16202–16219. 10.1021/acsnano.0c08773. 33314905

[ref228] Dong Y.; Hu C.; Xiong H.; Long R.; Xiong Y. Plasmonic catalysis: New opportunity for selective chemical bond evolution. ACS Catal. 2023, 13, 6730–6743. 10.1021/acscatal.3c00937.

[ref229] McClean J. R.; Kimchi-Schwartz M. E.; Carter J.; de Jong W. A. Hybrid quantum-classical hierarchy for mitigation of decoherence and determination of excited states. Phys. Rev. A 2017, 95, 042308 10.1103/PhysRevA.95.042308.

[ref230] Nakanishi K. M.; Mitarai K.; Fujii K. Subspace-search variational quantum eigensolver for excited states. Phys. Rev. Res. 2019, 1, 033062 10.1103/PhysRevResearch.1.033062.

[ref231] Parrish R. M.; Hohenstein E. G.; McMahon P. L.; Martínez T. J. Quantum Computation of Electronic Transitions Using a Variational Quantum Eigensolver. Phys. Rev. Lett. 2019, 122, 230401 10.1103/PhysRevLett.122.230401. 31298869

[ref232] Higgott O.; Wang D.; Brierley S. Variational Quantum Computation of Excited States. Quantum 2019, 3, 156 10.22331/q-2019-07-01-156.

[ref233] Jones T.; Endo S.; McArdle S.; Yuan X.; Benjamin S. C. Variational quantum algorithms for discovering Hamiltonian spectra. Phys. Rev. A 2019, 99, 062304 10.1103/PhysRevA.99.062304.

[ref234] Liu J.; Wan L.; Li Z.; Yang J. Simulating Periodic Systems on a Quantum Computer Using Molecular Orbitals. J. Chem. Theory Comput. 2020, 16, 6904–6914. 10.1021/acs.jctc.0c00881. 33073565

[ref235] Manrique D. Z.; Khan I. T.; Yamamoto K.; Wichitwechkarn V.; Ramo D. M. Momentum-Space Unitary Coupled Cluster and Translational Quantum Subspace Expansion for Periodic Systems on Quantum Computers. arXiv, January 4, 2021, 2008.08694, ver. 2. 10.48550/arXiv.2008.08694.

[ref236] Yoshioka N.; Sato T.; Nakagawa Y. O.; Ohnishi Y.-y.; Mizukami W. Variational quantum simulation for periodic materials. Phys. Rev. Research 2022, 4, 013052 10.1103/PhysRevResearch.4.013052.

[ref237] Huang B.; Govoni M.; Galli G. Simulating the electronic structure of spin defects on quantum computers. PRX Quantum 2022, 3, 010339 10.1103/PRXQuantum.3.010339.

[ref238] Bonet-Monroig X.; Sagastizabal R.; Singh M.; O’Brien T. Low-cost error mitigation by symmetry verification. Phys. Rev. A 2018, 98, 062339 10.1103/PhysRevA.98.062339.

[ref239] Sagastizabal R.; Bonet-Monroig X.; Singh M.; Rol M. A.; Bultink C.; Fu X.; Price C.; Ostroukh V.; Muthusubramanian N.; Bruno A.; et al. Experimental error mitigation via symmetry verification in a variational quantum eigensolver. Phys. Rev. A 2019, 100, 010302 10.1103/PhysRevA.100.010302.

[ref240] Yoshioka N.; Hakoshima H.; Matsuzaki Y.; Tokunaga Y.; Suzuki Y.; Endo S. Generalized Quantum Subspace Expansion. Phys. Rev. Lett. 2022, 129, 020502 10.1103/PhysRevLett.129.020502. 35867434

[ref241] Yalouz S.; Senjean B.; Gunther J.; Buda F.; O’Brien T. E.; Visscher L. A state-averaged orbital-optimized hybrid quantum–classical algorithm for a democratic description of ground and excited states. Quantum Science and Technology 2021, 6, 024004 10.1088/2058-9565/abd334.

[ref242] Yalouz S.; Koridon E.; Senjean B.; Lasorne B.; Buda F.; Visscher L. Analytical Nonadiabatic Couplings and Gradients within the State-Averaged Orbital-Optimized Variational Quantum Eigensolver. J. Chem. Theory Comput. 2022, 18, 776–794. 10.1021/acs.jctc.1c00995. 35029988

[ref243] Koridon E.; Fraxanet J.; Dauphin A.; Visscher L.; O’Brien T. E.; Polla S. A hybrid quantum algorithm to detect conical intersections. Quantum 2024, 8, 1259 10.22331/q-2024-02-20-1259.

[ref244] Mohammadbagherpoor H.; Oh Y.-H.; Singh A.; Yu X.; Rindos A. J. Experimental challenges of implementing quantum phase estimation algorithms on ibm quantum computer. arXiv, March 18, 2019, 1903.07605, ver. 1. 10.48550/arXiv.1903.07605.

[ref245] Mohammadbagherpoor H.; Oh Y.-H.; Dreher P.; Singh A.; Yu X.; Rindos A. J. An improved implementation approach for quantum phase estimation on quantum computers. arXiv, October 23, 2019, 1910.11696, ver. 1. 10.48550/arXiv.1910.11696.

[ref246] Duan B.; Yuan J.; Yu C.-H.; Huang J.; Hsieh C.-Y. A survey on HHL algorithm: From theory to application in quantum machine learning. Phys. Lett. A 2020, 384, 126595 10.1016/j.physleta.2020.126595.

[ref247] de Wolf R. Quantum Computing: Lecture Notes. arXiv, January 16, 2023, 1907.09415, ver. 5. 10.48550/arXiv.1907.09415.

[ref248] Tang E. Quantum Principal Component Analysis Only Achieves an Exponential Speedup Because of Its State Preparation Assumptions. Phys. Rev. Lett. 2021, 127, 060503 10.1103/PhysRevLett.127.060503. 34420330

[ref249] Low G. H.; Yoder T. J.; Chuang I. L. Methodology of Resonant Equiangular Composite Quantum Gates. Phys. Rev. X 2016, 6, 041067 10.1103/PhysRevX.6.041067.

[ref250] Low G. H.; Chuang I. L. Optimal Hamiltonian Simulation by Quantum Signal Processing. Phys. Rev. Lett. 2017, 118, 010501 10.1103/PhysRevLett.118.010501. 28106413

[ref251] Low G. H.; Chuang I. L. Hamiltonian Simulation by Qubitization. Quantum 2019, 3, 163 10.22331/q-2019-07-12-163.

[ref252] Tong Y.; An D.; Wiebe N.; Lin L. Fast inversion, preconditioned quantum linear system solvers, fast Green’s-function computation, and fast evaluation of matrix functions. Phys. Rev. A 2021, 104, 032422 10.1103/PhysRevA.104.032422.

[ref253] Ralli A.; Greene-Diniz G.; Ramo D. M.; Fitzpatrick N. Calculating the Single-Particle Many-body Green’s Functions via the Quantum Singular Value Transform Algorithm. arXiv, July 25, 2023, 2307.13583, ver. 1. 10.48550/arXiv.2307.13583.

[ref254] Gharibian S.; Le Gall F. Dequantizing the quantum singular value transformation: hardness and applications to quantum chemistry and the quantum PCP conjecture. Proceedings of the 54th Annual ACM SIGACT Symposium on Theory of Computing 2022, 19–32. 10.1145/3519935.3519991.

[ref255] Toyoizumi K.; Yamamoto N.; Hoshino K. Hamiltonian simulation using the quantum singular-value transformation: Complexity analysis and application to the linearized Vlasov-Poisson equation. Phys. Rev. A 2024, 109, 012430 10.1103/PhysRevA.109.012430.

[ref256] Kratzer P.; Neugebauer J. The Basics of Electronic Structure Theory for Periodic Systems. Frontiers in Chemistry 2019, 7, 00106 10.3389/fchem.2019.00106. PMC6424870 30918889

[ref257] Babbush R.; Wiebe N.; McClean J.; McClain J.; Neven H.; Chan G. K.-L. Low-Depth Quantum Simulation of Materials. Phys. Rev. X 2018, 8, 011044 10.1103/PhysRevX.8.011044.

[ref258] Ivanov A. V.; Sünderhauf C.; Holzmann N.; Ellaby T.; Kerber R. N.; Jones G.; Camps J. Quantum computation for periodic solids in second quantization. Phys. Rev. Res. 2023, 5, 013200 10.1103/PhysRevResearch.5.013200.

[ref259] Zini M. S.; Delgado A.; dos Reis R.; Casares P. A. M.; Mueller J. E.; Voigt A.-C.; Arrazola J. M. Quantum simulation of battery materials using ionic pseudopotentials. Quantum 2023, 7, 1049 10.22331/q-2023-07-10-1049.

[ref260] Gujarati T. P.; Motta M.; Friedhoff T. N.; Rice J. E.; Nguyen N.; Barkoutsos P. K.; Thompson R. J.; Smith T.; Kagele M.; Brei M.; Jones B. A.; Williams K. Quantum computation of reactions on surfaces using local embedding. npj Quantum Information 2023, 9, 88 10.1038/s41534-023-00753-1.

[ref261] Paola C. D.; Plekhanov E.; Krompiec M.; Kumar C.; Marsili E.; Du F.; Weber D.; Krauser J. S.; Shishenina E.; Ramo D. M. Platinum-based Catalysts for Oxygen Reduction Reaction simulated with a Quantum Computer. arXiv, April 12, 2024, 2307.15823, ver. 2. 10.48550/arXiv.2307.15823.

[ref262] Romanova M.; Weng G.; Apelian A.; Vlček V. Dynamical downfolding for localized quantum states. npj Computational Materials 2023, 9, 126 10.1038/s41524-023-01078-5.

[ref263] Greene-Diniz G.; Manrique D. Z.; Sennane W.; Magnin Y.; Shishenina E.; Cordier P.; Llewellyn P.; Krompiec M.; RanČić M. J.; Muñoz Ramo D. Modelling carbon capture on metal-organic frameworks with quantum computing. EPJ. Quantum Technology 2022, 9, 37 10.1140/epjqt/s40507-022-00155-w.

[ref264] Liu J.; Fan Y.; Li Z.; Yang J. Quantum algorithms for electronic structures: basis sets and boundary conditions. Chem. Soc. Rev. 2022, 51, 3263–3279. 10.1039/D1CS01184G. 35352716

[ref265] Stella L.; Attaccalite C.; Sorella S.; Rubio A. Strong electronic correlation in the hydrogen chain: A variational Monte Carlo study. Phys. Rev. B 2011, 84, 245117 10.1103/PhysRevB.84.245117.

[ref266] Sinitskiy A. V.; Greenman L.; Mazziotti D. A. Strong correlation in hydrogen chains and lattices using the variational two-electron reduced density matrix method. J. Chem. Phys. 2010, 133, 014104 10.1063/1.3459059. 20614956

[ref267] Motta M.; et al. Ground-State Properties of the Hydrogen Chain: Dimerization, Insulator-to-Metal Transition, and Magnetic Phases. Phys. Rev. X 2020, 10, 031058 10.1103/PhysRevX.10.031058.

[ref268] Barkoutsos P. K.; Gonthier J. F.; Sokolov I.; Moll N.; Salis G.; Fuhrer A.; Ganzhorn M.; Egger D. J.; Troyer M.; Mezzacapo A.; Filipp S.; Tavernelli I. Quantum algorithms for electronic structure calculations: Particle-hole Hamiltonian and optimized wave-function expansions. Phys. Rev. A 2018, 98, 022322 10.1103/PhysRevA.98.022322.

[ref269] Lee J.; Huggins W. J.; Head-Gordon M.; Whaley K. B. Generalized unitary coupled cluster wave functions for quantum computation. J. Chem. Theory Comput. 2019, 15, 311–324. 10.1021/acs.jctc.8b01004. 30485748

[ref270] Grimsley H. R.; Economou S. E.; Barnes E.; Mayhall N. J. An adaptive variational algorithm for exact molecular simulations on a quantum computer. Nat. Commun. 2019, 10, 3007 10.1038/s41467-019-10988-2. 31285433 PMC6614426

[ref271] Sun Q. Source code for pyscf.gto.basis; PySCF, 2014. https://pyscf.org/_modules/pyscf/gto/basis.html (accessed 07-02-2024).

[ref272] Goedecker S.; Teter M.; Hutter J. Separable dual-space Gaussian pseudopotentials. Phys. Rev. B 1996, 54, 1703–1710. 10.1103/PhysRevB.54.1703. 9986014

[ref273] Hartwigsen C.; Goedecker S.; Hutter J. Relativistic separable dual-space Gaussian pseudopotentials from H to Rn. Phys. Rev. B 1998, 58, 3641–3662. 10.1103/PhysRevB.58.3641. 9986014

[ref274] Musial M. Quantum Chemistry and Dynamics of Excited States; John Wiley & Sons, Ltd, 2020; pp 77–108.

[ref275] Stanton J. F.; Bartlett R. J. The equation of motion coupled-cluster method. A systematic biorthogonal approach to molecular excitation energies, transition probabilities, and excited state properties. J. Chem. Phys. 1993, 98, 7029–7039. 10.1063/1.464746.

[ref276] Mizuta K.; Fujii M.; Fujii S.; Ichikawa K.; Imamura Y.; Okuno Y.; Nakagawa Y. O. Deep variational quantum eigensolver for excited states and its application to quantum chemistry calculation of periodic materials. Phys. Rev. Research 2021, 3, 043121 10.1103/PhysRevResearch.3.043121.

[ref277] Fujii K.; Mizuta K.; Ueda H.; Mitarai K.; Mizukami W.; Nakagawa Y. O. Deep Variational Quantum Eigensolver: a divide-and-conquer method for solving a larger problem with smaller size quantum computers. arXiv, January 25, 2022, 2007.10917, ver. 2. 10.48550/arXiv.2007.10917.

[ref278] Yamamoto K.; Manrique D. Z.; Khan I. T.; Sawada H.; Ramo D. M. n. Quantum hardware calculations of periodic systems with partition-measurement symmetry verification: Simplified models of hydrogen chain and iron crystals. Phys. Rev. Res. 2022, 4, 033110 10.1103/PhysRevResearch.4.033110.

[ref279] Ostaszewski M.; Grant E.; Benedetti M. Structure optimization for parameterized quantum circuits. Quantum 2021, 5, 391 10.22331/q-2021-01-28-391.

[ref280] Sweke R.; Wilde F.; Meyer J.; Schuld M.; Faehrmann P. K.; Meynard-Piganeau B.; Eisert J. Stochastic gradient descent for hybrid quantum-classical optimization. Quantum 2020, 4, 314 10.22331/q-2020-08-31-314.

[ref281] Jackson C.; van Enk S. J. Detecting correlated errors in state-preparation-and-measurement tomography. Phys. Rev. A 2015, 92, 042312 10.1103/PhysRevA.92.042312.

[ref282] Tang F.; Wang L.; Walle M. D.; Mustapha A.; Liu Y.-N. An alloy chemistry strategy to tailoring the d-band center of Ni by Cu for efficient and selective catalytic hydrogenation of furfural. J. Catal. 2020, 383, 172–180. 10.1016/j.jcat.2020.01.019.

[ref283] Yan H.; Wang X.; Yao M.; Yao X. Band structure design of semiconductors for enhanced photocatalytic activity: The case of TiO2. Progress in Natural Science: Materials International 2013, 23, 402–407. 10.1016/j.pnsc.2013.06.002.

[ref284] Xiao J.; Han Q.; Cao H.; Rabeah J.; Yang J.; Guo Z.; Zhou L.; Xie Y.; Brückner A. Number of reactive charge carriers–a hidden linker between band structure and catalytic performance in photocatalysts. ACS Catal. 2019, 9, 8852–8861. 10.1021/acscatal.9b02426.

[ref285] Tasleem S.; Tahir M. Recent progress in structural development and band engineering of perovskites materials for photocatalytic solar hydrogen production: A review. Int. J. Hydrogen Energy 2020, 45, 19078–19111. 10.1016/j.ijhydene.2020.05.090.

[ref286] Wu B.; Zhang L.; Jiang B.; Li Q.; Tian C.; Xie Y.; Li W.; Fu H. Ultrathin porous carbon nitride bundles with an adjustable energy band structure toward simultaneous solar photocatalytic water splitting and selective phenylcarbinol oxidation. Angew. Chem., Int. Ed. 2021, 60, 4815–4822. 10.1002/anie.202013753. 33141452

[ref287] Cerasoli F. T.; Sherbert K.; Slawinska J.; Buongiorno Nardelli M. Quantum computation of silicon electronic band structure. Phys. Chem. Chem. Phys. 2020, 22, 21816–21822. 10.1039/D0CP04008H. 32966438

[ref288] Sherbert K.; Cerasoli F.; Buongiorno Nardelli M. A systematic variational approach to band theory in a quantum computer. RSC Adv. 2021, 11, 39438–39449. 10.1039/D1RA07451B. 35492501 PMC9044483

[ref289] Sherbert K.; Jayaraj A.; Nardelli M. B. Quantum algorithm for electronic band structures with local tight-binding orbitals. Sci. Rep. 2022, 12, 9867 10.1038/s41598-022-13627-x. 35701450 PMC9198033

[ref290] Sherbert K.; Nardelli M. B. Orthogonal-ansatz VQE: Locating excited states without modifying a cost-function. arXiv, April 9, 2022, 2204.04361, ver. 1. 10.48550/arXiv.2204.04361.

[ref291] Fan Y.; Liu J.; Li Z.; Yang J. Equation-of-Motion Theory to Calculate Accurate Band Structures with a Quantum Computer. J. Phys. Chem. Lett. 2021, 12, 8833–8840. 10.1021/acs.jpclett.1c02153. 34492184

[ref292] Szekeres Z.; Szabados Á.; Kállay M.; Surján P. R. On the “killer condition” in the equation-of-motion method: ionization potentials from multi-reference wave functions. Phys. Chem. Chem. Phys. 2001, 3, 696–701. 10.1039/b008428j.

[ref293] Lin C.; Zong F. H.; Ceperley D. M. Twist-averaged boundary conditions in continuum quantum Monte Carlo algorithms. Phys. Rev. E 2001, 64, 016702 10.1103/PhysRevE.64.016702. 11461437

[ref294] Liu J.; Matthews D. A.; Cheng L. Quadratic Unitary Coupled-Cluster Singles and Doubles Scheme: Efficient Implementation, Benchmark Study, and Formulation of an Extended Version. J. Chem. Theory Comput. 2022, 18, 2281–2291. 10.1021/acs.jctc.1c01210. 35312299

[ref295] Eddins A.; Motta M.; Gujarati T. P.; Bravyi S.; Mezzacapo A.; Hadfield C.; Sheldon S. Doubling the Size of Quantum Simulators by Entanglement Forging. PRX Quantum 2022, 3, 010309 10.1103/PRXQuantum.3.010309.

[ref296] Ryabinkin I. G.; Yen T.-C.; Genin S. N.; Izmaylov A. F. Qubit Coupled Cluster Method: ASystematic Approach to Quantum Chemistry on a Quantum Computer. J. Chem. Theory Comput. 2018, 14, 6317–6326. 10.1021/acs.jctc.8b00932. 30427679

[ref297] Krompiec M.; Ramo D. M. Strongly Contracted N-Electron Valence State Perturbation Theory Using Reduced Density Matrices from a Quantum Computer. arXiv, October 11, 2022, 2210.05702, ver. 1. 10.48550/arXiv.2210.05702.

[ref298] Sayfutyarova E. R.; Sun Q.; Chan G. K.-L.; Knizia G. Automated Construction of Molecular Active Spaces from Atomic Valence Orbitals. J. Chem. Theory Comput. 2017, 13, 4063–4078. 10.1021/acs.jctc.7b00128. 28731706

[ref299] Lau B. T. G.; Knizia G.; Berkelbach T. C. Regional Embedding Enables High-Level Quantum Chemistry for Surface Science. J. Phys. Chem. Lett. 2021, 12, 1104–1109. 10.1021/acs.jpclett.0c03274. 33475362

[ref300] Berry D. W.; Kieferová M.; Scherer A.; Sanders Y. R.; Low G. H.; Wiebe N.; Gidney C.; Babbush R. Improved techniques for preparing eigenstates of fermionic Hamiltonians. npj Quantum Information 2018, 4, 22 10.1038/s41534-018-0071-5.

[ref301] Poulin D.; Kitaev A.; Steiger D. S.; Hastings M. B.; Troyer M. Quantum algorithm for spectral measurement with a lower gate count. Physical review letters 2018, 121, 010501 10.1103/PhysRevLett.121.010501. 30028152

[ref302] Babbush R.; Gidney C.; Berry D. W.; Wiebe N.; McClean J.; Paler A.; Fowler A.; Neven H. Encoding electronic spectra in quantum circuits with linear T complexity. Physical Review X 2018, 8, 041015 10.1103/PhysRevX.8.041015.

[ref303] Berry D. W.; Gidney C.; Motta M.; McClean J. R.; Babbush R. Qubitization of arbitrary basis quantum chemistry leveraging sparsity and low rank factorization. Quantum 2019, 3, 208 10.22331/q-2019-12-02-208.

[ref304] Low G. H.; Chuang I. L. Hamiltonian simulation by qubitization. Quantum 2019, 3, 163 10.22331/q-2019-07-12-163.

[ref305] Kohn W. Analytic Properties of Bloch Waves and Wannier Functions. Phys. Rev. 1959, 115, 809–821. 10.1103/PhysRev.115.809.

[ref306] Ben Amor N.; Evangelisti S.; Leininger T.; Andrae D. In Basis Sets in Computational Chemistry; Perlt E., Ed.; Springer International Publishing: Cham, 2021; pp 41–101.

[ref307] Werner H.-J.; Knowles P. J. A second order multiconfiguration SCF procedure with optimum convergence. J. Chem. Phys. 1985, 82, 5053–5063. 10.1063/1.448627.

[ref308] Knowles P. J.; Werner H.-J. An efficient second-order MC SCF method for long configuration expansions. Chem. Phys. Lett. 1985, 115, 259–267. 10.1016/0009-2614(85)80025-7.

[ref309] Höyvik I.-M.; Jörgensen P. Characterization and Generation of Local Occupied and Virtual Hartree-Fock Orbitals. Chem. Rev. 2016, 116, 3306–3327. 10.1021/acs.chemrev.5b00492. 26855066

[ref310] Knizia G. Intrinsic Atomic Orbitals: An Unbiased Bridge between Quantum Theory and Chemical Concepts. J. Chem. Theory Comput. 2013, 9, 4834–4843. 10.1021/ct400687b. 26583402

[ref311] Senjean B.; Sen S.; Repisky M.; Knizia G.; Visscher L. Generalization of Intrinsic Orbitals to Kramers-Paired Quaternion Spinors, Molecular Fragments, and Valence Virtual Spinors. J. Chem. Theory Comput. 2021, 17, 1337–1354. 10.1021/acs.jctc.0c00964. 33555866

[ref312] Sen S.; Senjean B.; Visscher L. Characterization of excited states in time-dependent density functional theory using localized molecular orbitals. J. Chem. Phys. 2023, 158, 054115 10.1063/5.0137729. 36754801

[ref313] Pipek J.; Mezey P. G. A fast intrinsic localization procedure applicable for abinitio and semiempirical linear combination of atomic orbital wave functions. J. Chem. Phys. 1989, 90, 4916–4926. 10.1063/1.456588.

[ref314] Sen S.; Mascoli V.; Liguori N.; Croce R.; Visscher L. Understanding the Relation between Structural and Spectral Properties of Light-Harvesting Complex II. J. Phys. Chem. A 2021, 125, 4313–4322. 10.1021/acs.jpca.1c01467. 33979158 PMC8165694

[ref315] Sen S.; Visscher L. Towards the description of charge transfer states in solubilised LHCII using subsystem DFT. Photosynthesis Research 2023, 156, 39–57. 10.1007/s11120-022-00950-7. 35988131 PMC10070235

[ref316] ROSE. https://gitlab.com/quantum_rose/rose (accessed 04-03-2024).

[ref317] Georges A.; Kotliar G. Hubbard model in infinite dimensions. Phys. Rev. B 1992, 45, 6479–6483. 10.1103/PhysRevB.45.6479. 10000408

[ref318] Georges A.; Kotliar G.; Krauth W.; Rozenberg M. J. Dynamical mean-field theory of strongly correlated fermion systems and the limit of infinite dimensions. Rev. Mod. Phys. 1996, 68, 13–125. 10.1103/RevModPhys.68.13.

[ref319] Sun P.; Kotliar G. Extended dynamical mean-field theory and GW method. Phys. Rev. B 2002, 66, 085120 10.1103/PhysRevB.66.085120. 12906452

[ref320] Biermann S.; Aryasetiawan F.; Georges A. First-Principles Approach to the Electronic Structure of Strongly Correlated Systems: Combining the *GW* Approximation and Dynamical Mean-Field Theory. Phys. Rev. Lett. 2003, 90, 086402 10.1103/PhysRevLett.90.086402. 12633445

[ref321] Kotliar G.; Savrasov S. Y.; Haule K.; Oudovenko V. S.; Parcollet O.; Marianetti C. A. Electronic structure calculations with dynamical mean-field theory. Rev. Mod. Phys. 2006, 78, 865–951. 10.1103/RevModPhys.78.865.

[ref322] Georges A. The beauty of impurities: Two revivals of Friedel’s virtual bound-state concept. Comptes Rendus Physique 2016, 17, 430–446. 10.1016/j.crhy.2015.12.005.

[ref323] Paul A.; Birol T. Applications of DFT+ DMFT in materials science. Annu. Rev. Mater. Res. 2019, 49, 31–52. 10.1146/annurev-matsci-070218-121825.

[ref324] Zein N. E.; Savrasov S. Y.; Kotliar G. Local Self-Energy Approach for Electronic Structure Calculations. Phys. Rev. Lett. 2006, 96, 226403 10.1103/PhysRevLett.96.226403. 16803333

[ref325] Bauer B.; Wecker D.; Millis A. J.; Hastings M. B.; Troyer M. Hybrid Quantum-Classical Approach to Correlated Materials. Phys. Rev. X 2016, 6, 031045 10.1103/PhysRevX.6.031045.

[ref326] Rungger I.; Fitzpatrick N.; Chen H.; Alderete C. H.; Apel H.; Cowtan A.; Patterson A.; Ramo D. M.; Zhu Y.; Nguyen N. H.; Grant E.; Chretien S.; Wossnig L.; Linke N. M.; Duncan R. Dynamical mean field theory algorithm and experiment on quantum computers. arXiv, January 8, 2020, 1910.04735, ver. 2. 10.48550/arXiv.1910.04735.

[ref327] Suzuki Y.; et al. Qulacs: a fast and versatile quantum circuit simulator for research purpose. Quantum 2021, 5, 559 10.22331/q-2021-10-06-559.

[ref328] Sakurai R.; Mizukami W.; Shinaoka H. Hybrid quantum-classical algorithm for computing imaginary-time correlation functions. Phys. Rev. Res. 2022, 4, 023219 10.1103/PhysRevResearch.4.023219.

[ref329] Ma H.; Govoni M.; Galli G. Quantum simulations of materials on near-term quantum computers. npj Computational Materials 2020, 6, 85 10.1038/s41524-020-00353-z.

[ref330] Aryasetiawan F.; Imada M.; Georges A.; Kotliar G.; Biermann S.; Lichtenstein A. I. Frequency-dependent local interactions and low-energy effective models from electronic structure calculations. Phys. Rev. B 2004, 70, 195104 10.1103/PhysRevB.70.195104.

[ref331] Romanova M.; Vlček V. Decomposition and embedding in the stochastic GW self-energy. J. Chem. Phys. 2020, 153, 134103 10.1063/5.0020430. 33032410

[ref332] Romanova M.; Vlček V. Stochastic many-body calculations of moiré states in twisted bilayer graphene at high pressures. npj Computational Materials 2022, 8, 11 10.1038/s41524-022-00697-8.

[ref333] Ma H.; Govoni M.; Gygi F.; Galli G. A finite-field approach for GW calculations beyond the random phase approximation. J. Chem. Theory Comput. 2019, 15, 154–164. 10.1021/acs.jctc.8b00864. 30521333

[ref334] Ma H.; Govoni M.; Gygi F.; Galli G. Correction: A finite-field approach for GW calculations beyond the random phase approximation. J. Chem. Theory Comput. 2020, 16, 2877–2879. 10.1021/acs.jctc.0c00221. 32181645

[ref335] Nguyen N. L.; Ma H.; Govoni M.; Gygi F.; Galli G. Finite-Field Approach to Solving the Bethe-Salpeter Equation. Phys. Rev. Lett. 2019, 122, 237402 10.1103/PhysRevLett.122.237402. 31298883

[ref336] Wilson H. F.; Gygi F. m. c.; Galli G. Efficient iterative method for calculations of dielectric matrices. Phys. Rev. B 2008, 78, 113303 10.1103/PhysRevB.78.113303.

[ref337] Nguyen H.-V.; Pham T. A.; Rocca D.; Galli G. Improving accuracy and efficiency of calculations of photoemission spectra within the many-body perturbation theory. Phys. Rev. B 2012, 85, 081101 10.1103/PhysRevB.85.081101.

[ref338] Pham T. A.; Nguyen H.-V.; Rocca D.; Galli G. *GW* calculations using the spectral decomposition of the dielectric matrix: Verification, validation, and comparison of methods. Phys. Rev. B 2013, 87, 155148 10.1103/PhysRevB.87.155148.

[ref339] Govoni M.; Galli G. Large scale GW calculations. J. Chem. Theory Comput. 2015, 11, 2680–2696. 10.1021/ct500958p. 26575564

[ref340] Ma H.; Sheng N.; Govoni M.; Galli G. Quantum embedding theory for strongly correlated states in materials. J. Chem. Theory Comput. 2021, 17, 2116–2125. 10.1021/acs.jctc.0c01258. 33739106

[ref341] Temme K.; Bravyi S.; Gambetta J. M. Error Mitigation for Short-Depth Quantum Circuits. Phys. Rev. Lett. 2017, 119, 180509 10.1103/PhysRevLett.119.180509. 29219599

[ref342] Sheng N.; Vorwerk C.; Govoni M.; Galli G. Green’s function formulation of quantum defect embedding theory. J. Chem. Theory Comput. 2022, 18, 3512–3522. 10.1021/acs.jctc.2c00240. 35648660

[ref343] Stan A.; Dahlen N. E.; Van Leeuwen R. Levels of self-consistency in the GW approximation. J. Chem. Phys. 2009, 130, 114105 10.1063/1.3089567. 19317529

[ref344] Vorwerk C.; Sheng N.; Govoni M.; Huang B.; Galli G. Quantum embedding theories to simulate condensed systems on quantum computers. Nature Computational Science 2022, 2, 424–432. 10.1038/s43588-022-00279-0. 38177872

[ref345] Knizia G.; Chan G. K.-L. Density Matrix Embedding: A Simple Alternative to Dynamical Mean-Field Theory. Phys. Rev. Lett. 2012, 109, 186404 10.1103/PhysRevLett.109.186404. 23215304

[ref346] Knizia G.; Chan G. K.-L. Density matrix embedding: A strong-coupling quantum embedding theory. J. Chem. Theory Comput. 2013, 9, 1428–1432. 10.1021/ct301044e. 26587604

[ref347] Wouters S.; Jiménez-Hoyos C. A.; Sun Q.; Chan G. K.-L. A Practical Guide to Density Matrix Embedding Theory in Quantum Chemistry. J. Chem. Theory Comput. 2016, 12, 2706–2719. 10.1021/acs.jctc.6b00316. 27159268

[ref348] InQuanto. Quantinuum. https://www.quantinuum.com/computationalchemistry/inquanto (accessed 24-09-2023).

[ref349] Cao C.; Sun J.; Yuan X.; Hu H.-S.; Pham H. Q.; Lv D. Ab initio quantum simulation of strongly correlated materials with quantum embedding. npj Computational Materials 2023, 9, 78 10.1038/s41524-023-01045-0.

[ref350] Ralli A.; Williams de la Bastida M.; Coveney P. V. Scalable approach to quantum simulation via projection-based embedding. Phys. Rev. A 2024, 109, 022418 10.1103/PhysRevA.109.022418.

[ref351] HPCQC.org Home Page. https://www.hpcqc.org/home (accessed 19-01-2024).

[ref352] etp4hpc.eu. https://greencompute.uk/References/ETP4HPC/ETP4HPC_WP_Quantum4HPC_FINAL.pdf (accessed 19-01-2024).

[ref353] Schulz M.; Ruefenacht M.; Kranzlmuller D.; Schulz L. Accelerating HPC With Quantum Computing: It Is a Software Challenge Too. Computing in Science and Engineering 2022, 24, 60–64. 10.1109/MCSE.2022.3221845.38094600

[ref354] Piveteau C.; Sutter D. Circuit knitting with classical communication. IEEE Transactions on Information Theory 2024, 70 (4), 2734–2745. 10.1109/TIT.2023.3310797.

